# Effects of selenium supplementation on glycemic indices and oxidative-stress biomarkers in adults: a systematic review, meta-analysis, and dose-response analysis of randomized controlled trials

**DOI:** 10.1186/s12937-026-01376-w

**Published:** 2026-08-01

**Authors:** Xin-Yue Yang, Pan Xu, Xin Tan, Yu-Ting Sun, Min Tang, Chao Zhang, Long Wang, Shi-Shi Wu

**Affiliations:** 1 Center for Evidence-Based Medicine and Clinical Research, Taihe Hospital, Hubei University of Medicine, Shiyan, 442000 People’s Republic of China; 2 Department of Critical Care Medicine, Zhongnan Hospital of Wuhan University, Wuhan, 430071 People’s Republic of China; 3 Department of Pathology, Taihe Hospital, Hubei University of Medicine, Shiyan, 442000 People’s Republic of China; 4 Department of Clinical Nutrition, Taihe Hospital, Hubei University of Medicine, Shiyan, 442000 People’s Republic of China; 5 Department of Histology and Embryology, School of Basic Medical Sciences, Hubei University of Medicine, Shiyan, 442000 People’s Republic of China

**Keywords:** Selenium supplementation, Insulin resistance, Antioxidant biomarkers, Glycemic control, Lipid profile, Oxidative stress, Meta-analysis

## Abstract

**Background:**

Diabetes and metabolic syndrome are major public health challenges closely linked to oxidative stress, inflammation, and impaired insulin signaling. Selenium, an essential trace element with antioxidant and immunomodulatory roles, has been proposed as an adjunctive strategy for metabolic regulation; however, evidence from randomized trials remains inconsistent. We evaluated the effects of selenium supplementation on glycemic indices, oxidative-stress-related biomarkers, lipid metabolism, and related metabolic indicators in adults, and explored potential effect modifiers.

**Methods:**

We searched PubMed, Embase, Cochrane CENTRAL, Web of Science, CINAHL, CNKI, WanFang, VIP, and SinoMed through 12 August 2025 for randomized controlled trials of oral selenium supplementation in adults, including pregnant women with gestational diabetes mellitus (GDM). Outcomes included fasting plasma glucose, glycated hemoglobin, fasting insulin, indices of insulin resistance and β-cell function, oxidative-stress-related and inflammatory biomarkers, lipid parameters, and anthropometric and hemodynamic indicators. Random-effects meta-analyses estimated weighted mean differences with 95% confidence intervals. Subgroup, dose-response, and sensitivity analyses were performed, and certainty of evidence was assessed using GRADE.

**Results:**

Fifty-two treatment arms involving 10,916 participants were included. Selenium supplementation reduced fasting insulin, HOMA-IR, and HOMA-β values and increased QUICKI, but showed no clear overall effect on fasting plasma glucose or glycated hemoglobin. Favorable changes were observed in selected antioxidant and inflammatory biomarkers, including increases in glutathione, total antioxidant capacity, superoxide dismutase, glutathione peroxidase, and serum selenium, and reductions in malondialdehyde and high-sensitivity C-reactive protein, with no clear effect on nitric oxide. Modest changes in lipid parameters were also observed, including higher high-density lipoprotein cholesterol and lower low-density lipoprotein cholesterol, triglycerides, and total cholesterol. Among anthropometric and hemodynamic outcomes, only diastolic blood pressure was reduced. Effects varied by dose, duration, participant characteristics, and geographical selenium exposure context.

**Conclusions:**

Selenium supplementation was associated with modest improvements in insulin-sensitivity markers, selected antioxidantand inflammatory biomarkers, and lipid parameters, while showing no clear overall effect on fasting plasma glucose or glycated hemoglobin. These findings may be most applicable to populations with marginal or low baseline selenium status and should be extrapolated cautiously to selenium-replete settings. Long-term, high-quality RCTs are needed to clarify baseline-status-specific effects, safe dose ranges, and long-term safety.

**Trial registration:**

PROSPERO CRD420251124852.

**Supplementary Information:**

The online version contains supplementary material available at https://doi.org/10.1186/s12937-026-01376-w.

## Background

Over recent decades, the global prevalence rates of diabetes and metabolic syndrome (MetS) have increased markedly, imposing a substantial and escalating burden on public health [1, 2]. Poor glycemic control is a key driver of type 2 diabetes mellitus (T2DM) progression and a major risk factor for cardiovascular disease (CVD) [3]. MetS—characterized by insulin resistance, impaired glucose regulation, hypertension, and dyslipidemia—approximately doubles the risk of CVD and increases the risk of developing T2DM by up to fivefold [4]. The prevalence of MetS varies widely across regions, ethnicities, and age groups, ranging from 10% to 84% [5]. These patterns highlight the urgent need for effective and accessible strategies to prevent and manage metabolic disorders.

Lifestyle modification and pharmacotherapy remain central to metabolic disease management. Increasingly, dietary supplements have been explored as adjunctive strategies, because of their ease of use and potential accessibility. Among these, selenium has attracted particular interest because of its distinct biological roles [6].

Selenium is an essential trace element with biological relevance to metabolic regulation because it is incorporated into selenoproteins that are involved in redox homeostasis, inflammatory signaling, and cellular stress responses [7–9]. Key selenoproteins, including glutathione peroxidases and thioredoxin reductases, contribute to antioxidant defense and may help limit oxidative damage and inflammation, two processes closely linked to insulin resistance and metabolic dysfunction. Experimental studies also suggest that selenium may influence glucose metabolism through modulation of oxidative stress, inflammatory responses, and insulin-related signaling, including PI3K/Akt signaling. These mechanisms provide a biological rationale for evaluating selenium supplementation as a potential adjunctive nutritional approach for metabolic disorders.

From an epidemiological perspective, selenium exposure varies substantially across geographic regions because of differences in soil selenium availability, dietary patterns, and food sources. Both insufficient and excessive selenium exposure have been associated with cardiometabolic outcomes, suggesting that the relationship between selenium status and metabolic health may be context-dependent and potentially nonlinear. This is particularly relevant because populations with low or marginal selenium status may respond to supplementation differently from selenium-replete populations. At the same time, observational studies and randomized trials have reported inconsistent findings, with some suggesting metabolic benefits and others raising concerns about potential adverse glycemic effects at higher exposure levels or with long-term supplementation.

Previous systematic reviews and meta-analyses have provided useful evidence but were limited by a smaller number of randomized controlled trials, incomplete assessment of oxidative-stress-related and inflammatory biomarkers, limited exploration of dose-response patterns, and insufficient evaluation of the certainty of evidence. Therefore, a comprehensive synthesis of randomized evidence is needed to clarify whether selenium supplementation affects glycemic indices, insulin-sensitivity markers, oxidative-stress-related biomarkers, lipid parameters, and related metabolic outcomes, and whether these effects vary by dose, duration, baseline metabolic status, and selenium exposure context.

In this context, we conducted a comprehensive systematic review, meta-analysis, and dose-response evaluation of RCTs to assess the effects of oral selenium supplementation on metabolic health in adults. Primary outcomes included glycemic indices—FPG, glycated hemoglobin (HbA1c), FINS, homeostatic model assessment of insulin resistance (HOMA-IR), homeostatic model assessment of β-cell function (HOMA-β), and QUICKI—and oxidative-stress-related and inflammatory biomarkers, including glutathione (GSH), total antioxidant capacity (TAC), malondialdehyde (MDA), nitric oxide (NO), superoxide dismutase (SOD), high-sensitivity C-reactive protein (hs-CRP), glutathione peroxidase (GSH-Px), and circulating selenium levels (Se). Secondary outcomes comprised lipid parameters [high-density lipoprotein cholesterol (HDL-C), low-density lipoprotein cholesterol (LDL-C), very-low-density lipoprotein cholesterol (VLDL-C), triglycerides (TG), and total cholesterol (TC)] and anthropometric or hemodynamic indicators [weight, body mass index (BMI), systolic blood pressure (SBP), diastolic blood pressure (DBP), and waist circumference (WC)]. We further explored whether effects varied by supplementation dose, intervention duration, baseline metabolic status, and selenium exposure context to inform more rational and individualized use of selenium in metabolic health research and practice.

## Methods

### Literature search

We conducted a systematic search of nine electronic databases—PubMed, Embase, Cochrane Central Register of Controlled Trials (CENTRAL), Web of Science, CINAHL (EBSCO), CNKI, WanFang Data, VIP, and SinoMed—from inception to 12 August 2025. Search terms covered selenium supplementation, glycemic indices, insulin resistance, oxidative stress, oxidative-stress-related biomarkers, and randomized controlled trials. The complete database-specific search strategies, including English and Chinese search terms, are provided in Additional file 1: Table S1.

No language restrictions were imposed during the search process. To maximize trial capture, we also manually screened the reference lists of relevant articles. The review protocol was prospectively registered in PROSPERO (registration number: CRD420251124852).

### Inclusion and exclusion criteria

Studies were included if they met all of the following criteria: (i) adult participants (≥ 18 years), including pregnant women with gestational diabetes mellitus (GDM), with no other specific population restrictions; (ii) the intervention consisted of oral selenium supplements or selenium yeast; (iii) the control group received placebo, no selenium supplementation, including the same background co-intervention without selenium. Trials with concomitant active interventions were eligible only when the same co-intervention was provided to both the selenium and control groups, so that the between-group contrast isolated the incremental effect of selenium; (iv) the study assessed the effects of selenium supplementation on at least one of the following outcomes: glycemic markers, oxidative-stress-related or inflammatory biomarkers, lipid parameters, or anthropometric or hemodynamic indicators; and (v) the study used a randomized controlled clinical trial design, either parallel or crossover.

Studies were excluded if: (i) they were animal studies; (ii) the exposure of interest was blood selenium status rather than selenium supplementation; (iii) outcome data were incomplete or not extractable; (iv) no selenium-free comparator group was available; (v) selenium was co-administered with another active intervention in the experimental group, but the same background intervention was not provided to the control group; (vi) the report was a duplicate publication, in which case the most recent or most complete article was retained; or (vii) the publication type was a conference abstract, thesis, letter, or other non-full-text report.

No restrictions were imposed on baseline glycemic or metabolic status, baseline selenium status, or comorbid conditions during study selection. These characteristics were extracted during data collection and were subsequently explored in subgroup analyses or considered when interpreting the findings whenever sufficient data were available. In particular, studies involving participants with T2DM or GDM were analyzed separately from non-diabetic populations to assess potential effect modification by baseline metabolic status.

A list of excluded studies with reasons for exclusion is provided in Additional file 2: Table S2.

Two reviewers (XYY and PX) independently screened titles and abstracts according to the predefined eligibility criteria. The full texts of potentially eligible articles were retrieved and independently assessed for final inclusion. Disagreements were resolved through discussion; when consensus could not be reached, a third reviewer (LW) was consulted.

### Data extraction

Two reviewers (XYY and XT) independently extracted data from all eligible studies using a predesigned, standardized form. Extracted information included study characteristics (first author, year of publication, region/country, sample size, numbers enrolled and analyzed, study design, and main outcomes), participant characteristics (baseline glycemic indices and other relevant clinical or demographic characteristics), intervention details (selenium compound, daily dose, total cumulative dose, and duration), outcomes (glycemic and insulin homeostasis biomarkers, oxidative-stress-related and inflammatory biomarkers, lipid parameters, anthropometric and hemodynamic indicators), adverse events and reasons for loss to follow-up.

When studies did not directly report the mean change from baseline and the corresponding standard deviation (SD), these values were derived according to the Cochrane Handbook for Systematic Reviews of Interventions [10]. Specifically, when means and SDs at both baseline and endpoint were available, the SD of the change was estimated as:

$$\:\mathrm{S}{\mathrm{D}}_{\mathrm{change}}=\sqrt{\mathrm{S}{\mathrm{D}}_{\mathrm{B}}^{2}+\mathrm{S}{\mathrm{D}}_{\mathrm{F}}^{2}-2\times\:\mathrm{R}\times\:\mathrm{S}{\mathrm{D}}_{\mathrm{B}}\times\:\mathrm{S}{\mathrm{D}}_{\mathrm{F}}}$$

where $\mathrm{S}{\mathrm{D}}_{\mathrm{B}}$ and $\mathrm{S}{\mathrm{D}}_{\mathrm{F}}$ are the baseline and final standard deviations, respectively, and *R* denotes the correlation coefficient between baseline and final measurements [10]. If *R* was not reported, an imputed value of 0.5 was used, consistent with established methodological practice [11].

When only the standard error (SE) was reported, the SD was calculated as: $\mathrm{SD}=\mathrm{SE}\times\sqrt{\mathrm N}$, where *N* is the sample size.

When numerical results were presented only in graphical form, data were extracted from figures using WebPlotDigitize (https://automeris.io/). All extracted data were cross-checked for accuracy. Any discrepancies between reviewers were resolved through discussion or, if necessary, consultation with a third reviewer (LW).

### Quality assessment of the studies

The methodological quality and risk of bias of the included trials were independently assessed by two authors (LW and XYY) using the Cochrane risk-of-bias tool and the Jadad scale. The following seven domains were evaluated: random sequence generation, allocation concealment, blinding of participants and personnel, blinding of outcome assessment, completeness of outcome data, selective outcome reporting, and other potential sources of bias. Each domain was judged as having a low, high, or unclear risk of bias based on the reported information.

According to the Jadad scoring system, trials with scores ≤ 4 were classified as lower quality and those with scores > 4 as higher quality. The risk-of-bias assessments for all included studies are summarized in Additional file 10: Figure S1 and Additional file 7: Table S7.

### Quantitative data synthesis

All statistical analyses were performed using Stata software (version 15.1; StataCorp, College Station, TX, USA). Continuous outcomes were summarized as weighted mean differences (WMDs) with 95% confidence intervals (CIs). For each trial, the net change in each outcome was calculated as the difference in change from baseline between the selenium and control groups.

When necessary, SDs were estimated from reported statistics using the methods described by Hozo et al. [12] and Simental-Mendia et al. [13]. Outcome units were harmonized across studies using appropriate conversions before pooling. According to underlying disease status, trial populations were stratified into two groups: (i) T2DM or gestational diabetes mellitus (GDM) and (ii) non-diabetic populations.

Statistical heterogeneity was assessed using Cochran’s Q test and the I² statistic. A random-effects model was applied when substantial heterogeneity was present (I² > 50%) or when Cochran’s Q test indicated heterogeneity (*P* < 0.10). A fixed-effect model was used only when I² ≤ 50% and *P* ≥ 0.10.

### Nonlinear dose-response analysis

Nonlinear dose-response relationships were explored between selenium dose and selected metabolic, oxidative-stress-related, inflammatory, and selenium-status outcomes when sufficient dose-specific data were available. We used robust error meta-regression (REMR) as a one-stage dose-response meta-regression framework based on inverse variance–weighted least-squares regression with cluster-robust standard errors at the study level. This approach was selected because dose-specific effect estimates derived from the same study may be correlated, whereas the within-study covariance structure is usually unavailable in published aggregate data.

Selenium dose was modeled as a continuous exposure. Potential nonlinearity was evaluated by incorporating restricted cubic spline terms with three knots within the REMR framework. Dose-specific fitted values and 95% confidence intervals were then obtained from the fitted models. The adequacy of dose-response evidence was judged according to the number of contributing studies or treatment arms, the number of distinct selenium dose levels, the observed dose range, and the distribution of data points across the dose spectrum. Estimates at the extremes of the dose distribution, particularly at higher selenium doses, were interpreted cautiously when supported by limited data.

### Subgroup analyses

To explore potential effect modifiers and sources of heterogeneity, we conducted prespecified subgroup analyses according to baseline metabolic status (T2DM/GDM vs. non-diabetic populations), country (Iran vs. other countries), geographic region (Asia vs. non-Asia), intervention duration (≤ 84 days vs. > 84 days), selenium dose (200 µg/day vs. other doses), and intervention context (selenium-alone trials vs. balanced co-intervention trials). These subgroup analyses were interpreted as exploratory, particularly when the number of studies within a subgroup was limited.

### Publication bias and sensitivity analysis

Publication bias and small-study effects were assessed by visual inspection of funnel plots. Egger’s weighted regression test and Begg’s rank correlation test were performed only for outcomes with at least 10 contributing studies. For outcomes with fewer than 10 studies, formal statistical tests for funnel plot asymmetry were not performed because of limited statistical power. To evaluate the robustness of the pooled estimates and the influence of individual studies, we performed leave-one-out sensitivity analyses by sequentially omitting each study and repeating the meta-analysis. Study quality was assessed using the Cochrane risk-of-bias tool and the Jadad scale and was incorporated into the GRADE certainty assessment. Separate study-quality subgroup analyses were not performed for all outcomes because many outcomes included too few studies to support stable stratified estimates.

### GRADE certainty of the body of evidence

The overall certainty of evidence for each outcome was evaluated using the GRADE (Grading of Recommendations Assessment, Development, and Evaluation) approach [14]. Because all included studies were randomized controlled trials, the certainty of evidence initially started as high and was then rated down, as appropriate, according to five domains: risk of bias, inconsistency, indirectness, imprecision, and other considerations.

Risk of bias was assessed using the Cochrane risk-of-bias tool and the Jadad scale. Inconsistency was judged according to the magnitude of statistical heterogeneity, including the I² statistic, the P value for Cochran’s Q test, consistency in the direction of effects, overlap of confidence intervals, and whether heterogeneity could be explained by prespecified subgroup or sensitivity analyses. Indirectness was assessed by evaluating the consistency between the included evidence and the review PICO question, including population, intervention, comparator, and outcome. Particular attention was paid to whether co-interventions were balanced between groups and whether the between-group contrast isolated the incremental effect of selenium.

Imprecision was assessed based on the total number of participants, the number of contributing studies, the optimal information size, and the width of the 95% confidence interval. Evidence was downgraded when the optimal information size was not met, when only a small number of studies contributed data, or when the 95% confidence interval crossed the line of no effect or included both clinically important benefit and no effect. Other considerations included potential publication bias, small-study effects, and dose-response evidence. Publication bias and small-study effects were evaluated by visual inspection of funnel plots and, when at least 10 studies were available, by Egger’s and Begg’s tests. Formal tests for funnel plot asymmetry were not performed for outcomes with fewer than 10 studies because of limited statistical power. Dose-response evidence was considered supportive but did not offset downgrading concerns when important limitations remained regarding inconsistency, imprecision, indirectness, or potential publication bias.

## Results

### Study selection and characteristics

**Fig. 1:**
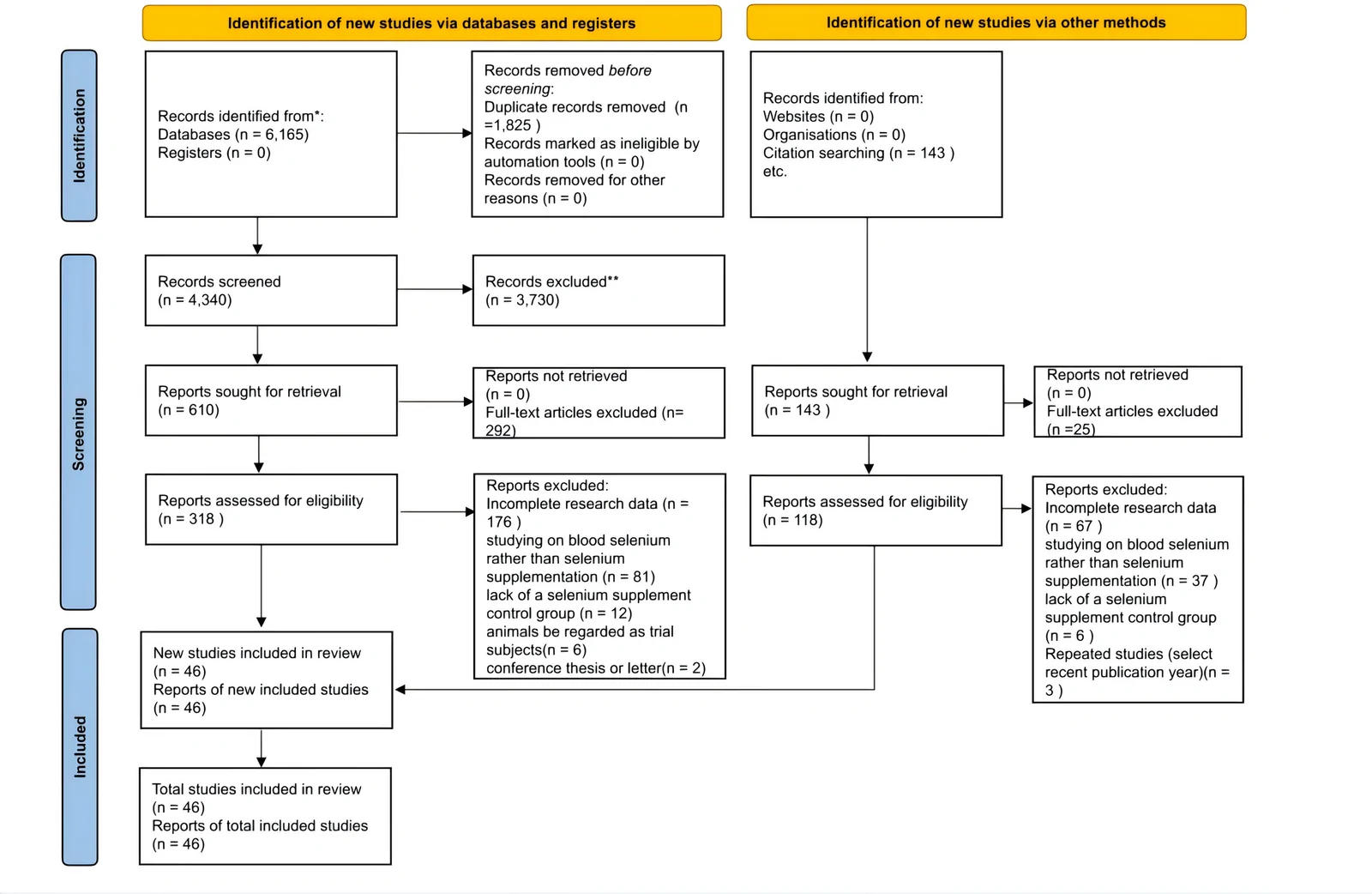
PRISMA 2020 flow diagram for the identification, screening, and inclusion of eligible studies

The study selection process is shown in Fig. 1. The database search identified 6,165 records, and 148 additional records were retrieved through citation searching. After removing 3,633 duplicates, 2,680 unique records were screened by title and abstract. Of these, 610 full-text articles were assessed for eligibility, and 46 reports met the inclusion criteria for the systematic review.

Four reports [15–18] each contained two independent selenium treatment arms, and one report [19] included three independent arms. Therefore, although the qualitative synthesis was based on 46 reports, the quantitative meta-analysis included 52 independent treatment arms.

The main characteristics of the included trials are summarized in Table 1. In total, 10,916 participants who completed follow-up were included in the quantitative synthesis. The included studies were published between 1998 and 2025. Among the 52 treatment arms, seven [20–26] enrolled patients with T2DM and six [27–32] included women with GDM. Daily selenium doses ranged from 10 to 960 µg/day (median 200 µg/day), and intervention durations ranged from 14 to 2,811 days (median 84 days).

**Table 1:** Characteristics of the included studies

References, public year, country	*N* (I/C)^1^	Participants		Intervention				Comparison	Outcome^2^	Study design
		FPG (mg/dL)	Population	Type of selenium compound	dosage (ug/day)	Duration (day)	total–dose (ug)			
Yan 2021 China [20]	83 (41/42)	110.16	T2DM + CLT	selenium supplement + levothyroxine sodium tablets	112	60	6720	levothyroxine sodium tablets	↑FPG	Parallel RCT NM
Meng 2022 China [27]	45 (24/21)	120.24	GDM	selenium supplement + regular hypoglycemic therapy	200	28	5600	regular hypoglycemic therapy	↓FPG, TC, TG, LDL-C;↔HOMA-IR, HDL-C	Parallel RCT NM
Li 2023 China [33]	76 (34/32)	88.20	PCOS	selenium supplement	60	84	5040	placebo	↑TG;↔FPG, FINS, HOMA-IR, TC, HDL-C, LDL-C	Parallel RCT double-blinded
Bahmani 2016 Iran [34]	60 (30/30)	ND	DN	selenium supplement	200	84	16,800	placebo	↓hs-CRP;↑GSH, TAC༛↔MDA, NO, HbA1C	Parallel RCT double-blinded
Amirani 2022 Iran [28]	60 (30/30)	91.50	GDM	selenium yeast + Probiotics	200	42	8400	probiotics	↓TC, TG, LDL-C, VLDL-C;↔FPG, FINS, HOMA-IR, QUICKI, HDL-C, Weight	Parallel RCT double-blinded
Salimian 2022 Iran [35]	60 (30/30)	107.90	HD	selenium supplement	200	168	33,600	placebo	↓hs-CRP, TC, LDL-C;↑GSH, TAC༛↔FPG, FINS, HOMA-IR, QUICKI, MDA, TG, HDL-C, VLDL-C, Weight, BMI	Parallel RCT double-blinded
Asemi 2015 Iran [31]	45 (20/20)	ND	PCOS + IVF	selenium supplement	200	56	11,200	placebo	↔Weight, BMI	Parallel RCT double-blinded
Zadeh 2022 Iran [36]	40 (20/20)	86.10	PCOS + IVF	selenium supplement	200	56	11,200	placebo	↓FPG, TAC, TC, TG, LDL-C;↑GSH༛↔FINS, HOMA-IR, QUICKI, MDA, HDL-C, VLDL-C, Weight, BMI	Parallel RCT double-blinded
Algotar 2013 United States [15]	465 (233/232)	102.30	CRPC	selenium supplement	400	1095	438,000	placebo	↑Se;↔FPG	Parallel RCT double-blinded
Mahdiyeh 2021 Iran [16]	60 (20/20)	115.17	AS	sodium selenite	200	56	11,200	placebo	↓FPG, FINS, TG;↔HOMA-IR, TC, HDL-C, LDL-C, SBP, DBP	Parallel RCT double-blinded
Najib 2019 Iran [30]	60 (30/30)	91.21	GDM	selenium supplement	100	84	13,200	placebo	↑FPG;↔FINS, HOMA-IR, HbA1C	Parallel RCT double-blinded
Mohammad 2016 Iran [37]	60 (30/30)	87.11	PCOS	selenium supplement	200	84	16,800	placebo	↔FPG, FINS, HOMA-IR	Parallel RCT double-blinded
Mesdaghinia 2017 Iran [38]	60 (30/30)	92.60	IUGR	selenium supplements	100	70	7000	placebo	↓HOMA-β, hs-CRP, LDL-C;↑GSH, TAC, HDL-C༛↔FPG, FINS, HOMA-IR, QUICKI, MDA, NO, TC, TG, VLDL-C, Weight, BMI	Parallel RCT double-blinded
Faghihi 2014 Iran [22]	65 (34/31)	150.34	T2DM	selenium supplement	200	90	18,000	placebo	↓TG;↑FPG, Se, HDL-C༛↔FINS, HOMA-IR, TC, LDL-C, HbA1C	Parallel RCT double-blinded
Rayman 2012 United Kingdom [19]	501 (126/121)	ND	SeD	selenium supplement	300	180	54,000	placebo	↑Se	Parallel RCT double-blinded
Farrokhian 2016 Iran [23]	60 (30/30)	133.90	T2DM	selenium supplement	200	56	11,200	placebo	↓FINS, HOMA-β, GSH, hs-CRP;↑FPG, TAC༛↔HOMA-IR, QUICKI, MDA, NO, TC, TG, HDL-C, LDL-C, VLDL-C	Parallel RCT double-blinded
Zhang 2014 China [24]	120 (60/60)	135.00	T2DM	selenium supplement + regular hypoglycemic therapy	200	360	72,000	regular hypoglycemic therapy	↓FPG; ↔HbA1C	Parallel RCT NM
Jia 2020 China [25]	96 (48/48)	105.48	T2DM + CLT	selenium supplement + levothyroxine sodium tablets	150	90	13,500	levothyroxine sodium tablets	↓FPG;↑Se; ↔HbA1C	Parallel RCT NM
Deng 2013 China [39]	94 (48/46)	ND	CLT	selenium supplement	200	180	36,000	placebo	↔HbA1C	Parallel RCT NM
Asemi 2015 Iran [31]	70 (35/35)	89.51	GDM	selenium supplement	200	42	8400	placebo	↓FPG, FINS, HOMA-β, hs-CRP, TG;↑GSH, TAC, NO༛↔HOMA-IR, QUICKI, MDA, TC, HDL-C, LDL-C, Weight, BMI, SBP, DBP	Parallel RCT double-blinded
Saifi 2020 Algerian [32]	180 (60/60)	80.00	GDM	selenium supplement	50	84	4200	placebo	↓FPG;↑Se༛↔MDA, BMI, SBP, DBP	Parallel RCT NM
Chen 2014 China [40]	64 (33/31)	120.78	IGT	selenium supplement + diet management	150	60	9000	diet management	↓FPG;↔BMI, HbA1C	Parallel RCT NM
Bahmani 2016 Iran [41]	80 (30/30)	122.40	DN	selenium supplement	200	84	16,800	placebo	↓HOMA-β, TG;↑TC, LDL-C༛↔FPG, FINS, HOMA-IR, QUICKI, HDL-C, VLDL-C, Weight, BMI	Parallel RCT double-blinded
Jamilian 2015 Iran [42]	75 (37/38)	92.70	PCOS	selenium supplement	200	56	11,200	placebo	↓FINS, HOMA-β, TG;↑LDL-C༛↔FPG, HOMA-IR, QUICKI, HDL-C, Weight, BMI	Parallel RCT double-blinded
Raygan 2018 Iran [43]	60 (30/30)	123.30	CHF	selenium supplement	200	84	16,800	placebo	↓FPG, hs-CRP, TC, LDL-C;↑GSH, TAC, HDL-C༛↔FINS, HOMA-IR, QUICKI, MDA, NO, TG, VLDL-C, Weight, BMI, SBP, DBP	Parallel RCT double-blinded
Kamali 2019 Iran [44]	40 (20/20)	120.20	CABG	selenium supplement	200	28	5600	placebo	↓FPG, hs-CRP;↑GSH, TAC༛↔FINS, HOMA-IR, QUICKI, MDA, NO, TC, TG, HDL-C, LDL-C, VLDL-C, Weight, BMI	Parallel RCT double-blinded
Gargari 2015 Iran [17]	34 (17/17)	91.80	CO	selenium supplement + HCD	200	42	8400	HCD	↑TG, SBP;↔FPG, FINS, HDL-C, WC, DBP	Parallel RCT double-blinded
Tamtaji 2019 Iran [45]	60 (30/30)	88.70	AD	selenium supplement	200	84	16,800	placebo	↓hs-CRP, TC, LDL-C;↑GSH, TAC༛↔FPG, FINS, HOMA-IR, QUICKI, NO, TG, HDL-C, VLDL-C, Weight, BMI	Parallel RCT double-blinded
Rashidi 2020 Iran [46]	76 (38/38)	ND	PCOS	selenium supplement	200	84	16,800	placebo	↓TG;↔TC, HDL-C, LDL-C	Parallel RCT double-blinded
Zamani 2023 Iran [47]	68 (34/34)	96.50	RA	selenium supplement	200	84	16,800	placebo	↓FPG, TAC, LDL-C;↑GSH, NO༛↔TC, TG, HDL-C	Parallel RCT double-blinded
Faure 2004 France [26]	48 (21/27)	156.20	T2DM	selenium supplement	960	90	86,400	placebo	↔FPG, FINS, HbA1C	Parallel RCT NM
Razavi 2016 Iran [48]	64 (32/32)	ND	PCOS	selenium supplement	200	56	11,200	placebo	↓GSH, TAC, hs-CRP;↔MDA, NO	Parallel RCT double-blinded
Mahmoodianfard 2015 Iran [49]	34 (17/17)	ND	BMI > 25 + HT	selenium supplement	200	84	16,800	placebo	↔Se, BMI	Parallel RCT double-blinded
Balali 2024 Iran [50]	72 (36/36)	ND	MH	selenium supplement	200	84	16,800	placebo	↑TAC;↔MDA, NO, BMI, WC, SBP, DBP	Parallel RCT double-blinded
Hu 2021 China [51]	90 (43/47)	ND	CLT	selenium supplement	200	180	36,000	placebo	↑Se	Parallel RCT double-blinded
Tian 2020 China [52]	32 (18/14)	ND	AIT	selenium supplement	200	90	18,000	placebo	↑SOD;↔TAC, MDA	Parallel RCT double-blinded
Adamowicz 2002 Poland [53]	22 (11/11)	ND	CRF	selenium supplement + vitamin E	128.6	90	11,571	vitamin E	↑SOD, GSH-Px, Se	Parallel RCT double-blinded
Atapour 2022 Iran [54]	88 (44/44)	ND	ESRD	selenium supplement + vitamin E	171.4	90	14,400	vitamin E	↓TC;↑Se༛↔TG, Weight	Parallel RCT double-blinded
Wu 2023 China [55]	92 (46/46)	ND	CLT	selenium supplement	200	180	36,000	placebo	↑SOD;↔MDA	Parallel RCT double-blinded
Zhou 2022 China [56]	80 (40/40)	ND	CLT	selenium supplement	200	180	36,000	placebo	↑SOD	Parallel RCT double-blinded
Qu 2019 China [57]	120 (60/60)	ND	CLT	selenium supplement	200	180	36,000	placebo	↑SOD	Parallel RCT non-blinded
Zhao 2000 China [58]	40 (20/20)	ND	CC	selenium supplement	600	14	8400	placebo	↑SOD, GSH-Px, Se;↔MDA	Parallel RCT non-blinded
Wang 2015 China [59]	120 (60/60)	109.44	A&O	selenium supplement	50	300	15,000	placebo	↔FPG	Parallel RCT double-blinded
Ye 2018 China [60]	84 (42/42)	ND	CU	selenium supplement + cetirizine tablets	200	56	11,200	cetirizine tablets	↓MDA	Parallel RCT non-blinded
XU 2012 China [61]	40 (20/20)	ND	AD	selenium supplement + huperzine A tablets	858	84	72,072	huperzine A tablets	↓hs-CRP;↑NO	Parallel RCT non-blinded
Rui 1998 China [18]	40 (20/20)	ND	CP	selenium supplement + chemotherapy	10	800	8000	chemotherapy	↑GSH-Px, Se	Parallel RCT non-blinded

*Abbreviations: T2DM *Type 2 Diabetes Mellitus, *CLT *Chronic Lymphocytic Thyroiditis, *GDM *Gestational Diabetes Mellitus, *PCOS *Polycystic Ovary Syndrome, *DN *Diabetic Nephropathy, *HD *Hemodialysis, *IVF *In Vitro Fertilization, *CRPC *Castration-Resistant Prostate Cancer, *AS *Atherosclerosis, *IUGR *Intrauterine Growth Restriction, *SeD *Selenium Deficiency (relative), *CHF *Congestive Heart Failure, *CABG *Coronary Artery Bypass Grafting, *CO *Central Obesity, *AD *Alzheimer’s Disease, *RA *Rheumatoid Arthritis, *BMI > 25 *Body Mass Index > 25 kg/m², *HT *Hypothyroidism, *MH *Migraine Headache, *AIT *Autoimmune Thyroiditis, *CRF *Chronic Renal Failure, *ESRD *End-Stage Renal Disease, *CC *Colorectal Cancer, *A&O *Adult and Older Adults (51–84 years old), *CU *Chronic Urticaria, *CP *Cancer Patients, *IGT *Impaired Glucose Tolerance, *ND *No Data

^1^N (I/C): T = total number of treated patients; I = number in the intervention group; C = number in the control group

^2^Outcome symbols indicate the direction of change in the selenium intervention group compared with controls: ↑ significant increase; ↔ no significant change; ↓ significant decrease

Reporting of glycemic outcomes varied across treatment arms: FPG was reported in 30 arms from28 reports [15–17, 20, 22–28, 30–33, 35–38, 40–45, 47, 59]; HbA1c in eight arms from 8 reports [22, 24–26, 30, 34, 39, 40]; FINS in 20 arms from 18 reports [16, 17, 22, 23, 28, 30, 31, 33, 35–37, 41–45, 62]; HOMA-IR in 19 arms from 18 reports [16, 17, 22, 23, 27, 28, 30, 31, 33, 35–38, 41–45]; HOMA-β in five arms from five reports [23, 31, 38, 41, 42]; and QUICKI in 11 arms from 11 reports [23, 28, 31, 35, 36, 38, 41–45].

Across the 46 reports (52 treatment arms), the original study populations comprised 11,287 participants, of whom 10,916 completed follow-up, corresponding to an overall attrition rate of 3.29% (371 withdrawals). Most withdrawals were due to participant-related reasons, including pregnancy, relocation, or non-adherence. In the control groups, 186 participants withdrew (154 for personal reasons and 32 due to adverse events, including 20 gastrointestinal events). In the selenium groups, 185 participants withdrew (145 for personal reasons and 40 due to adverse events, including 24 gastrointestinal events). Among studies reporting adverse events, the reported events were generally mild and predominantly gastrointestinal, such as flatulence and abdominal discomfort. However, safety reporting was incomplete across trials, and the available data were insufficient to draw firm conclusions regarding long-term safety.

### Serum selenium (Se)

**Fig. 2:**
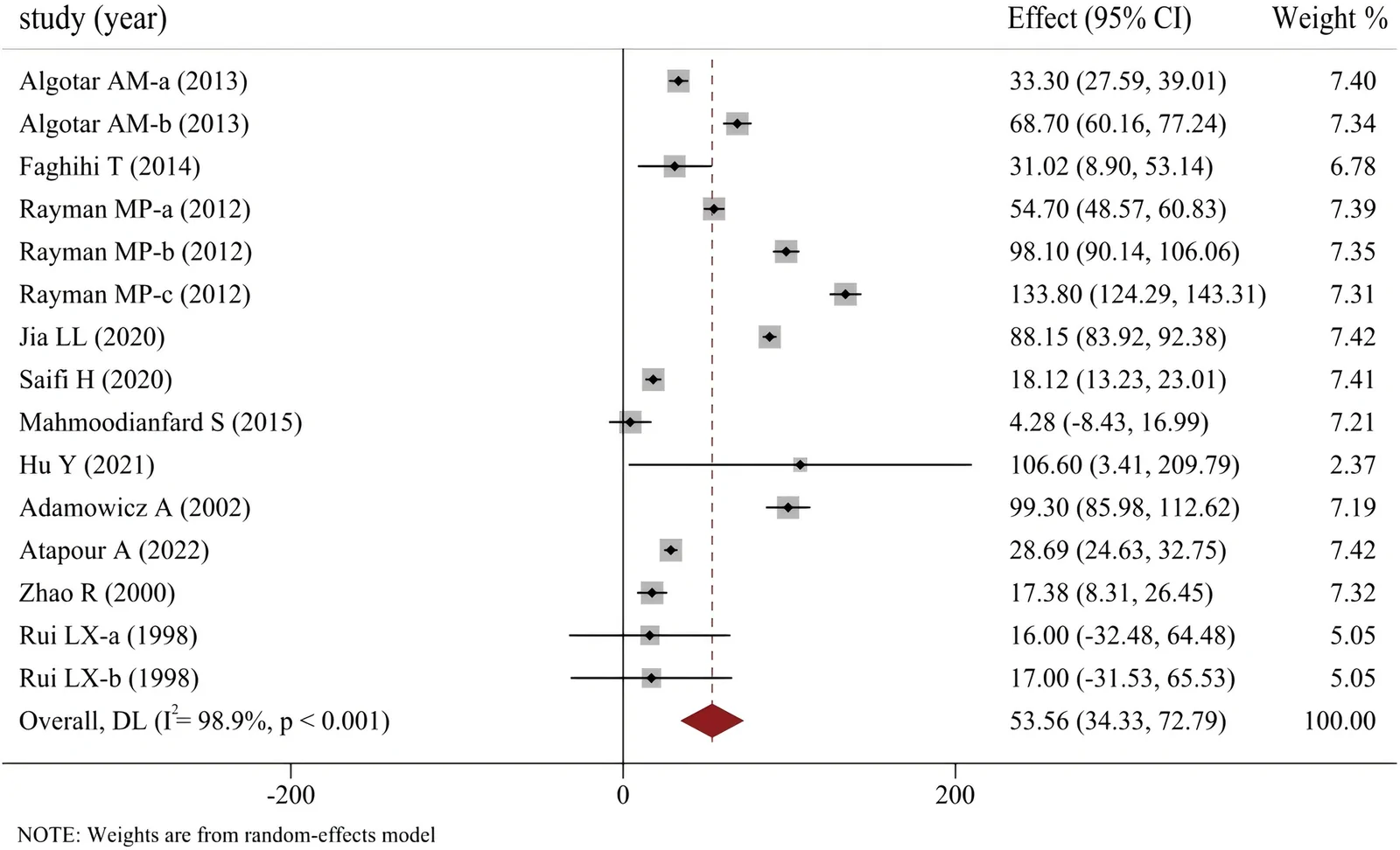
Effects of selenium supplementation on serum selenium levels. Between-group comparison of post-intervention serum selenium concentrations

The effect of selenium supplementation on serum Se was evaluated in 15 reports (15 treatment arms, *n* = 2,244). Overall, selenium supplementation markedly increased serum selenium compared with the control group (WMD = 53.56 µg/L; 95% CI: 34.33, 72.79; *P* < 0.001), with considerable heterogeneity (I² = 98.9%) (Table 2, Additional file 3: Table S3, Fig. 2).

**Table 2:** Meta-analysis results of selenium supplementation on glycemic and antioxidant outcomes

Indicators	Studies	I/C^1^	WMD (95% CI)	*P* ^2^	I^2^ (%)	*P* ^3^	Model	Egger’s Test		Begg’s Test		Quality of evidence
								t	*P* > |t|	z	Pr > |z|	
FPG (mg/dl)	30	1328/1327	-2.15 (-6.43,2.14)	0.326	94.6	< 0.001	random	2.32	0.028	-0.80	0.422	⨁◯◯◯ Very low
HbA1c (%)	8	295/293	-0.21 (-0.63,0.20)	0.317	96.4	< 0.001	random	NA	NA	NA	NA	⨁◯◯◯ Very low
FINS (µIU/ml)	20	506/511	-1.47 (-2.37,-0.57)	0.001	63.3	< 0.001	random	-1.06	0.305	-1.56	0.119	⨁⨁⨁◯ Moderate
HOMA-IR	19	492/488	-0.58 (-0.84,-0.31)	< 0.001	75.5	< 0.001	random	-1.42	0.172	-1.43	0.151	⨁⨁◯◯ Low
HOMA-β	5	160/160	-17.57 (-23.91,-11.23)	< 0.001	0.0	0.819	random	NA	NA	NA	NA	⨁⨁◯◯ Low
QUICKI	11	300/302	0.010 (0.006,0.015)	< 0.001	67.6	< 0.001	random	1.90	0.090	1.01	0.312	⨁⨁◯◯ Low
GSH (µmol/L)	11	296/300	55.87 (31.58,80.17)	< 0.001	42.9	0.064	random	-1.18	0.269	-1.79	0.073	⨁⨁⨁◯ Moderate
TAC (mmol/L)	13	350/350	23.76 (8.80,38.72)	0.002	79.5	< 0.001	random	-3.80	0.005	-1.70	0.089	⨁◯◯◯ Very low
MDA (µmol/L)	15	463/462	-1.08 (-1.62,-0.54)	< 0.001	96.4	< 0.001	random	0.32	0.756	0.74	0.458	⨁⨁◯◯ Low
NO (µmol/L)	11	306/309	0.53 (-1.32,2.38)	0.576	70.2	< 0.001	random	1.46	0.162	-0.13	0.897	⨁◯◯◯ Very low
SOD (U/ml)	6	195/191	15.48 (5.77,25.19)	0.002	94.7	< 0.001	random	NA	NA	NA	NA	⨁◯◯◯ Very low
GSH-Px (U/L)	4	71/71	32.37 (7.81,56.93)	0.010	97.3	< 0.001	random	NA	NA	NA	NA	⨁◯◯◯ Very low
hs-CRP (ng/ml)	10	267/270	-984.87 (-1419.65,-550.09)	< 0.001	79.2	< 0.001	random	-0.38	0.708	0.12	0.904	⨁◯◯◯ Very low
Se (ug/L)	15	1139/1105	53.56 (34.33,72.79)	< 0.001	98.9	< 0.001	random	-0.05	0.964	0.24	0.807	⨁⨁◯◯ Low

^1^I/C: I = number of patients in the intervention group; C = number of patients in the control group

^2^*P* value for subgroup differences between groups

^3^*P* value for heterogeneity within each subgroup

Subgroup analyses showed that selenium supplementation significantly increased serum selenium concentrations both non-diabetic and T2DM/GDM populations. The magnitude of increase appeared to vary by region, with larger increases observed in non-Asian regions (WMD = 72.09 µg/L; 95% CI: 42.88, 101.29) than in Asian regions (WMD = 34.23 µg/L; 95% CI: 4.38, 64.09). The magnitude of change also appeared to vary by intervention duration. Long interventions (> 84 days) produced greater increases (WMD = 64.96 µg/L; 95% CI: 43.92, 86.00) than shorter interventions (≤ 84 days; WMD = 15.10 µg/L; 95% CI: 8.24, 21.96). Dose stratification showed that both 200 µg/day (WMD = 47.87 µg/L; 95% CI: 7.80, 87.95) and other doses (WMD = 56.42 µg/L; 95% CI: 32.47, 80.37) significantly elevated serum selenium, suggesting that serum selenium increased across dose categories (Additional file 4: Table S4).

To further characterize changes within the intervention groups, we also conducted pre-post analyses within the selenium arms. Serum selenium increased significantly after supplementation (WMD = 51.39 µg/L; 95% CI: 32.42, 70.36; *P* < 0.001; I² = 98.9%) (Additional file 3: Table S3). The pattern of larger increases in non-T2DM/GDM participants, non-Asian regions (WMD = 72.76 µg/L; 95% CI: 42.80, 102.72), and longer interventions (> 84 days) was preserved, and both 200 µg/day and other doses were associated with significant within-group increases. Studies from Iran also showed significant increases, whereas the within-group change was not statistically significant in T2DM/GDM participants (Additional file 5: Table S5).

### Glycemic outcomes

#### Fasting Plasma Glucose (FPG)

**Fig. 3:**
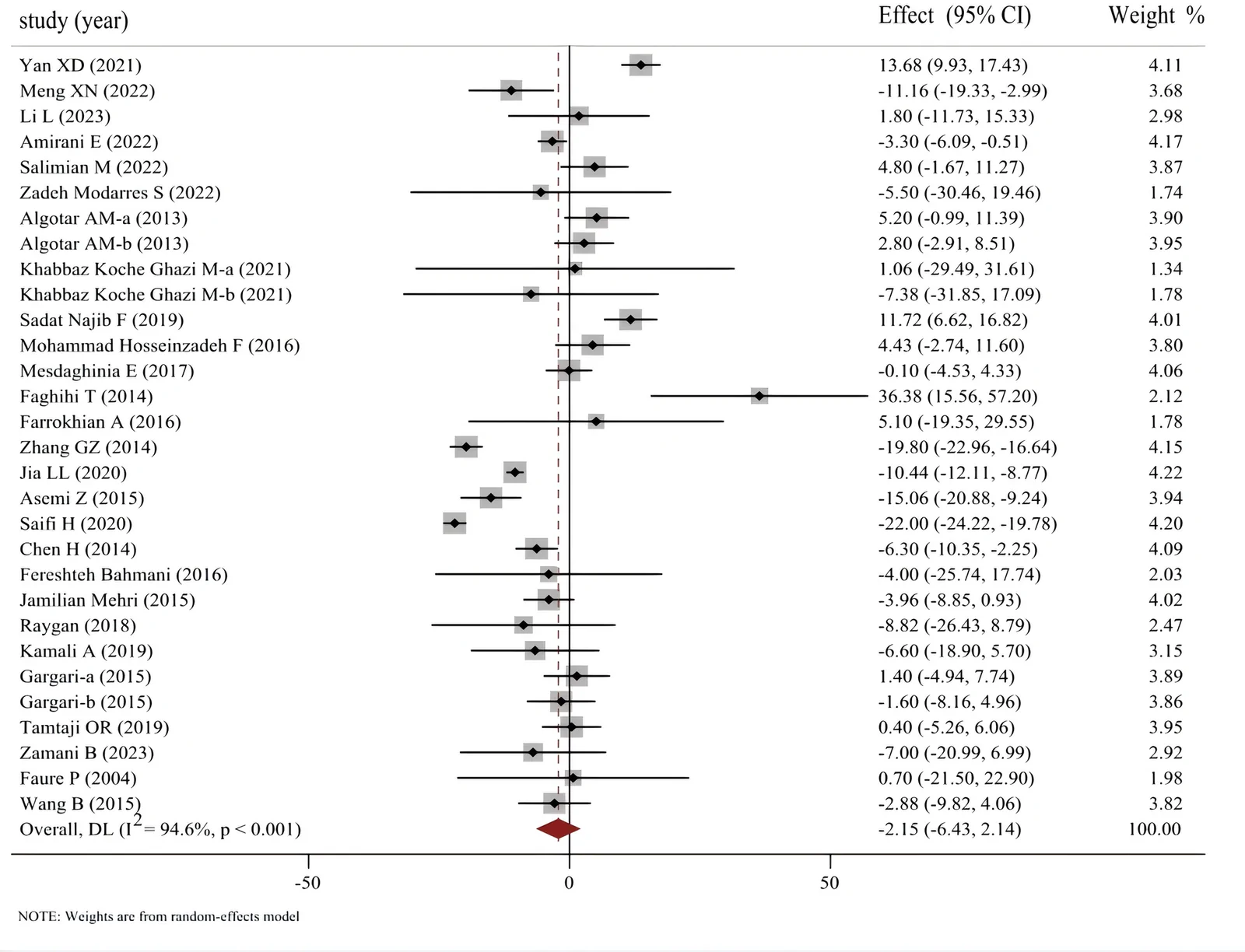
Effects of selenium supplementation on fasting plasma glucose. Between-group comparison of fasting plasma glucose (FPG)

Thirty treatment arms from 28 reports (*n* = 2,655) reported FPG. Compared with the control group, selenium supplementation was not associated with a significant change in FPG (WMD = − 2.15 mg/dL; 95% CI: −6.43, 2.14; *P* = 0.326), with substantial heterogeneity (I² = 94.6%; *P* < 0.001) (Table 2, Additional file 3: Table S3, Fig. 3). Subgroup analyses stratified by disease status, region, country, dose, and duration did not materially explain this heterogeneity (Table 4).

**Table 3:** Subgroup analyses of selenium effects on key glycemic indicators

Subgroups	FPG (mg/dL)				HbA1c (%)				FINS (µIU/mL)				HOMA-IR			
	*n*	WMD (95% CI)	*P* ^1^	I^2^(%)^2^	*n*	WMD (95% CI)	*P* ^1^	I^2^ (%)^2^	*n*	WMD (95% CI)	*P* ^1^	I^2^ (%)^2^	*n*	WMD (95% CI)	*P* ^1^	I^2^ (%)^2^
Population																
GDM/T2DM	11	-3.36 (-11.09,4.36)	0.394	97.6	5	-0.27 (-0.80,0.26)	0.322	96.4	6	-1.40 (-3.83,1.03)	0.258	80.1	6	-0.50 (-1.08,0.08)	0.089	83.7
Others	19	-0.61 (-2.57,1.35)	0.540	21.6	3	-0.10 (-0.22,0.00)	0.062	0.0	14	-1.54 (-2.54,-0.55)	0.002	51.1	13	-0.62 (-0.94,-0.29)	<0.001	72.0
Country																
Iran	19	-0.37 (-4.04,3.30)	0.845	75.0	3	0.22 (-0.63,1.07)	0.614	81.5	18	-1.83 (-2.76,-0.89)	<0.001	61.2	17	-0.65 (-0.96,-0.34)	<0.001	77.1
Other countries	11	-4.86 (-12.04,2.32)	0.184	97.2	5	-0.42 (-0.91,0.07)	0.096	97.5	2	1.16 (-0.55,2.86)	0.183	0.0	2	-0.26 (-0.86,0.35)	0.405	72.1
Region																
Asia	26	-1.88 (-6.01,2.24)	0.371	92.3	7	-0.23 (-0.67,0.21)	0.315	96.9	19	-1.62 (-2.54,-0.69)	0.001	63.5	19	-0.58 (-0.84,-0.31)	<0.001	75.5
Non-Asia	4	-3.75 (-21.61,14.10)	0.680	97.4	1	-0.09 (-1.02,0.84)	0.849	0.0	1	0.93 (-1.81,3.67)	0.507	0.0	-	-	-	-
Duration																
≤84(day)	22	-2.87 (-8.51,2.77)	0.319	94.7	3	-0.12 (-0.22,-0.01)	0.032	0.0	17	-2.05 (-3.05,-1.05)	<0.001	56.5	17	-0.68 (-0.96,-0.40)	<0.001	72.7
>84(day)	8	-0.55 (-8.11,7.01)	0.887	94.3	5	-0.23 (-0.79,0.32)	0.404	96.4	3	1.05 (-1.70,3.81)	0.453	77.6	2	0.93 (-1.67,3.53)	0.483	92.0
Dosage																
200(µg/d)	20	-2.76 (-7.49,1.98)	0.254	87.2	4	0.04 (-0.67,0.76)	0.903	88.9	16	-1.76 (-2.75,-0.77)	<0.001	63.8	16	-0.63 (-0.92,-0.33)	<0.001	77.8
Others	10	-1.34 (-9.42,6.74)	0.745	97.6	4	-0.42 (-1.13,0.30)	0.257	98.2	4	-0.45 (-2.72,1.82)	0.699	58.3	3	-0.36 (-1.05,0.34)	0.315	63.9

1*P* value for subgroup differences between groups

2I² indicates the percentage of variability across studies due to heterogeneity

#### Glycated hemoglobin (HbA1c)

**Fig. 4:**
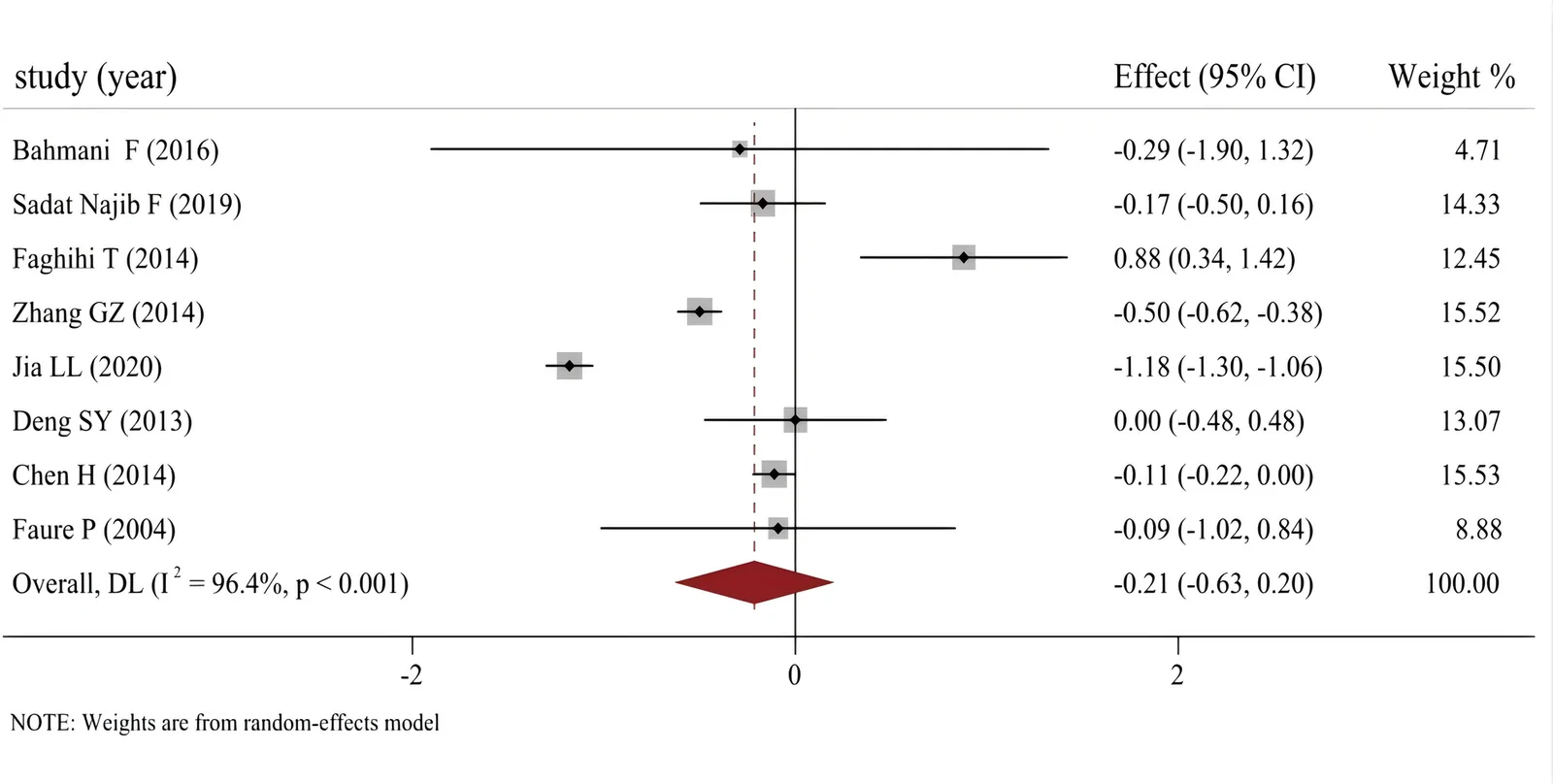
Effects of selenium supplementation on HbA1c. Between-group comparison of glycated hemoglobin (HbA1c)

Eight treatment arms from eight reports (*n* = 588) reported HbA1c. Between-group comparisons showed that selenium supplementation was not associated with a significant change in HbA1c (WMD = − 0.21%; 95% CI: −0.63, 0.20; *P* = 0.317), with very high heterogeneity (I² = 96.4%; *P* < 0.001) (Table 2, Additional file 3: Table S3, Fig. 4). Subgroup analyses did not identify clear effect modification by population type, region, dose, or duration (Table 4).

#### Fasting insulin (FINS)

Twenty treatment arms from 18 reports (*n* = 1,017) reported FINS. Compared with the control group, selenium supplementation was associated with a significant reduction in FINS (WMD = − 1.47 µIU/mL; 95% CI: −2.37, − 0.57; *P* = 0.001), with moderate heterogeneity (I² = 63.3%; *P* < 0.001) (Table 2, Additional file 3: Table S3).

The reduction was evident in non-diabetic populations (WMD = − 1.54 µIU/mL; 95% CI: −2.54, − 0.55; *P* = 0.002), but was not statistically significant in T2DM/GDM participants (WMD = − 1.40 µIU/mL; 95% CI: −3.83, 1.03; *P* = 0.258). Regionally, significant reductions were observed in studies from Iran (WMD = − 1.83 µIU/mL; 95% CI: −2.76, − 0.89; *P* < 0.001) and Asia overall (WMD = − 1.62 µIU/mL; 95% CI: −2.54, − 0.69; *P* = 0.001), but not in non-Asian regions.

By intervention duration, shorter trials (≤ 84 days) showed significant decreases in FINS (WMD = − 2.05 µIU/mL; 95% CI: −3.05, − 1.05; *P* < 0.001), whereas longer trials (> 84 days) showed no clear effect. A daily dose of 200 µg was associated with a significant reduction (WMD = − 1.76 µIU/mL; 95% CI: −2.75, − 0.77; *P* < 0.001), whereas other doses showed no clear effect (Table 4).

#### Homeostatic Model Assessment of Insulin Resistance (HOMA-IR)

Nineteen treatment arms from 18 reports (*n* = 980) reported HOMA-IR. Compared with the control group, selenium supplementation was associated with a significant reduction in HOMA-IR (WMD = − 0.58; 95% CI: −0.84, − 0.31; *P* < 0.001), with substantial heterogeneity (I² = 75.5%; *P* < 0.001) (Table 2, Additional file 3: Table S3).

The reduction appeared more evident in non-diabetic populations (WMD = − 0.62; 95% CI: −0.94, − 0.29; *P* < 0.001), whereas the effect was not statistically significant in T2DM/GDM participants (WMD = − 0.50; 95% CI: −1.08, 0.08; *P* = 0.089). Studies from Iran (WMD = − 0.65; 95% CI: −0.96, − 0.34; *P* < 0.001) and Asia overall (WMD = − 0.58; 95% CI: −0.84, − 0.31; *P* < 0.001) showed significant reductions in HOMA-IR, whereas non-Asian studies did not.

Short interventions (≤ 84 days) significantly decreased HOMA-IR (WMD = − 0.68; 95% CI: −0.96, − 0.40; *P* < 0.001); longer interventions (> 84 days) showed no statistically significant effect (WMD = 0.93; 95% CI: −1.67, 3.53; *P* = 0.483), with wide confidence intervals. A daily dose of 200 µg was associated with a significant reduction HOMA-IR (WMD = − 0.63; 95% CI: −0.92, − 0.33; *P* < 0.001), whereas other doses showed no clear effect (Table 4).

#### Homeostatic Model Assessment of β-cell function (HOMA-β)

Five treatment arms from five reports (*n* = 320) reported HOMA-β. Compared with the control group, selenium supplementation was associated with significantly lower HOMA-β values (WMD = − 17.57; 95% CI: −23.91, − 11.23; *P* < 0.001), with no detectable heterogeneity (I² = 0.0%; *P* = 0.819) (Table 2, Additional file 3: Table S3). Given the limited number of contributing studies, subgroup analyses for HOMA-β were considered exploratory and are presented only in Additional file 4: Table S4.

#### Quantitative Insulin Sensitivity Check Index (QUICKI)

Eleven treatment arms from11 reports (*n* = 602) reported QUICKI. Compared with the control group, selenium supplementation was associated with a small but statistically significant increase in QUICKI (WMD = 0.010; 95% CI: 0.006, 0.015; *P* < 0.001), with moderate heterogeneity (I² = 67.6%; *P* < 0.001) (Table 2, Additional file 3: Table S3).

However, in subgroup analyses, none of the strata defined by population type, region, dose, or duration showed a clearly significant effect, and heterogeneity remained largely unexplained (Additional file 4: Table S4).

### Oxidative-stress-related and inflammatory biomarkers

#### Glutathione (GSH)

Eleven treatment arms from11 reports (*n* = 596) reported GSH. Compared with the control group, selenium supplementation was associated with a significant increase in GSH (WMD = 55.87 µmol/L; 95% CI:31.58, 80.17; *P* < 0.001), with moderate heterogeneity (I² = 42.9%; *P* = 0.064) (Table 2, Additional file 3: Table S3).

The increase appeared more evident in non-diabetic populations (WMD = 56.48 µmol/L; 95% CI:30.33, 82.63) and in longer interventions (> 84 days; WMD = 69.90 µmol/L; 95% CI: 54.26, 85.54) than in shorter interventions (≤ 84 days; WMD = 50.25 µmol/L; 95% CI:18.73, 81.77). Trials using 200 µg/day also showed significant increases in GSH(WMD = 57.64 µmol/L; 95% CI: 29.91, 85.36). Studies from Asia and Iran showed consistently positive estimates (Table 4).

**Table 4:** Subgroup analyses of selenium effects on antioxidant markers

Subgroups	GSH (µmol/L)				TAC (mmol/L)				MDA (µmol/L)				NO (µmol/L)			
	*n*	WMD (95% CI)	*P* ^1^	I^2^(%)^2^	*n*	WMD (95% CI)	*P* ^1^	I^2^ (%)^2^	*n*	WMD (95% CI)	*P* ^1^	I^2^ (%)^2^	*n*	WMD (95% CI)	*P* ^1^	I^2^ (%)^2^
Population																
GDM/T2DM	2	46.25 (-52.09,144.60)	0.357	75.8	2	223.10 (-151.22,597.41)	0.243	91.6	3	-0.87 (-1.66,-0.08)	0.030	86.5	2	1.10 (-16.89,19.09)	0.905	49.8
Others	9	56.48 (30.33,82.63)	< 0.001	40.1	11	18.79 (5.35,32.2)	0.006	75.5	12	-1.15 (-1.79,-0.50)	< 0.001	96.4	9	0.73 (-1.16,2.62)	0.447	73.8
Country																
Iran	11	55.87 (31.58,80.17)	< 0.001	42.9	12	38.21 (8.95,67.47)	0.010	77.8	10	-0.27 (-0.44,-0.10)	0.002	36.3	10	-0.23 (-1.67,1.21)	0.751	49.4
Other countries	-	-	-	-	1	4.20 (2.35,6.05)	< 0.001	0.0	5	-2.26 (-3.67,-0.85)	0.002	97.3	1	7.11 (3.50,10.72)	< 0.001	0.0
Region																
Asia	11	55.87 (31.58,80.17)	< 0.001	42.9	13	23.76 (8.80,38.72)	0.002	79.5	14	-1.05 (-1.63,-0.48)	< 0.001	95.8	11	0.53 (-1.32,2.38)	0.576	70.2
Non-Asia	-	-	-	-	-	-	-	-	1	-1.48 (-1.68,-1.28)	< 0.001	0.0	-	-	-	-
Duration																
≤ 84(day)	10	50.25 (18.73,81.77)	0.002	44.1	11	44.92 (9.94,79.90)	0.012	79.8	12	-0.97 (-1.61,-0.33)	0.003	96.2	11	0.53 (-1.32,2.38)	0.576	70.2
> 84(day)	1	69.90 (54.26,85.54)	< 0.001	0.0	2	4.23 (2.38,6.08)	< 0.001	0.0	3	-1.57 (-3.44,0.30)	0.100	97.5	-	-	-	-
Dosage																
200(µg/d)	10	57.64 (29.91,85.36)	< 0.001	44.4	12	13.13 (0.61,25.66)	0.040	67.5	12	-1.22 (-1.92,-0.53)	0.001	96.4	9	-0.25 (-2.09,1.60)	0.794	54.8
Others	1	40.60 (-2.09,83.29)	0.062	0.0	1	86.60 (54.44,118.76)	< 0.001	0.0	3	-0.58 (-1.66,0.50)	0.293	97.1	2	3.27 (-3.88,10.43)	0.370	92.3

^1^*P* value for subgroup differences between groups

^2^I² indicates the percentage of variability across studies due to heterogeneity

#### Total Antioxidant Capacity (TAC)

**Fig. 5:**
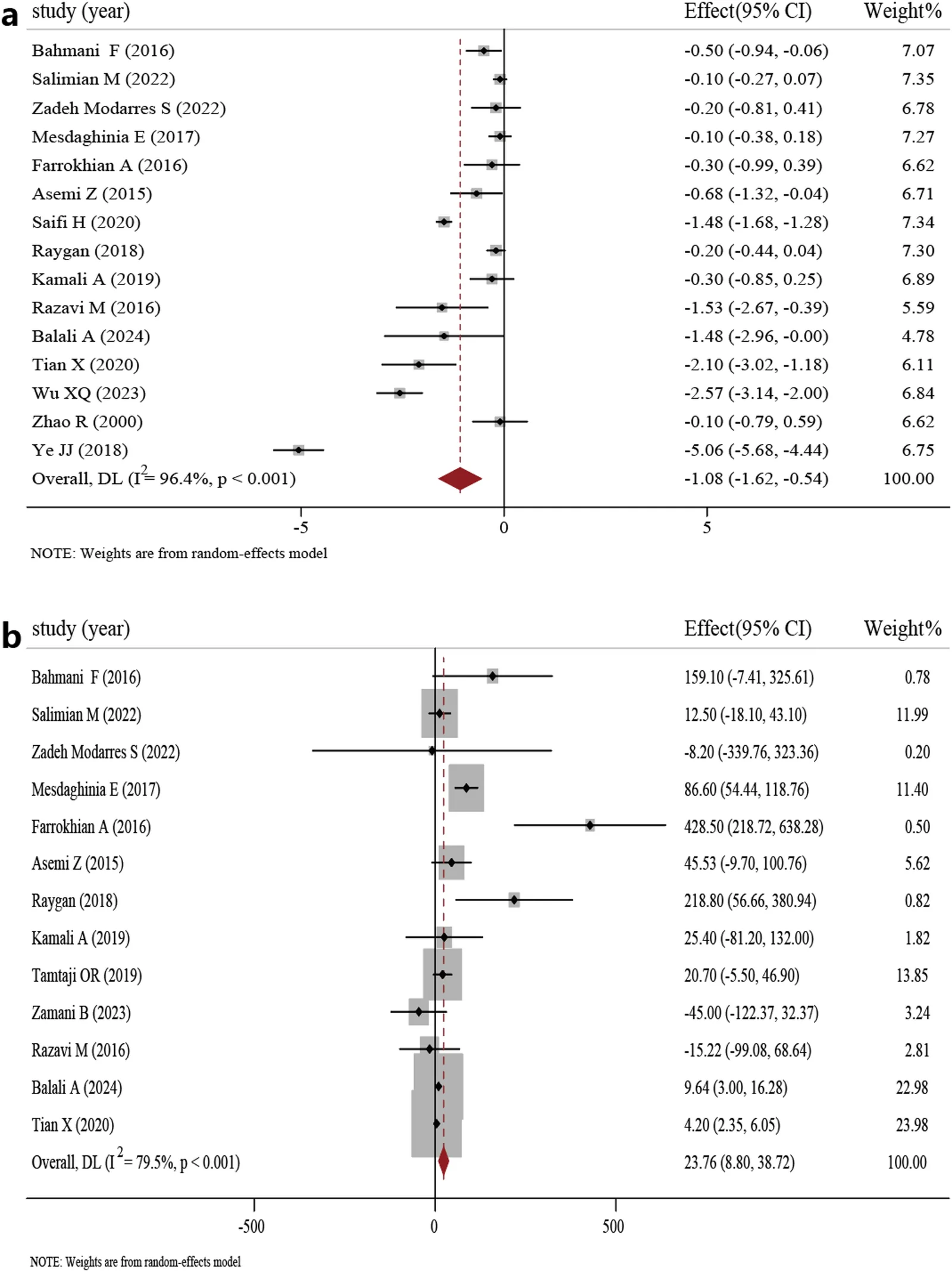
Effects of selenium on oxidative stress biomarkers. **a **Forest plot of malondialdehyde (MDA) concentrations (between-group comparison). **b **Forest plot of total antioxidant capacity (TAC) (between-group comparison)

Thirteen treatment arms from13 reports (*n* = 700) reported TAC. Compared with the control group, selenium supplementation was associated with a significant increase in TAC (WMD = 23.76 mmol/L; 95% CI: 8.80, 38.72; *P* = 0.002), with high heterogeneity (I² = 79.5%; *P* < 0.001) (Table 2, Additional file 3: Table S3, Fig. 5).

In subgroup analyses, the increase was more evident in non-diabetic populations (WMD = 18.79 mmol/L; 95% CI: 5.35, 32.20). In the T2DM/GDM subgroup, the TAC estimate was highly imprecise (WMD = 223.10 mmol/L; 95% CI: −151.22, 597.41) and was based on a limited number of studies. This subgroup estimate was retained for transparency but should not be interpreted as robust evidence of a population-specific effect.

A dose of 200 µg/day produced a modest but significant increase in TAC (WMD = 13.13 mmol/L; 95% CI: 0.61, 25.66), whereas other dose categories showed larger estimates (WMD = 86.60 mmol/L; 95% CI: 54.44, 118.76), although these results were based on limited data. Iranian trials also showed substantial increases in TAC (WMD = 38.21 mmol/L; 95% CI: 8.95, 67.47) (Table 4).

#### Malondialdehyde (MDA)

Fifteen treatment arms from 15 reports (*n* = 925) reported MDA. Compared with the control group, selenium supplementation was associated with a significant reduction in MDA (WMD = − 1.08 µmol/L; 95% CI: −1.62, − 0.54; *P* < 0.001), with very high heterogeneity (I² = 96.4%; *P* < 0.001) (Table 2, Additional file 3: Table S3, Fig. 5).

The reductions appeared somewhat larger in non-diabetic populations (WMD = − 1.15 µmol/L; 95% CI: −1.79, − 0.50) than in T2DM/GDM groups (WMD = − 0.87 µmol/L; 95% CI: −1.66, − 0.08). By intervention duration, the point estimate was larger for longer interventions (> 84 days; WMD = − 1.57 µmol/L; 95% CI: −3.44, 0.30) than for short interventions (≤ 84 days: WMD = − 0.97 µmol/L; 95% CI: −1.61, − 0.33), although the confidence interval for long interventions crossed the line of no effect. A dose of 200 µg/day was associated with a significant reduction in MDA (WMD = − 1.22 µmol/L; 95% CI: −1.92, − 0.53), whereas other doses showed no clear effect (WMD = − 0.58 µmol/L; 95% CI: −1.66, 0.50). Both Asian studies (WMD = − 1.05 µmol/L; 95% CI: −1.63, − 0.48) and non-Asian studies (WMD = − 1.48 µmol/L; 95% CI: −1.68, − 1.28) showed reductions in MDA (Table 4).

#### Nitric Oxide (NO)

Eleven treatment arms from11 reports (*n* = 615) reported NO. Compared with the control group, selenium supplementation was not associated with a significant change in NO concentrations (WMD = 0.53 µmol/L; 95% CI: −1.32, 2.38; *P* = 0.576), with high heterogeneity (I² = 70.2%; *P* < 0.001) (Table 2, Additional file 3: Table S3).

No statistically significant effects were observed in non-diabetic populations or in shorter interventions (≤ 84 days), and data were insufficient to draw firm conclusions for longer interventions. Trials using 200 µg/day and those using other doses also showed no significant effects, with no clear regional pattern across Asian and non-Asian studies (Table 4).

#### Superoxide Dismutase (SOD)

Six treatment arms from6 reports (*n* = 386) reported SOD. Compared with the control group, selenium supplementation was associated with a significant increase in SOD activity (WMD = 15.48 U/mL; 95% CI: 5.77, 25.19; *P* = 0.002), with very high heterogeneity (I² = 94.7%; *P* < 0.001) (Table 2, Additional file 3: Table S3).

In subgroup analyses, increased SOD activity was observed in non-diabetic populations (WMD = 15.48 U/mL; 95% CI: 5.77, 25.19). The increase appeared greater in longer interventions (> 84 days; WMD = 16.87 U/mL; 95% CI: 5.79, 27.95) than in shorter interventions (≤ 84 days; WMD = 8.60 U/mL; 95% CI: −0.87, 18.07), although the latter was not statistically significant. A dose of 200 µg/day was associated with a significant increase in SOD activity (WMD = 17.58 U/mL; 95% CI: 5.97, 29.19), whereas other doses showed no clear effect (WMD = 8.56 U/mL; 95% CI: −0.57, 17.68). Asian trials showed significant increases (WMD = 15.94 U/mL; 95% CI: 5.86, 26.02) whereas the single non-Asian study showed a non-significant positive estimate (WMD = 8.00 U/mL; 95% CI: −26.07, 42.07) (Additional file 4: Table S4).

### Glutathione Peroxidase (GSH-Px)

Four treatment arms from three reports (*n* = 142) reported GSH-Px. Compared with the control group, selenium supplementation was associated with a significant increase in GSH-Px activity (WMD = 32.37 U/L; 95% CI: 7.81, 56.93; *P* = 0.010), with very high heterogeneity (I² = 97.3%; *P* < 0.001) (Table 2, Additional file 3: Table S3).

In subgroup analyses, long interventions (> 84 days) showed increased GSH-Px activity (WMD = 47.29 U/L; 95% CI: 39.00, 55.58), whereas the single shorter intervetion (≤ 84 days) showed no statistically significant effect (WMD = 5.60 U/L; 95% CI: −1.32, 12.52). Asian studies showed significant increases (WMD = 35.23 U/L; 95% CI: 7.00, 63.45), whereas the single non-Asian study showed no statistically significant effect (WMD = 21.70 U/L; 95% CI: −2.90, 46.30) (Additional file 4: Table S4). No GSH-Px data were available from trials using exactly 200 µg/day.

#### High-sensitivity C-reactive Protein (hs-CRP)

Ten treatment arms from10 reports (*n* = 537) reported hs-CRP. Compared with the control group, selenium supplementation was associated with a significant reduction in hs-CRP (WMD = − 984.87 ng/mL; 95% CI: −1419.65, − 550.09; *P* < 0.001), with high heterogeneity (I² = 79.2%; *P* < 0.001) (Table 2, Additional file 3: Table S3).

Reductions were apparent in non-diabetic populations (WMD = − 922.32 ng/mL; 95% CI: −1402.99, − 441.64) and in shorter interventions (≤ 84 days: WMD = − 1148.57 ng/mL; 95% CI: −1739.25, − 557.90). The single longer study (> 84 days) also showed a reduction in hs-CRP (WMD = − 700.00 ng/mL; 95% CI: −944.34, − 455.66). Trials using 200 µg/day showed significant reductions (WMD = − 913.59 ng/mL; 95% CI: −1183.44, − 643.74), whereas other doses showed a negative but non-significant estimate with a wide confidence interval (WMD = − 1828.61 ng/mL; 95% CI: −5548.07, 1890.85). Asian and Iranian studies showed reductions in hs-CRP (Additional file 4: Table S4).

### Lipid outcomes

Selenium supplementation showed modest favorable changes in lipid profiles. Overall, selenium supplementation was associated with significant reductions in TC (WMD = − 4.14 mg/dL; 95% CI: −7.40, − 0.87; *P* = 0.013), TG (WMD = − 5.93 mg/dL; 95% CI: −10.18, − 1.69; *P* = 0.006), and LDL-C (WMD = − 4.27 mg/dL; 95% CI: −8.20, − 0.34; *P* = 0.033) (Additional file 3: Table S3, Additional file 6: Table S6).

Subgroup analyses suggested that reductions in TC and TG were more evident in individuals with abnormal glucose metabolism (T2DM/GDM). For example, TG was significantly reduced in the T2DM/GDM subgroup (WMD = − 18.83 mg/dL; 95% CI: −28.06, − 9.60; *P* < 0.001). Asian studies also showed a significant reduction in LDL-C (WMD = − 4.27 mg/dL; 95% CI:−8.20, − 0.34; *P* = 0.033).

Shorter interventions (≤ 84 days) were associated with greater reductions in TG (WMD = − 9.07 mg/dL; 95% CI: −13.99, − 4.14; *P* < 0.001). A dose of 200 µg/day was associated with significant reductions in TC (WMD = − 4.14 mg/dL; 95% CI: −7.62, − 0.66; *P* = 0.020) and TG (WMD = − 7.38 mg/dL; 95% CI: −11.93, − 2.83; *P* = 0.001), and a borderline reduction in LDL-C (WMD = − 4.24 mg/dL; 95% CI: −8.64, 0.17; *P* = 0.059). Heterogeneity was low-to-moderate for TC (I² = 29.2%; *P* = 0.114) and TG (I² = 26.3%; *P* = 0.132), and moderate for LDL-C (I² = 49.7%; *P* = 0.009), with generally consistent directions of effect across most subgroups.

Selenium supplementation was not associated with a significantchange in VLDL-C (WMD = − 0.55 mg/dL; 95% CI: −1.65, 0.54; *P* = 0.321; I² = 24.9%; *P* = 0.214). In contrast, HDL-C increased modestly (WMD = 2.63 mg/dL; 95% CI: 0.01, 5.26; *P* = 0.049), although with substantial heterogeneity (I² = 95.2%; *P* < 0.001) (Additional file 4: Table S4). Overall, selenium supplementation was associated with modest favorable changes in lipid parameters, including lower TC, TG, and LDL-C and slightly higher HDL-C, while showing no clear effect on VLDL-C. These patterns appeared more evident in dysglycemic populations, Asian settings, shorter interventions, and trials using 200 µg/day but subgroup findings should be interpreted cautiously.

### Anthropometric and blood pressure outcomes

Selenium supplementation showed no statistically significant effects on most anthropometric or blood pressure outcomes. Pooled analyses showed no significant effects on weight (WMD = 0.03 kg; 95% CI: −0.11, 0.16; *P* = 0.688), BMI (WMD = 0.00 kg/m²; 95% CI: −0.05, 0.05; *P* = 0.934), SBP (WMD = 1.04 mmHg; 95% CI: −0.01, 2.10; *P* = 0.071), or WC (WMD = 0.76 cm; 95% CI: −0.23, 1.75; *P* = 0.133), with no detectable heterogeneity for these outcomes (I² = 0.0% for all; all P for heterogeneity > 0.05) (Additional file 3: Table S3, Additional file 6: Table S6).

In contrast, selenium supplementation was associated with a modest reduction in DBP (WMD = − 1.56 mmHg; 95% CI: −2.30, − 0.81; *P* < 0.001), with low heterogeneity (I² = 15.1%; *P* = 0.311). Subgroup analyses by population type (T2DM/GDM vs. non-diabetic populations), region (Asia vs. non-Asia), duration, and dose showed generally consistent null findings for weight, BMI, SBP, and WC, whereas the DBP-lowering association appeared relatively stable across most strata.

### Subgroup analysis according to intervention context

Subgroup analyses by intervention context showed that the effects of selenium supplementation were generally consistent between selenium-alone trials and balanced co-intervention trials. In balanced co-intervention trials, both groups received the same active background intervention, so that the intended between-group contrast isolated the incremental effect of selenium. No significant subgroup differences were observed for most metabolic outcomes, including FPG, HbA1c, FINS, HOMA-IR, HOMA-β, QUICKI, lipid parameters, inflammatory biomarkers, and anthropometric measures. Significant subgroup differences were detected only for TAC and NO, suggesting that the background intervention context may influence selected oxidative stress-related biomarkers. These findings support cautious interpretation of TAC and NO while indicating that the main metabolic conclusions were not primarily explained by intervention context.

### Nonlinear dose-response relationships

Nonlinear dose-response analyses suggested nonlinear patterns between selenium dose and several metabolic and oxidative-stress-related biomarkers. As selenium dose increased from lower levels, serum selenium concentrations rose, with concurrent favorable trends in selected markers including FPG, FINS, HOMA-IR, and antioxidant enzyme activities.

**Fig. 6:**
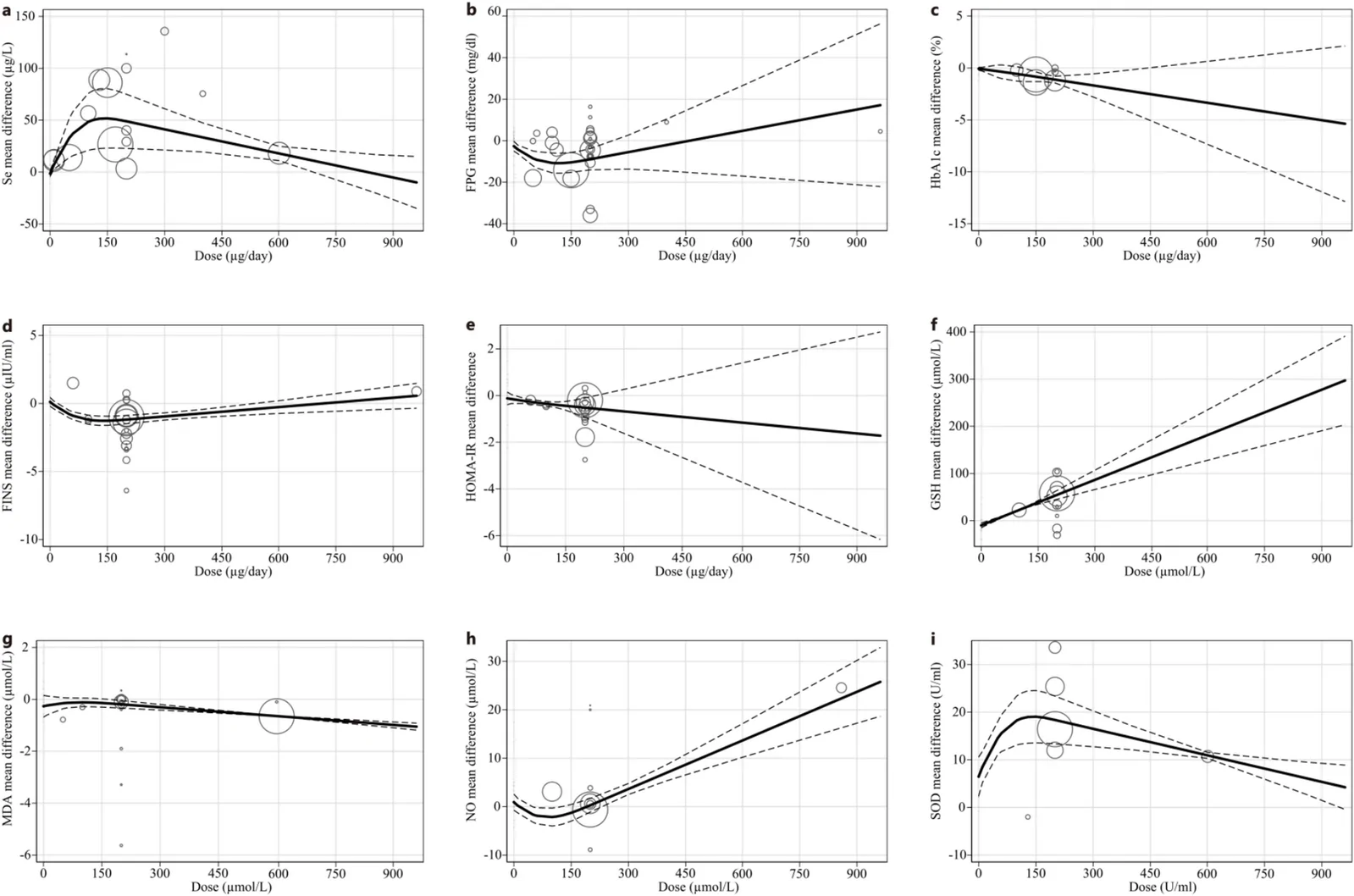
Dose-response relationships for selenium supplementation. **a **Serum selenium; (**b**) fasting plasma glucose (FPG); (**c**) HbA1c; (**d**) fasting insulin (FINS); (**e**) HOMA-IR; (**f**) glutathione (GSH); (**g**) malondialdehyde (MDA); (**h**) nitric oxide (NO); (**i**) superoxide dismutase (SOD). Dose-response curves were estimated using the nonlinear robust error meta-regression (REMR) model, based on inverse variance–weighted least-squares regression with cluster-robust error variance. Solid lines represent pooled effects; shaded bands show 95% confidence intervals

Across outcomes, the most favorable biomarker pattern appeared to occur within approximately 50–200 µg/day, particularly around 100–150 µg/day, where serum selenium increased and FPG, FINS, and hs-CRP tended to decline, alongside increases in SOD and GSH-Px (Fig. 6). Beyond approximately 200 µg/day, the magnitude of favorable changes appeared to plateau or attenuate for some outcomes: At the highest doses, up to 960 µg/day, confidence intervals were wide, reflecting limited data and greater uncertainty.

When interpreted alongside dietary reference values from the WHO, EFSA, and NIH, which generally suggest recommended adult selenium intakes of approximately 55–70 µg/day and a tolerable upper intake level of 400 µg/day [63–65]—our findings are compatible with an exploratory low-to-moderate supplementation range of approximately 100–150 µg/day. Within this range, selenium supplementation was associated with favorable biomarker changes while remaining below commonly cited upper intake thresholds. Nevertheless, because confidence intervals widened considerably at higher doses and the number of high-dose data points was limited, these nonlinear associations should be regarded as exploratory rather than definitive.

### Sensitivity analyses

Leave-one-out sensitivity analyses showed that no single study qualitatively changed the pooled effects of selenium supplementation on the main glycemic, oxidative-stress-related, inflammatory, and selenium-status outcomes (Additional file 11: Figure S2). However, heterogeneity remained substantial for several outcomes, particularly serum selenium, MDA, and GSH-Px, suggesting that the observed variability may reflect clinical and methodological differences across studies. Potential contributors included differences in baseline metabolic status, baseline selenium status, supplementation dose, intervention duration, geographic region, and selenium formulation. Thus, although the overall direction of several effects was generally preserved, the magnitude of pooled estimates should be interpreted cautiously.

For TAC, removal of two potential outlier studies, Balali et al. [50] and Tian et al. [52] changed the pooled mean difference by more then 10%, suggesting that part of the observed heterogeneity and subgroup variation may have been influenced by a small number of studies with relatively large effects. Nevertheless, sensitivity analysis using a fixed-effect model after excluding these studies did not materially alter the direction or statistical significance of the overall association.

### Publication bias and small-study effects

Visual inspection of funnel plots suggested varying degrees of asymmetry for several outcomes, whereas relatively symmetrical distributions were observed for FINS and HOMA-IR (Additional file 12: Figure S3).

Formal tests for funnel plot asymmetry were performed only for outcomes with at least 10 contributing studies. No statistical evidence of funnel plot asymmetry was detected for FINS, HOMA-IR, QUICKI, GSH, MDA, NO, or serum selenium. In contrast, Egger’s test suggested possible small-study effects for FPG (*P* = 0.028), TAC (*P* = 0.002), and hs-CRP (*P* = 0.005; Begg’s test *P* = 0.904) (Table 2, Additional file 6: Table S6).

These assessments should be interpreted cautiously. Funnel plot asymmetry may reflect publication bias, but it may also arise from between-study heterogeneity, selective reporting, methodological differences, or other small-study effects. For outcomes with fewer than 10 contributing studies, including HbA1c, HOMA-β, SOD, GSH-Px, SBP, DBP, and WC, formal tests for funnel plot asymmetry were not performed because the results would be underpowered and unreliable. Therefore, publication bias could not be reliably excluded for these outcomes.

### GRADE certainty of evidence

Using the revised GRADE assessment (Additional file 8: Table S8), the certainty of evidence varied across outcomes. The main reasons for downgrading were substantial between-study inconsistency, imprecision due to limited sample size or confidence intervals crossing the line of no effect, indirectness related to intervention context for selected oxidative-stress-related outcomes, and possible small-study effects or publication bias for FPG, TAC, and hs-CRP.

For glycemic control and insulin-related outcomes, selenium supplementation had no clear effect on FPG (WMD = − 2.15 mg/dL; 95% CI: −6.43, 2.14; very low certainty) or HbA1c (WMD = − 0.21%; 95% CI: −0.63, 0.20; very low certainty). Selenium supplementation reduced FINS (WMD = − 1.47 µIU/mL; 95% CI: −2.37, − 0.57; moderate certainty) and HOMA-IR (WMD = − 0.58; 95% CI: −0.84, − 0.31; low certainty), was associated with lower HOMA-β values (WMD = − 17.57; 95% CI: −23.91, − 11.23; low certainty), and increased QUICKI (WMD = 0.010; 95% CI: 0.006, 0.015, low certainty).

For oxidative-stress-related and inflammatory biomarkers, selenium supplementation increased GSH (WMD = 55.87; 95% CI: 31.58, 80.17; moderate certainty) and TAC (WMD = 23.76; 95% CI: 8.80, 38.72; very low certainty), SOD (WMD = 15.48 U/mL; 95% CI: 5.77, 25.19; very low certainty), GSH-Px (WMD = 32.37 U/L; 95% CI: 7.81, 56.93; very low certainty), and serum selenium (WMD = 53.56 µg/L; 95% CI: 34.33, 72.79; low certainty). Selenium supplementation decreased MDA (WMD = − 1.08; 95% CI: −1.62, − 0.54; low certainty) and hs-CRP (WMD = − 984.87 ng/mL; 95% CI: −1419.65, − 550.09; very low certainty), while no clear effect was observed for NO (WMD = 0.53 µmol/L; 95% CI: −1.32, 2.38; very low certainty).

For lipid outcomes, selenium increased HDL-C (WMD = 2.63 mg/dL; 95% CI: 0.01, 5.26; low certainty) and reduced LDL-C (WMD = − 4.27 mg/dL; 95% CI: −8.20, − 0.34; moderate certainty), TG (WMD = − 5.93 mg/dL; 95% CI: −10.18, − 1.69; high certainty), and TC (WMD = − 4.14 mg/dL; 95% CI: −7.40, − 0.87; high certainty). No clear effect was observed for VLDL-C (WMD = − 0.55 mg/dL; 95% CI: −1.65, 0.54; moderate certainty).

For anthropometric and blood pressure outcomes, selenium supplementation had no clear effect on BMI (WMD = 0.00 kg/m²; 95% CI: −0.05, 0.05; high certainty), weight (WMD = 0.03 kg; 95% CI: −0.11, 0.16; moderate certainty), SBP (WMD = 1.04 mmHg; 95% CI: −0.01, 2.10; moderate certainty), or WC (WMD = 0.76 cm; 95% CI: −0.23, 1.75; low certainty). Selenium supplementation modestly reduced DBP (WMD = − 1.56 mmHg; 95% CI: −2.30, − 0.81; moderate certainty).

## Discussion

This systematic review and meta-analysis comprehensively evaluated the effects of selenium supplementation on glycemic indices, oxidative-stress-related and inflammatory biomarkers, lipid parameters, and anthropometric or hemodynamic indicators in adults. In the primary between-group analyses, selenium supplementation showed no clear overall effect on FPG or HbA1c, but was associated with reductions in FINS and HOMA-IR, and an increase in QUICKI. These findings suggest modest improvements in insulin-sensitivity markers rather than definitive improvement in overall glycemic control. Favorable changes were also observed in selected oxidative-stress-related, inflammatory, and lipid biomarkers; however, several outcomes showed substantial heterogeneity or low-to-very-low certainty of evidence. Therefore, these findings should be interpreted as biomarker-level changes across heterogeneous trial settings rather than direct evidence of long-term clinical benefit.

In the primary between-group comparisons, selenium supplementation was not associated with significant reductions in FPG or HbA1c in the overall analysis, which is consistent with several earlier randomized trials reporting neutral effects on glycemic indices in generally normoglycemic populations [66]. This pattern may partly reflect a “ceiling effect”, whereby individuals with relatively normal baseline glycemic control have limited room for further improvement [66]. By contrast, subgroup analyses suggested that selenium supplementation may have more apparent effects in selected high-risk populations, particularly individuals with pre-existing disturbances in glucose metabolism such as T2DM or GDM. To avoid overinterpreting time-dependent changes or regression to the mean, within-group pre–post analyses were de-emphasized and moved to the Supplementary Material (Table S3 and Table S5). These exploratory analyses should not be treated as between-group randomized RCT-level evidence and should not replace the primary between-group comparisons.

Our findings should be interpreted in the context of previous evidence. Prior meta-analyses have reported inconsistent effects of selenium supplementation on glycemic control. For example, Mahdavi Gorabi et al. reported no significant overall improvement in glycemic indices after selenium supplementation, which is broadly consistent with our finding that the overall between-group effects on FPG and HbA1c were not statistically significant. However, our analysis further suggests that selenium supplementation may be associated with modest improvements in insulin-sensitivity markers, including FINS, HOMA-IR, and QUICKI, as well as favorable changes in selected oxidative-stress-related, inflammatory, and lipid biomarkers. These additional findings may reflect the larger number of randomized trials included in the present review, broader outcome coverage, updated evidence, and the use of subgroup, sensitivity, GRADE, and exploratory dose-response analyses.

Our findings should also be balanced against observational evidence and long-term high-dose trials that have raised concerns about potential adverse glycemic effects of higher selenium exposure. Observational studies, including the study by Ma et al., have reported associations between higher selenium exposure and increased diabetes risk, whereas long-term high-dose trials such as the NPC trial and the Algotar trial, have raised concerns about limited metabolic benefit or possible harm with prolonged high-dose supplementation. These findings do not necessarily contradict our results. Short-to-medium-term randomized supplementation trials, particularly those conducted in populations with marginal or low selenium status may capture biomarker-level improvements, whereas observational studies and long-term high-dose trials may reflect different exposure ranges, baseline selenium status, duration of exposure, population characteristics, and the potential risks of excessive selenium intake.

Several factors may explain discrepancies across studies. First, selenium status varies substantially across geographic regions, and populations with low or marginal selenium exposure may respond differently from selenium-replete populations. Second, supplementation dose and intervention duration varied widely across trials, and the relationship between selenium exposure and metabolic outcomes may be nonlinear. Third, many outcomes were surrogate biomarkers rather than clinical endpoints, and several pooled estimates showed substantial heterogeneity or low certainty of evidence. Therefore, the present findings should be viewed as an updated synthesis suggesting potential biomarker-level benefits in selected populations, rather than as definitive evidence supporting routine selenium supplementation for all adults.

Selenium supplementation was associated with favorable changes in several oxidative-stress-related biomarkers. Mechanistically, selenium is incorporated as selenocysteine into key selenoproteins, including glutathione peroxidases (GSH-Px) and thioredoxin reductases (TrxR) [7], which contribute to antioxidant defense, redox regulation, and protection against oxidative stress [67]. The observed reduction in MDA is consistent with reduced lipid peroxidation. Given that oxidative stress is closely linked to obesity, insulin resistance, and T2DM [68], these oxidative-stress-related biomarker changes are biologically plausible, although their clinical implications require further confirmation.

Although TAC generally increased following selenium supplementation, the exceptionally large point estimate observed in the T2DM/GDM subgroup should be interpreted cautiously because it was derived from a small number of studies and was accompanied by substantial uncertainty, as reflected by the very wide confidence interval. Therefore, this subgroup finding should be considered exploratory rather than definitive.

Improved redox status may have implications for insulin signaling. Excessive reactive oxygen species (ROS) can activate stress kinases such as JNK and IKKβ [69], which may impair insulin signaling by promoting serine phosphorylation of insulin receptor substrate (IRS), thereby interfering with downstream PI3K/Akt activation and glucose uptake [70]. In this meta-analysis, selenium supplementation significantly reduced HOMA-IR and FINS, suggesting a modest improvement in insulin sensitivity markers. A plausible mechanism is that increased activity of GSH-Px and TrxR may reduces ROS burden, dampen stress kinase activation, and help preserve insulin signaling through IRS-PI3K/Akt pathways [71]. Although most mechanistic data derive from animal and cellular models, our pooled clinical findings are consistent with this oxidative stress–insulin signaling pathway.

The enhancement of antioxidant defenses may also be linked to changes in lipid metabolism. Oxidative stress promotes lipid peroxidation, alters hepatic lipid handling, and impairs HDL-C function, thereby contributing to triglyceride accumulation and atherogenic dyslipidemia [72, 73]. In our analysis, selenium supplementation modestly increased HDL-C and decreased TG and LDL-C, with these changes appearing more evident in populations with abnormal glucose metabolism, such as T2DM or GDM. These findings are consistent with experimental evidence that selenoproteins may modulate hepatic lipogenesis and protect lipoproteins from oxidative modification [74]. In addition, selenium has been reported to influence AMPK signaling, a central hub for energy metabolism and oxidative stress. AMPK activation can suppress de novo lipogenesis and promote fatty acid oxidation [75], providing a plausible mechanistic framework for the observed lipid changes. The reduction in hs-CRP further supports a potential anti-inflammatory effect of selenium. Since chronic low-grade inflammation is another key driver of insulin resistance and atherosclerosis, selenium may influence glucose–lipid homeostasis through combined antioxidant and anti-inflammatory mechanisms.

The nonlinear dose-response analyses extend previous reviews by exploring whether biomarker changes vary across selenium doses. Dose-response modeling suggested that the most favorable pattern of biomarker changes was generally observed within a low-to-moderate dose range, approximately 100–150 µg/day. However, the strength of dose-response evidence varied across outcomes. Curves based on broader dose coverage and a larger number of contributing studies were more informative, whereas estimates supported by fewer studies or fewer distinct dose levels should be regarded as exploratory. In addition, the higher-dose range was supported by relatively few observations, resulting in greater uncertainty. Therefore, the apparent favorable low-to-moderate dose range should be interpreted as a hypothesis-generating dose-response pattern rather than as a definitive clinical dosing recommendation.

This interpretation should also be considered alongside long-term high-dose selenium trials that provide important contextual evidence for safety but were not the main drivers of the short-to-medium-term biomarker findings in the present meta-analysis. In the Nutritional Prevention of Cancer (NPC) trial, Stranges et al. [30] reported an increased incidence of type 2 diabetes among participants receiving selenium supplementation during extended follow-up, whereas Algotar et al. [24] found no clear glycemic benefit from long-term supplementation at doses as high as 400 µg/day. Although these studies differed from most trials included in our quantitative biomarker synthesis in population characteristics, supplementation dose, intervention duration, and outcome reporting, they provide important contextual safety evidence. Taken together, the available evidence suggests that chronic exposure to relatively high selenium doses may not confer additional metabolic advantages and may increase glycemic risk in some individuals. Therefore, the apparent 100–150 µg/day range should be regarded as an exploratory low-to-moderate supplementation range rather than as a recommendation for high-dose or long-term selenium use.

The observed effects also appeared to vary by intervention duration. Shorter supplementation (≤ 84 days) was associated with favorable changes in several intermediate endpoints, including FINS, HOMA-IR, GSH, TAC, and hs-CRP. Subgroup analysis showed that HbA1c was reduced only in shorter-term trials (≤ 84 days; WMD = − 0.12%; *P* = 0.032), whereas the reduction in longer-term trials did not reach statistical significance (> 84 days; WMD = − 0.23%, *P* = 0.404), likely because of the limited number of studies and wider confidence intervals in this stratum. Because most included trials were of short-to-medium duration and data beyond several months were sparse, inferences about long-term efficacy and safety remain limited.

Substantial between-study heterogeneity was a major challenge. This heterogeneity may reflect differences in baseline selenium status, habitual diet, genetic background, disease status, selenium formulation, supplementation dose, intervention duration, geographic region, assay methods, and intervention context. Subgroup analyses and leave-one-out sensitivity analyses suggested several potential contributors, but these factors did not fully explain the heterogeneity observed for several outcomes. Therefore, pooled estimates for highly heterogeneous outcomes, particularly serum selenium, MDA, GSH-Px, SOD, HDL-C, FPG, and HbA1c, should be interpreted as average effects across diverse clinical and methodological settings rather than as precise common treatment effects.

Particularly high heterogeneity was observed for several selenium-status and oxidative-stress-related outcomes, including serum selenium (I² = 98.9%), MDA (I² = 96.4%), and GSH-Px (I² = 97.3%). We retained these pooled analyses because the outcomes were prespecified, random-effects models were used, the direction of effect was generally consistent, and sensitivity analyses did not identify any single study that fully accounted for the observed associations. Nevertheless, the magnitude of these pooled WMDs should be interpreted cautiously, and greater emphasis should be placed on the direction and consistency of the associations rather than on exact point estimates.

The geographic distribution of the available evidence also limits generalizability. Approximately half of the included trials were conducted in Iran, with most remaining studies originating from China and other Asian regions. These populations may have relatively low or marginal selenium exposure compared with selenium-replete regions. Consequently, the observed metabolic and oxidative-stress-related biomarker changes may partly reflect correction of suboptimal selenium status rather than universal benefits of supplementation. Future trials should more systematically characterize baseline selenium status, dietary patterns, and relevant genetic factors, and should prespecify subgroup analyses based on these factors to better capture effect modification.

Publication bias and small-study effects should also be considered. Possible small-study effects were suggested for FPG, TAC, and hs-CRP, whereas formal tests were not performed for outcomes with fewer than 10 studies. Non-significant funnel plot tests should not be interpreted as evidence that publication bias is absent, particularly when substantial heterogeneity is present. Therefore, some pooled effects, especially those supported mainly by small trials, may have been overestimated.

The potential influence of co-interventions should be interpreted in light of the eligibility criteria. We did not include trials in which selenium plus another active intervention was compared with placebo or no treatment alone. When active co-interventions were present, the same background intervention was required in both groups, so that the intended between-group contrast isolated the incremental effect of selenium. Therefore, these trials still estimated the incremental effect of selenium in a balanced co-intervention context. Subgroup analyses comparing selenium-alone trials with balanced co-intervention trials showed generally consistent findings for most metabolic outcomes, although TAC and NO differed by intervention context. These results suggest that the main insulin-sensitivity and lipid findings were not driven by unbalanced co-interventions, whereas selected oxidative-stress-related biomarkers should be interpreted more cautiously.

Several limitations should be acknowledged. First, considerable heterogeneity remained unexplained despite prespecified subgroup analyses and leave-one-out sensitivity analyses, limiting the precision and generalizability of pooled WMDs for several outcomes. Subgroup findings should therefore be considered exploratory and hypothesis-generating rather than confirmatory evidence of differential treatment effects. Second, the number of available trials was limited for several key outcomes, including HOMA-β, TAC, GSH-Px, hs-CRP, and SBP, leading to imprecise estimates and lower GRADE certainty. Third, most interventions were of short-to-medium duration, and data on longer-term outcomes and safety, particularly with repeated or high-dose supplementation, remain limited. Fourth, incomplete reporting in the original trials limited our ability to account for physical activity, detailed dietary intake, concomitant pharmacotherapy, and baseline selenium exposure. Fifth, given the large number of outcomes and subgroup analyses assessed, some statistically significant findings, especially for secondary endpoints, may have arisen by chance because of multiple comparisons.

From a clinical and public health perspective, selenium supplementation should be viewed as a potential adjunctive and individualized strategy rather than as a stand-alone or routine therapy. In adults with insulin resistance or diabetes, particularly in regions or populations with marginal or low baseline selenium status, short-term supplementation within a low-to-moderate dose range may be associated with favorable changes in insulin-sensitivity markers, oxidative-stress-related biomarkers, and selected lipid parameters. However, the current evidence remains biomarker-based and is insufficient to support selenium supplementation as part of standard treatment algorithms for T2DM, GDM, or metabolic syndrome. Decisions regarding supplementation should consider regional selenium exposure, dietary intake, baseline selenium status, comorbidities, and potential risks associated with chronic high-dose use. Future well-designed RCTs should explicitly stratify or adjust for baseline selenium status and evaluate clinically meaningful endpoints such as incident diabetes, cardiovascular events, and long-term safety.

Consistent with the revised GRADE assessment, these findings should be interpreted cautiously because the certainty of evidence was frequently downgraded for substantial heterogeneity, imprecision, potential publication bias, and, for selected outcomes, indirectness related to intervention context.

## Conclusion

In this systematic review and meta-analysis of randomized controlled trials, selenium supplementation was associated with modest improvements in insulin sensitivity markers and selected oxidative-stress-related, inflammatory, and lipid biomarkers in adults. Selenium supplementation was associated with reductions in fasting insulin and HOMA-IR and an increase in QUICKI, whereas no clear overall effects were observed for FPG or HbA1c. Favorable changes were also observed in several oxidative-stress-related and lipid biomarkers, but these findings should be interpreted cautiously because heterogeneity was substantial for several outcomes and the certainty of evidence varied across endpoints.

The available evidence was geographically concentrated, with approximately half of the included trials conducted in Iran and many of the remaining studies conducted in China or other Asian regions. Therefore, these findings may be most directly applicable to adults with impaired metabolic status and marginal or low baseline selenium status, rather than to all adults or selenium-replete populations. Dose-response and subgroup analyses suggest that a low-to-moderate supplementation range may favorable, but this funding should be regarded as exploratory rather than as a definitive clinical dosing recommendation.

Overall, selenium supplementation may have a potential adjunctive role in selected populations, but the evidence remains primarily biomarker-based and does not support routine selenium supplementation for all adults. Larger, high-quality, long-term randomized controlled trials across diverse selenium-exposure settings are needed to clarify baseline-status-specific effects, safe dose ranges, long-term safety, and effects on clinically meaningful outcomes.

## Supplementary Information

Additional file 1: Additional file 1: Table S1 Full electronic search strategies for all databases. Additional file 2: Table S2 Excluded articles and reasons for exclusion. Additional file 3: Table S3 Comparison of glycemic, antioxidant, lipid, and anthropometric indicators. Additional file 4: Table S4 Subgroup analyses for metabolic and antioxidant outcomes. Additional file 5: Table S5 Subgroup analyses by pre- and post-intervention status. Additional file 6: Table S6 Meta-analysis results for lipid and anthropometric outcomes. Additional file 7: Table S7 Risk of bias assessment of the included randomized controlled trials using the Cochrane risk-of-bias tool. Additional file 8: Table S8 GRADE summary of findings and certainty of evidence. Additional file 9: Table S9 Subgroup analysis by intervention context: selenium-alone trials versus balanced co-intervention trials. Additional file 10: Figure S1 Quality Evaluation plot. Additional file 11: Figure S2 Leave-one-out sensitivity analyses for the primary outcomes. Additional file 12: Figure S3 Funnel plots assessing publication bias.

## Data Availability

We conducted a systematic search of nine electronic databases—PubMed, Embase, Cochrane Central Register of Controlled Trials (CENTRAL), Web of Science, CINAHL (EBSCO), CNKI, WanFang Data, VIP, and SinoMed—from inception to 12 August 2025. The following terms and their combinations were used in titles and abstracts: “selenium,” “selenium supplement,” “blood glucose index,” “insulin resistance,” “oxidative stress,” and “glutathione peroxidase.” Detailed search strategies for each database are provided in Additional file 1: Table S1.To maximize trial capture and enhance the comprehensiveness of our review, we also manually screened the reference lists of relevant articles. The review protocol was prospectively registered in PROSPERO (Registration number: CRD420251124852).

## Abbreviations

BMI: Body Mass Index
CI: Confidence Interval
CINAHL: Cumulative Index To Nursing And Allied Health Literature
CNKI: China National Knowledge Infrastructure
CRP: C-Reactive Protein
CVD: Cardiovascular Disease
DBP: Diastolic Blood Pressure
EFSA: European Food Safety Authority
FPG: Fasting Plasma Glucose
FINS: Fasting Insulin
GDM: Gestational Diabetes Mellitus
GRADE: Grading Of Recommendations, Assessment, Development, And Evaluations
GSH: Glutathione
GSH-Px: Glutathione Peroxidase
HbA1c: Glycated Hemoglobin
HDL-C: High-Density Lipoprotein
HOMA-β: Homeostasis Model Assessment Of β-Cell Function
HOMA-IR: Homeostasis Model Assessment Of Insulin Resistance
hs-CRP: High-Sensitivity C-reactive protein
LDL-C: Low-Density Lipoprotein
MDA: Malondialdehyde
MetS: Metabolic Syndrome
NIH: National Institutes Of Health
NO: Nitric Oxide
OIS: Optimal Information Size
PRISMA: Preferred Reporting Items For Systematic Reviews And Meta-Analyses
QUICKI: Quantitative Insulin Sensitivity Check Index
RCT(s): Randomized Controlled Trial(S)
REMR: Robust Error Meta-Regression
SBP: Systolic Blood Pressure
SD: Standard Deviation
Se: Selenium
SE: Standard Error
SOD: Superoxide Dismutase
T2DM: Type 2 Diabetes Mellitus
TAC: Total Antioxidant Capacity
TC: Total Cholesterol
TG: Triglycerides
UL: Tolerable Upper Intake Level
VLDL-C: Very-Low-Density Lipoprotein
WC: Waist Circumference
WHO: World Health Organization
WMD: Weighted Mean Difference
