# Supplementary material for: Effects of selenium supplementation on glycemic indices and oxidative-stress biomarkers in adults: a systematic review, meta-analysis, and dose-response analysis of randomized controlled trials
Source: Nutr J. 2026 Aug 1;25:118. doi: 10.1186/s12937-026-01376-w (PMC13721686; doi:10.1186/s12937-026-01376-w)
Supplement: Supplementary file 1 [file 12937_2026_1376_MOESM1_ESM.docx]

**Additional files**

**Additional file 1: Table S1** Full electronic search strategies for all databases.

**Additional file 2: Table S2** Excluded articles and reasons for exclusion.

**Additional file 3: Table S3** Comparison of glycemic, antioxidant, lipid, and anthropometric indicators.

**Additional file 4: Table S4** Subgroup analyses for metabolic and antioxidant outcomes.

**Additional file 5: Table S5** Subgroup analyses by pre- and post-intervention status.

**Additional file 6: Table S6** Meta-analysis results for lipid and anthropometric outcomes.

**Additional file 7: Table S7** Risk of bias assessment of the included randomized controlled trials using the Cochrane risk-of-bias tool.

**Additional file 8: Table S8** GRADE summary of findings and certainty of evidence.

**Additional file 9: Table S9** Subgroup analysis by intervention context: selenium-alone trials versus balanced co-intervention trials.

**Additional file 10: Figure S1** Quality Evaluation plot.

**Additional file 11: Figure S2** Leave-one-out sensitivity analyses for the primary outcomes.

**Additional file 12: Figure S3** Funnel plots assessing publication bias.

**Additional file 1: Table S1** Full electronic search strategies for all databases.

| Online databases | Search strategy |
| --- | --- |
| PubMed | ((""Selenium""[Mesh] OR seleni*[tiab] OR selenomethionine[tiab] OR ""selenium yeast""[tiab] OR ""selenium-enriched yeast""[tiab] OR ""selenized yeast""[tiab] OR selenite[tiab] OR ""sodium selenite""[tiab] OR selenate[tiab] OR ""selenium supplementation""[tiab] OR ""selenium supplement*""[tiab] OR ""dietary selenium""[tiab] OR ""selenium intake""[tiab] OR ""selenium-fortified""[tiab] OR ""selenium-enriched""[tiab]) AND (( ""Glycated Hemoglobin""[Mesh] OR ""glycosylated hemoglobin""[tiab] OR ""glycated hemoglobin""[tiab] OR HbA1c[tiab] OR HBA1c[tiab] OR ""fasting plasma glucose""[tiab] OR ""fasting glucose""[tiab] OR FPG[tiab] OR glucose[tiab] OR glycemic*[tiab] OR glycaemic*[tiab] OR ""postprandial glucose""[tiab] OR ""Insulin Resistance""[Mesh] OR ""insulin resistance""[tiab] OR ""homeostasis model assessment""[tiab] OR HOMA-IR[tiab] OR ""HOMA IR""[tiab] OR ""insulin sensitivity""[tiab] OR QUICKI[tiab] OR HOMA-β[tiab] OR ""beta-cell function""[tiab] OR insulin[tiab]) OR ( ""Oxidative Stress""[Mesh] OR oxidative stress[tiab] OR ""Antioxidants""[Mesh] OR antioxidant*[tiab] OR ""Reactive Oxygen Species""[Mesh] OR ""Lipid Peroxidation""[Mesh] OR ""lipid peroxidation""[tiab] OR ""Malondialdehyde""[Mesh] OR malondialdehyde[tiab] OR MDA[tiab] OR TBARS[tiab] OR ""Nitric Oxide""[Mesh] OR ""nitric oxide""[tiab] OR ""Isoprostanes""[Mesh] OR isoprostane*[tiab] OR ""Superoxide Dismutase""[Mesh] OR ""superoxide dismutase""[tiab] OR SOD[tiab] OR ""Catalase""[Mesh] OR catalase[tiab] OR CAT[tiab] OR ""Glutathione""[Mesh] OR glutathione[tiab] OR GSH[tiab] OR GSSG[tiab] OR ""GSH/GSSG""[tiab] OR ""total antioxidant capacity""[tiab] OR TAC[tiab] OR ""ferric reducing antioxidant power""[tiab] OR FRAP[tiab] OR ""protein carbonyl*""[tiab] OR ""advanced oxidation protein products""[tiab] OR AOPP[tiab] OR ""Oxidized Low-Density Lipoprotein""[tiab] OR ""oxidized LDL""[tiab] OR oxLDL[tiab] OR ""oxidised LDL""[tiab] OR ""8-hydroxy-2'-deoxyguanosine""[tiab] OR ""8-hydroxy-2-deoxyguanosine""[tiab] OR 8-OHdG[tiab] OR ""Glutathione Peroxidase""[Mesh] OR ""glutathione peroxidase""[tiab] OR GPx[tiab] OR ""GSH-Px""[tiab])) AND (randomized controlled trial[pt] OR controlled clinical trial[pt] OR randomized[tiab] OR randomised[tiab] OR randomly[tiab] OR placebo[tiab] OR ""placebo-controlled""[tiab] OR trial[ti] OR ""double blind""[tiab] OR ""single blind""[tiab] OR ""triple blind""[tiab])) NOT (""animals""[Mesh] NOT ""humans""[Mesh]) NOT (animal*[tiab] OR nonhuman*[tiab] OR murine[tiab] OR mouse[tiab] OR mice[tiab] OR rodent*[tiab] OR rat[tiab] OR rats[tiab] OR porcine[tiab] OR swine[tiab] OR pigs[tiab] OR piglet*[tiab] OR bovine[tiab] OR cattle[tiab] OR ovine[tiab] OR sheep[tiab] OR caprine[tiab] OR goat*[tiab] OR equine[tiab] OR horse[tiab] OR horses[tiab] OR canine[tiab] OR dogs[tiab] OR dog[tiab] OR feline[tiab] OR cats[tiab] OR rabbit*[tiab] OR hamster*[tiab] OR ""guinea pig*""[tiab] OR zebrafish[tiab] OR fish[tiab] OR fishes[tiab] OR piscine[tiab] OR avian[tiab] OR poultry[tiab] OR chicken*[tiab] OR broiler*[tiab] OR quail*[tiab] OR duck*[tiab] OR hens[tiab] OR hen[tiab] OR carp[tiab] OR tilapia[tiab] OR veterinary[tiab]) |
| EMbase | ('selenium'/exp OR selenium:ti,ab,kw OR seleni*:ti,ab,kw OR selenomethionine:ti,ab,kw OR 'selenium yeast':ti,ab,kw OR 'selenium-enriched yeast':ti,ab,kw OR 'selenized yeast':ti,ab,kw OR selenite:ti,ab,kw OR 'sodium selenite':ti,ab,kw OR selenate:ti,ab,kw OR 'selenium supplementation':ti,ab,kw OR 'selenium supplement*':ti,ab,kw OR 'dietary selenium':ti,ab,kw OR 'selenium intake':ti,ab,kw OR 'selenium-fortified':ti,ab,kw OR 'selenium-enriched':ti,ab,kw) AND (('hemoglobin a1c'/exp OR 'insulin resistance'/exp OR 'glycosylated hemoglobin':ti,ab,kw OR 'glycated hemoglobin':ti,ab,kw OR hba1c:ti,ab,kw OR 'fasting plasma glucose':ti,ab,kw OR 'fasting glucose':ti,ab,kw OR fpg:ti,ab,kw OR glucose:ti,ab,kw OR glycemic*:ti,ab,kw OR glycaemic*:ti,ab,kw OR 'postprandial glucose':ti,ab,kw OR 'homeostasis model assessment':ti,ab,kw OR 'homa ir':ti,ab,kw OR 'insulin sensitivity':ti,ab,kw OR quicki:ti,ab,kw OR 'homa b':ti,ab,kw OR 'beta-cell function':ti,ab,kw OR insulin:ti,ab,kw) OR ('oxidative stress'/exp OR 'antioxidant'/exp OR 'reactive oxygen species'/exp OR 'lipid peroxidation'/exp OR 'malondialdehyde'/exp OR 'nitric oxide'/exp OR 'isoprostane'/exp OR 'superoxide dismutase'/exp OR 'catalase'/exp OR 'glutathione'/exp OR 'oxidized low density lipoprotein'/exp OR '8 hydroxydeoxyguanosine'/exp OR 'lipid peroxidation':ti,ab,kw OR malondialdehyde:ti,ab,kw OR mda:ti,ab,kw OR 't bars':ti,ab,kw OR tbars:ti,ab,kw OR 'nitric oxide':ti,ab,kw OR isoprostane*:ti,ab,kw OR 'superoxide dismutase':ti,ab,kw OR sod:ti,ab,kw OR catalase:ti,ab,kw OR cat:ti,ab,kw OR glutathione:ti,ab,kw OR gsh:ti,ab,kw OR gssg:ti,ab,kw OR 'gsh/gssg':ti,ab,kw OR 'total antioxidant capacity':ti,ab,kw OR tac:ti,ab,kw OR 'ferric reducing antioxidant power':ti,ab,kw OR frap:ti,ab,kw OR 'protein carbonyl*':ti,ab,kw OR 'advanced oxidation protein products':ti,ab,kw OR aopp:ti,ab,kw OR 'oxidized ldl':ti,ab,kw OR oxldl:ti,ab,kw OR 'oxidised ldl':ti,ab,kw OR '8-hydroxy-2-deoxyguanosine':ti,ab,kw OR '8 ohdg':ti,ab,kw OR 'glutathione peroxidase':ti,ab,kw OR gpx:ti,ab,kw OR 'gsh-px':ti,ab,kw)) AND ('randomized controlled trial'/exp OR 'randomization'/exp OR 'double blind procedure'/exp OR 'single blind procedure'/exp OR 'placebo'/exp OR random*:ti,ab,kw OR placebo:ti,ab,kw OR 'placebo-controlled':ti,ab,kw OR 'double blind':ti,ab,kw OR 'single blind':ti,ab,kw OR 'triple blind':ti,ab,kw OR trial:ti) AND [humans]/lim NOT [animals]/lim NOT (animal*:ti,ab,kw OR nonhuman*:ti,ab,kw OR murine:ti,ab,kw OR mouse:ti,ab,kw OR mice:ti,ab,kw OR rodent*:ti,ab,kw OR rat:ti,ab,kw OR rats:ti,ab,kw OR porcine:ti,ab,kw OR swine:ti,ab,kw OR pigs:ti,ab,kw OR piglet*:ti,ab,kw OR bovine:ti,ab,kw OR cattle:ti,ab,kw OR ovine:ti,ab,kw OR sheep:ti,ab,kw OR caprine:ti,ab,kw OR goat*:ti,ab,kw OR equine:ti,ab,kw OR horse:ti,ab,kw OR horses:ti,ab,kw OR canine:ti,ab,kw OR dogs:ti,ab,kw OR dog:ti,ab,kw OR feline:ti,ab,kw OR cats:ti,ab,kw OR rabbit*:ti,ab,kw OR hamster*:ti,ab,kw OR 'guinea pig*':ti,ab,kw OR zebrafish:ti,ab,kw OR fish:ti,ab,kw OR fishes:ti,ab,kw OR piscine:ti,ab,kw OR avian:ti,ab,kw OR poultry:ti,ab,kw OR chicken*:ti,ab,kw OR broiler*:ti,ab,kw OR quail*:ti,ab,kw OR duck*:ti,ab,kw OR hens:ti,ab,kw OR hen:ti,ab,kw OR carp:ti,ab,kw OR tilapia:ti,ab,kw OR veterinary:ti,ab,kw) |
| Cochrane Central Register of Controlled Trials (CENTRAL) | Title Abstract Keyword=((Selenium OR Seleni* OR selenomethionine OR ""selenium yeast"" OR ""selenium-enriched yeast"" OR ""selenized yeast"" OR selenite OR “sodium selenite” OR selenate OR “selenium supplementation” OR “selenium supplement” OR “dietary selenium” OR “selenium intake” OR “selenium-fortified” OR “selenium-enriched”)) AND Title Abstract Keyword=((""glycosylated hemoglobin"" OR ""glycated hemoglobin"" OR HbA1c OR HBA1c OR ""fasting plasma glucose"" OR ""fasting glucose"" OR glycemic* OR glycaemic* OR ""postprandial glucose"" OR ""insulin resistance"" OR ""homeostasis model assessment"" OR HOMA-IR OR ""HOMA IR"" OR ""insulin sensitivity"" OR QUICKI OR HOMA-β OR ""beta-cell function"" OR insulin OR ""oxidative stress"" OR antioxidant* OR ""reactive oxygen species"" OR ""lipid peroxidation"" OR malondialdehyde OR MDA OR TBARS OR ""nitric oxide"" OR isoprostane* OR ""superoxide dismutase"" OR SOD OR catalase OR CAT OR glutathione OR GSH OR GSSG OR ""GSH/GSSG"" OR ""total antioxidant capacity"" OR TAC OR ""ferric reducing antioxidant power"" OR FRAP OR ""protein carbonyl"" OR ""advanced oxidation protein products"" OR AOPP OR ""oxidized LDL"" OR oxLDL OR ""oxidised LDL"" OR ""8-hydroxy-2-deoxyguanosine"" OR ""8-hydroxy-2-deoxyguanosine"" OR ""glutathione peroxidase"" OR GPx OR ""GSH-Px"")) AND Title Abstract Keyword=(random* OR placebo OR ""placebo-controlled"" OR ""double blind"" OR ""single blind"" OR ""triple blind"") NOT Title Abstract Keyword=(animal* OR nonhuman* OR murine OR mouse OR mice OR rodent* OR rat OR rats OR porcine OR swine OR pigs OR piglet* OR bovine OR cattle OR ovine OR sheep OR caprine OR goat* OR equine OR horse OR horses OR canine OR dogs OR dog OR feline OR cats OR rabbit* OR hamster* OR ""guinea pigs"" OR zebrafish OR fish OR fishes OR piscine OR avian OR poultry OR chicken* OR broiler* OR quail* OR duck* OR hens OR hen OR carp OR tilapia OR veterinary) |
| Web of Science | TS=((""Selenium"" OR seleni* OR selenomethionine OR ""selenium yeast"" OR ""selenium-enriched yeast"" OR ""selenized yeast"" OR selenite OR ""sodium selenite"" OR selenate OR ""selenium supplementation"" OR ""selenium supplement*"" OR ""dietary selenium"" OR ""selenium intake"" OR ""selenium-fortified"" OR ""selenium-enriched"")) AND TS=((""glycosylated hemoglobin"" OR ""glycated hemoglobin"" OR HbA1c OR HBA1c OR ""fasting plasma glucose"" OR ""fasting glucose"" OR FPG OR glucose OR glycemic* OR glycaemic* OR ""postprandial glucose"" OR ""insulin resistance"" OR ""homeostasis model assessment"" OR HOMA-IR OR ""HOMA IR"" OR ""insulin sensitivity"" OR QUICKI OR HOMA-β OR ""beta-cell function"" OR insulin OR ""oxidative stress"" OR antioxidant* OR ""reactive oxygen species"" OR ""lipid peroxidation"" OR malondialdehyde OR MDA OR TBARS OR ""nitric oxide"" OR isoprostane* OR ""superoxide dismutase"" OR SOD OR catalase OR CAT OR glutathione OR GSH OR GSSG OR ""GSH/GSSG"" OR ""total antioxidant capacity"" OR TAC OR ""ferric reducing antioxidant power"" OR FRAP OR ""protein carbonyl*"" OR ""advanced oxidation protein products"" OR AOPP OR ""oxidized LDL"" OR oxLDL OR ""oxidised LDL"" OR ""8-hydroxy-2'-deoxyguanosine"" OR ""8-hydroxy-2-deoxyguanosine"" OR 8-OHdG OR ""glutathione peroxidase"" OR GPx OR ""GSH-Px"")) AND TS=(random* OR placebo OR ""placebo-controlled"" OR ""double blind"" OR ""single blind"" OR ""triple blind"" OR trial) NOT TS=(animal* OR nonhuman* OR murine OR mouse OR mice OR rodent* OR rat OR rats OR porcine OR swine OR pigs OR piglet* OR bovine OR cattle OR ovine OR sheep OR caprine OR goat* OR equine OR horse OR horses OR canine OR dogs OR dog OR feline OR cats OR rabbit* OR hamster* OR ""guinea pig*"" OR zebrafish OR fish OR fishes OR piscine OR avian OR poultry OR chicken* OR broiler* OR quail* OR duck* OR hens OR hen OR carp OR tilapia OR veterinary) |
| CINAHL (EBSCO) | SU "Selenium" OR seleni* OR selenomethionine OR "selenium yeast" OR "selenium-enriched yeast" OR "selenized yeast" OR selenite OR "sodium selenite" OR selenate OR "selenium supplementation" OR "selenium supplement*" OR "dietary selenium" OR "selenium intake" OR "selenium-fortified" OR "selenium-enriched" AND SU "glycosylated hemoglobin" OR "glycated hemoglobin" OR HbA1c OR HBA1c OR "fasting plasma glucose" OR "fasting glucose" OR FPG OR glucose OR glycemic* OR glycaemic* OR "postprandial glucose" OR "insulin resistance" OR "homeostasis model assessment" OR HOMA-IR OR "HOMA IR" OR "insulin sensitivity" OR QUICKI OR HOMA-β OR "beta-cell function" OR insulin OR "oxidative stress" OR antioxidant* OR "reactive oxygen species" OR "lipid peroxidation" OR malondialdehyde OR MDA OR TBARS OR "nitric oxide" OR isoprostane* OR "superoxide dismutase" OR SOD OR catalase OR CAT OR glutathione OR GSH OR GSSG OR "GSH/GSSG" OR "total antioxidant capacity" OR TAC OR "ferric reducing antioxidant power" OR FRAP OR "protein carbonyl*" OR "advanced oxidation protein products" OR AOPP OR "oxidized LDL" OR oxLDL OR "oxidised LDL" OR "8-hydroxy-2'-deoxyguanosine" OR "8-hydroxy-2-deoxyguanosine" OR 8-OHdG OR "glutathione peroxidase" OR GPx OR "GSH-Px" AND SU (random* OR placebo OR "placebo-controlled" OR "double blind" OR "single blind" OR "triple blind" OR trial) NOT SU animal* OR nonhuman* OR murine OR mouse OR mice OR rodent* OR rat OR rats OR porcine OR swine OR pigs OR piglet* OR bovine OR cattle OR ovine OR sheep OR caprine OR goat* OR equine OR horse OR horses OR canine OR dogs OR dog OR feline OR cats OR rabbit* OR hamster* OR "guinea pig*" OR zebrafish OR fish OR fishes OR piscine OR avian OR poultry OR chicken* OR broiler* OR quail* OR duck* OR hens OR hen OR carp OR tilapia OR veterinary |
| CNKI | SU=(' 糖尿病 '+' 胰岛素抵抗 '+' 高血糖 '+' 糖尿病前期 '+' 糖耐量受损 ')(' 硒 '+' 谷胱甘肽过氧化物酶 ')(' 血糖 '+' 糖化血红蛋白 '+' 胰岛素 '+' 降糖 ')-' 鼠 '-TI=' 鼠 ' SU=(' 硒 '+' 谷胱甘肽过氧化物酶 ')*(' 血糖 '+' 糖化血红蛋白 '+' 胰岛素 '+' 降糖 ')-' 鼠 '-TI=' 鼠 ' SU=(' 硒 '+' 谷胱甘肽过氧化物酶 ') and FT=(' 血糖 '+' 糖化血红蛋白 '+' 胰岛素 '+' 降糖 ') and SU=(' 试验 '+' 随机 '+' 盲 '+' 对照 ') not AB=(' 鼠 '+' 鸡 '+' 兔 '+' 猪 '+' 牛 '+' 羊 ') |
| WanFang | #1 主题:(硒 OR 硒代蛋氨酸 OR 亚硒酸钠 OR 硒酸钠 OR 富硒酵母 OR 硒化酵母 OR 硒补充 OR 硒补充剂 OR 膳食硒 OR 强化硒 OR 富硒) #2 主题:(糖化血红蛋白 OR 胰岛素抵抗 OR 胰岛素敏感性 OR 胰岛 β 细胞功能 OR 血糖 OR 抗氧化 OR 氧化应激 OR 丙二醛 OR 谷胱甘肽 OR 超氧化物歧化酶 OR 一氧化氮) #3 主题:(随机 OR 随机对照 OR 随机分组 OR 安慰剂 OR 双盲 OR 单盲 OR 三盲 OR 临床试验 OR 随机对照试验) #4 主题:(鼠 OR 兔 OR 猪 OR 羊 OR 犬 OR 狗 OR 猫 OR 牛 OR 鱼 OR 家禽 OR 鸡 OR 鸭 OR 鹌鹑 OR 鸽 OR 蜂) #5 (#1) AND (#2) AND (#3) NOT (#4) |
| VIP | #1 主题:(硒 OR 硒代蛋氨酸 OR 亚硒酸钠 OR 硒酸钠 OR 富硒酵母 OR 硒化酵母 OR 硒补充 OR 硒补充剂 OR 膳食硒 OR 强化硒 OR 富硒) #2 主题:(糖化血红蛋白 OR 胰岛素抵抗 OR 胰岛素敏感性 OR 胰岛 β 细胞功能 OR 血糖 OR 抗氧化 OR 氧化应激 OR 丙二醛 OR 谷胱甘肽 OR 超氧化物歧化酶 OR 一氧化氮) #3 主题:(随机 OR 随机对照 OR 随机分组 OR 安慰剂 OR 双盲 OR 单盲 OR 三盲 OR 试验 OR 随机对照试验) #4 主题:(鼠 OR 兔 OR 猪 OR 羊 OR 犬 OR 狗 OR 猫 OR 牛 OR 鱼 OR 家禽 OR 鸡 OR 鸭 OR 鹌鹑 OR 鸽 OR 蜂) #5 (#1) AND (#2) AND (#3) NOT (#4) |
| SinoMed | #1 主题:(硒 OR 硒代蛋氨酸 OR 亚硒酸钠 OR 硒酸钠 OR 富硒酵母 OR 硒化酵母 OR 硒补充 OR 硒补充剂 OR 膳食硒 OR 强化硒 OR 富硒)  #2 主题:(糖化血红蛋白 OR 胰岛素抵抗 OR 胰岛素敏感性 OR 胰岛 β 细胞功能 OR 血糖 OR 抗氧化 OR 氧化应激 OR 丙二醛 OR 谷胱甘肽 OR 超氧化物歧化酶 OR 一氧化氮) #3 主题:(随机 OR 随机对照 OR 随机分组 OR 安慰剂 OR 双盲 OR 单盲 OR 三盲 OR 试验 OR 随机对照试验) #4 主题:(鼠 OR 兔 OR 猪 OR 羊 OR 犬 OR 狗 OR 猫 OR 牛 OR 鱼 OR 家禽 OR 鸡 OR 鸭 OR 鹌鹑 OR 鸽 OR 蜂) #5 (#1) AND (#2) AND (#3) NOT (#4) |

*Table footnote:* Database searches yielded 6,165 records (PubMed: 1,004; EMbase: 2,348; Web of Science: 1907; Cochrane Central Register of Controlled Trials: 15; CNKI: 18; WanFang: 244; VIP: 87; CINAHL: 349; SinoMed: 193).

**Additional file 2: Table S2** Excluded articles and reasons for exclusion.

| Title | First author | Public year | Result | Reasons for Exclusion |
| --- | --- | --- | --- | --- |
| Preparation, characterization, antioxidant and hypoglycemic ability of polysaccharide nano-selenium from forsythia suspensa in vitro | Yao LL | 2024 | not included | Animals be regarded as trial subjects |
| Hypoglycemic effect of selenium enriched auricularia auricula polysaccharide | Hao S | 2021 | not included | Animals be regarded as trial subjects |
| The role and mechanisms of selenium supplementation on fatty liver-associated disorder | Xu L | 2022 | not included | Animals be regarded as trial subjects |
| Selenium nanoparticles modulate steroidogenesis-related genes and improve ovarian functions via regulating androgen receptors expression in polycystic ovary syndrome rat model | Abdallah ABE | 2023 | not included | Animals be regarded as trial subjects |
| Study on the hypoglycaemic function of selenium-enriched fermented soy milk | Xu W | 2006 | not included | Animals be regarded as trial subjects |
| Study on the hypoglycaemic effects of nano-selenium-platycodon polysaccharide complexes | Hou W | 2019 | not included | Animals be regarded as trial subjects |
| Invited commentary in response to: Selenium supplementation lowers insulin resistance and markers of cardio-metabolic risk in patients with congestive heart failure: A randomised, double-blind, placebo-controlled trial | Cheng WH | 2018 | not included | Conference thesis or letter |
| The Beneficiary Role of Selenium in Type II Diabetes: A Longitudinal Study | Karalis D | 2019 | not included | Conference thesis or letter |
| The effects of probiotic and selenium co-supplementation on mental health parameters and metabolic profiles in type 2 diabetic patients with coronary heart disease: A randomized, double-blind, placebo-controlled trial | Raygan F | 2019 | not included | Incomplete research data |
| Study on the correlation between micronutrient intake during pregnancy and glycemic control outcomes in patients with gestational diabe | Yuan Z | 2023 | not included | Incomplete research data |
| Effect of selenium preparations on polycystic ovary syndrome: A meta-analysis | Zhao J | 2021 | not included | Incomplete research data |
| Research progress on the antioxidant effects of selenium in diabetes mellitus | Mellitus R | 2023 | not included | Incomplete research data |
| Association between selenium and thyroid hormonesand effect of selenium supplementation on thyroid hor-mones in people with abnormal glucose metabolism | Huang YUE | 2023 | not included | Incomplete research data |
| The relationship between selenium and type 2 diabetes | Ceng C | 2021 | not included | Incomplete research data |
| The effect and mechanism of selenium against manganese exposure on art outcomes in patients with polycystic ovary syndrome | Yin D | 2024 | not included | Incomplete research data |
| Research progress of selenium protein p and gestational diabetes mellitus | Hong WQ | 2021 | not included | Incomplete research data |
| Effect of selenium supplementation on glucose control and insulin sensitivity:A meta-analysis of randomized controlled trials | Huan LZ | 2021 | not included | Incomplete research data |
| Research advances on the trace element selenium in diabetes prevention and treatment | Xiaojun T | 2024 | not included | Incomplete research data |
| Observational study on the efficacy of selenium-enriched nutrient germ rice as a staple food therapy for the prevention and management of type 2 diabetes | Zhou HT | 2021 | not included | Incomplete research data |
| Unraveling the link: Mendelian randomization reveals causal relationship between selenium and metabolic syndrome | Liu F | 2025 | not included | Incomplete research data |
| The effects of selenium supplementation on lipid profile in adults: A systematic review and meta-analysis of randomized controlled trials | Saadh M | 2024 | not included | Incomplete research data |
| The effects of selenium supplementation on blood lipids and blood pressure in adults: A systematic review and dose-response meta-analysis of randomized control trials | Kelishadi M | 2022 | not included | Incomplete research data |
| The impact of mineral supplementation on polycystic ovarian syndrome | Elobeid T | 2022 | not included | Incomplete research data |
| The effects of zinc and selenium supplementation on body composition and thyroid function in individuals with overweight or obesity: A systematic review | Zavros | 2018 | not included | Incomplete research data |
| The effects of selenium supplementation on glucose metabolism and lipid profiles among patients with metabolic diseases: A systematic review and meta-analysis of randomized controlled trials | Tabrizi R | 2017 | not included | Incomplete research data |
| The beneficiary role of selenium in type ii diabetes: A longitudinal study | Karalis D | 2019 | not included | Incomplete research data |
| Selenium, diabetes, and their intricate sex-specific relationship | Demircan K | 2024 | not included | Incomplete research data |
| Selenium supplementation for polycystic ovary syndrome: A meta-analysis of randomized controlled trials | Wu P | 2022 | not included | Incomplete research data |
| Selenium supplementation effect on glycemic control: A grade-assessed systematic review and dose-response meta-analysis of randomized controlled trials | Vajdi M | 2023 | not included | Incomplete research data |
| Selenium supplementation and the risk of type 2 diabetes mellitus: A meta-analysis of randomized controlled trials | Mao S | 2014 | not included | Incomplete research data |
| Selenium biomarkers and mir-7-5p in overweight/obese women | Alves HPM | 2024 | not included | Incomplete research data |
| Selenium and selenoproteins at the intersection of type 2 diabetes and thyroid pathophysiology | Gorini F | 2022 | not included | Incomplete research data |
| Selenium supplementation in pregnancy-maternal and newborn outcomes | Biswas K | 2022 | not included | Incomplete research data |
| Selenium status in patients with chronic liver disease: A systematic review and meta-analysis | Lin Y | 2022 | not included | Incomplete research data |
| Selenium supplementation effect on glycemic control: A GRADE-assessed systematic review and dose-response meta-analysis | Vajdi M | 2023 | not included | Incomplete research data |
| Nutritional supplements and complementary therapies in polycystic ovary syndrome | Alesi S | 2022 | not included | Incomplete research data |
| Nanoparticle-based interventions for polycystic ovary syndrome: A review of mechanisms and therapeutic potential | Muthukumaran D | 2024 | not included | Incomplete research data |
| Genetically predicted selenium is negatively associated with serum tc, ldl-c and positively associated with hba1c levels | Wu Q | 2021 | not included | Incomplete research data |
| Food chemistry of selenium and controversial roles of selenium in affecting blood cholesterol concentrations | Zhao M | 2021 | not included | Incomplete research data |
| Evaluation of the relationship between serum selenium level and glycemic control in pregnant patients* | Akademik | 2023 | not included | Incomplete research data |
| Effects of selenium supplementation on polycystic?Ovarian?Syndrome: A systematic review and meta-analysis on randomized clinical trials | Zhao J | 2023 | not included | Incomplete research data |
| Effects of selenium on coronary artery disease, type 2 diabetes and their risk factors: A mendelian randomization study | Rath AA | 2021 | not included | Incomplete research data |
| Effects of selenium administration on blood lipids: A systematic review and dose-response meta-analysis of experimental human studies | Urbano T | 2025 | not included | Incomplete research data |
| Effects of probiotic and selenium co-supplementation on lipid profile and glycemia indices: A systematic review and meta-analysis of randomized clinical trials | Mohammadparast V | 2023 | not included | Incomplete research data |
| Effectiveness and safety of selenium supplementation for type 2 diabetes mellitus in adults: A systematic review of randomised controlled trials | Stróyk, A | 2019 | not included | Incomplete research data |
| Effect of selenium supplementation on cardiometabolic risk factors in polycystic ovary syndrome (pcos) patients: A systematic review and meta-analysis of randomized clinical trials | Abu-zaida A | 2023 | not included | Incomplete research data |
| Effect of selenium supplementation on glycemic control in patients with type 2 diabetes or prediabetes | Nct05320510 | 2022 | not included | Incomplete research data |
| Effect of selenium supplementation on biochemical markers of women with polycystic ovarian syndrome: A systematic review | Kazempour R | 2023 | not included | Incomplete research data |
| Dose-response relationship between dietary antioxidant intake and diabetic kidney disease in the us adults with diabetes | Zhang J | 2023 | not included | Incomplete research data |
| Could nutrient supplements provide additional glycemic control in diabetes management? A systematic review and meta-analysis of randomized controlled trials of as an add-on nutritional supplementation therapy | Kim Y | 2022 | not included | Incomplete research data |
| Clinical study on the health care effect of selenium-enriched water on impaired fasting blood glucose/type 2 diabetes | Mahdi V | 2025 | not included | Incomplete research data |
| Association of selenium intake and selenium concentrations with risk of type 2 diabetes in adults: A narrative review | Alharithy M | 2023 | not included | Incomplete research data |
| Association between blood selenium levels and gestational diabetes mellitus: A systematic review and meta-analysis | Yan S | 2022 | not included | Incomplete research data |
| Antioxidant supplementation for management of gestational diabetes mellitus in pregnancy: A systematic review and meta-analysis of randomised controlled trials | van der Pligt P | 2025 | not included | Incomplete research data |
| An overview on effects of micronutrients and macronutrients interventions in management of polycystic ovary syndrome | Naz M | 2022 | not included | Incomplete research data |
| A systematic review and dose-response meta-analysis of the effect of selenium supplementation on serum concentration of c-reactive protein | Gholami ALI | 2023 | not included | Incomplete research data |
| A systematic review and dose-response meta-analysis of exposure to environmental selenium and the risk of type 2 diabetes in nonexperimental studies | Vinceti M | 2021 | not included | Incomplete research data |
| Vitamin e and the risk of prostate cancer: The selenium and vitamin e cancer prevention trial (select) | Klein EA | 2011 | not included | Incomplete research data |
| Trace elements in early phase type 2 diabetes mellitus-a population-based study. The hunt study in norway | Hansen AF | 2017 | not included | Incomplete research data |
| Trace element status in patients with type 2 diabetes in norway: The hunt3 survey | Simi A | 2017 | not included | Incomplete research data |
| The influence of adiposity and acute exercise on circulating hepatokines in normal-weight and overweight/obese men | Sargeant JA | 2018 | not included | Incomplete research data |
| The association between dietary selenium intake and diabetes: A cross-sectional study among middle-aged and older adults | Jie WEI | 2015 | not included | Incomplete research data |
| Selenium supplementation for prevention of colorectal adenomas and risk of associated type 2 diabetes | Thompson PA | 2016 | not included | Incomplete research data |
| Randomized, double-blind, placebo-controlled, phase iii chemoprevention trial of selenium supplementation in patients with resected stage i non-small-cell lung cancer: Ecog 5597 | Karp DD | 2013 | not included | Incomplete research data |
| Protectin dx ameliorates palmitate-induced hepatic insulin resistance through ampk/sirt1-mediated modulation of fetuin-a and sep expression | Jung TW | 2019 | not included | Incomplete research data |
| No effect of modest selenium supplementation on insulin resistance in uk pregnant women, as assessed by plasma adiponectin concentration | Mao J | 2016 | not included | Incomplete research data |
| Effect of single nucleotide polymorphisms in seps1 and sepp1 on expression in the protein level in metabolic syndrome in subjects with cardiovascular disease | Gharipour M | 2019 | not included | Incomplete research data |
| Effect of selenium and vitamin e on risk of prostate cancer and other cancers: The selenium and vitamin e cancer prevention trial (select) | Lippman | 2009 | not included | Incomplete research data |
| Effect of long-term treatment with antioxidants (vitamin c, vitamin e, coenzyme q10 and selenium) on arterial compliance, humoral factors and inflammatory markers in patients with multiple cardiovascular risk factors | Shargorodsky M | 2010 | not included | Incomplete research data |
| Can hepatokines be regarded as novel non-invasive serum biomarkers of intrahepatic lipid content in obese children? | Flisiak jackiewicz M | 2019 | not included | Incomplete research data |
| A liver-derived secretory protein, selenoprotein p, causes insulin resistance | Misu H | 2010 | not included | Incomplete research data |
| Serum Selenium Concentrations and Diabetes in U.S. Adults | Laclaustra | 2009 | not included | Incomplete research data |
| Cell: After 200 years! Scientists have finally uncovered the true reason why the trace element selenium is indispensable. However, one should not blindly supplement it. | Wu | 2018 | not included | Incomplete research data |
| Antioxidantsupplementsreducedoxidative stressandstabilizedliverfunctiontestsbut didnotreduceinﬂammationinarandomized controlledtrialinobesechildrenand adolescents | Stefanieb.murer | 2013 | not included | Incomplete research data |
| The Effects of Dietary Selenium Supplementation on Inflammatory Markers Among Patients with Metabolic Diseases | Djalalinia S | 2021 | not included | Incomplete research data |
| Does selenium supplementation increase serum glucoselevels in men at high risk. | Algotar A | 2013 | not included | Incomplete research data |
| Guidelines on the management of stable angina pectoris: Executive summary | Members A | 2006 | not included | Incomplete research data |
| Frequent consumption of selenium-enriched chicken meat by adults causes weight loss and maintains their antioxidant status | Martínez S | 2010 | not included | Incomplete research data |
| Antioxidant supplementation for management of gestational diabetes mellitus in pregnancy: A systematic review and meta-analysis of randomised controlled trials | van der Pligt P | 2025 | not included | Incomplete research data |
| Antioxidant micronutrients in the critically ill: A systematic review and meta-analysis | Manzanares W | 2012 | not included | Incomplete research data |
| Association between blood selenium levels and gestational diabetes mellitus: A systematic review and meta-analysis | Yan S | 2022 | not included | Incomplete research data |
| Selenium supplementation, an adjunct therapy for diabetes and its complications | Zhangda Q | 2020 | not included | Incomplete research data |
| The king of vegetables — spinach the jewel among fruits | Chen L | 2015 | not included | Incomplete research data |
| The favourable effects of long-term selenium supplementation on regression of cervical tissues and metabolic profiles of patients with cervical intraepithelial neoplasia: A randomised, double-blind, placebo-controlled trial | Karamali | 2015 | not included | Incomplete research data |
| Effect of selenium supplementation on changes in glycated haemoglobin (hba1c): Results from a multiple-dose, randomized controlled trial | Stranges-a | 2019 | not included | Incomplete research data |
| Effect of l -arginine and selenium added to a hypocaloric diet enriched with legumes on cardiovascular disease risk factors in women with central obesity: A randomized, double-blind, placebo-controlled trial | Mohammad | 2012 | not included | Incomplete research data |
| Epidemiology of selenium and type 2 diabetes: Can we make sense of it? | Rayman MP | 2013 | not included | Incomplete research data |
| Selenium deficiency is linearly associated with hypoglycemia in healthy adults | Wang Y | 2020 | not included | Incomplete research data |
| Summaries for patients. Long-term use of selenium supplements and risk for type 2 diabetes | No author | 2007 | not included | Incomplete research data |
| Selenium in the treatment of graves’ hyperthyroidism and eye disease | Lanzolla G | 2020 | not included | Incomplete research data |
| The effect of selenium-enriched yeast capsules combined with irbesartan on urinary protein in patients with type 2 diabetic nephropathy | Shen K | 2019 | not included | Incomplete research data |
| Clinical observation of selenium-enriched yeast in patients with autoimmune thyroiditis complicated by hypothyroidism | Fengzong M | 2016 | not included | Incomplete research data |
| The efficacy of selenium in treating 126 cases of type 2 diabetes | Zou WH | 1998 | not included | Incomplete research data |
| Selenium and diabetes | Niyin X | 2000 | not included | Incomplete research data |
| Selenium and coenzyme q(10) supplementation and sex differences in cardiovascular mortality results from a prospective randomized double-blind placebo-controlled trial in elderly people low in selenium | Alehagen U | 2025 | not included | Incomplete research data |
| The effect of selenium supplementation on sonographic findings of salivary glands in papillary thyroid cancer (ptc) patients treated with radioactive iodine: Study protocol for a double-blind, randomized, placebo-controlled clinical trial | Amini S | 2023 | not included | Incomplete research data |
| Investigation of the effect of oral selenium on the reduction of clinical symptoms and joint pain in patients with rheumatoid arthritis in the iranian population | Mehrpooya M | 2023 | not included | Incomplete research data |
| Add-on effect of selenium and vitamin d combined supplementation in early control of graves' disease hyperthyroidism during methimazole treatment | Gallo D | 2022 | not included | Incomplete research data |
| Improved cardiovascular health by supplementation with selenium and coenzyme q10: Applying structural equation modelling (sem) to clinical outcomes and biomarkers to explore underlying mechanisms in a prospective randomized double-blind placebo-controlled intervention project in sweden | Alehagen U | 2022 | not included | Incomplete research data |
| The effects of dietary selenium supplementation on inflammatory markers among patients with metabolic diseases: A systematic review and meta-analysis of randomized controlled trials | Djalalinia S | 2021 | not included | Incomplete research data |
| Supplemental selenium and coenzyme q10 reduce glycation along with cardiovascular mortality in an elderly population with low selenium status - a four-year, prospective, randomised, double-blind placebo-controlled trial | Alehagen U | 2020 | not included | Incomplete research data |
| Consumption of brazil nuts with high selenium levels increased inflammation biomarkers in obese women: A randomized controlled trial | Duarte GBS | 2019 | not included | Incomplete research data |
| Effects of selenium and vitamin c on the serum level of antithyroid peroxidase antibody in patients with autoimmune thyroiditis | Karimi F | 2019 | not included | Incomplete research data |
| Influence of selenium supplementation on patients with inflammation: A pilot double blind randomized study | Freitas RGBON | 2017 | not included | Incomplete research data |
| The effect of selenium supplementation on acute phase reactants and thyroid function tests in hemodialysis patients | Omrani HR | 2015 | not included | Incomplete research data |
| Protective effect of selenium on cisplatin induced nephrotoxicity: A double-blind controlled randomized clinical trial | Ghorbani A | 2013 | not included | Incomplete research data |
| Effect of selenium on immune response against hepatitis b vaccine with accelerated method in insulin-dependent diabetes mellitus patients | Janbakhsh A | 2013 | not included | Incomplete research data |
| Selenium and the thyroid | Khrle J | 2013 | not included | Incomplete research data |
| Restoration of antioxidant enzymes in the therapeutic use of selenium in septic patients | Janka V | 2013 | not included | Incomplete research data |
| Selenium supplementation for the primary prevention of cardiovascular disease | Rees K | 2013 | not included | Incomplete research data |
| Phase iii trial of selenium to prevent prostate cancer in men with high-grade prostatic intraepithelial neoplasia: Swog s9917 | Marshall JR | 2011 | not included | Incomplete research data |
| Selenium supplementation reduced oxidative dna damage in adnexectomized brca1 mutations carriers | Dziaman T | 2009 | not included | Incomplete research data |
| Effect of supplementation with se-enriched yeast and factors influencing se concentration in plasma of transplant recipients | Bost M | 2009 | not included | Incomplete research data |
| Randomised, double blind, placebo-controlled trial of selenium supplementation in adult asthma | Shaheen SO | 2007 | not included | Incomplete research data |
| The effect of antioxidant supplementation on superoxide dismutase activity, cu and zn levels, and total antioxidant status in erythrocytes of patients with graves' disease | Bacic-vrca V | 2005 | not included | Incomplete research data |
| The effect of vitamin-mineral supplementation on the level of mda and activity of glutathione peroxidase and superoxide dismutase in blood of matched maternal-cord pairs | Che?chowska M | 2004 | not included | Incomplete research data |
| [investigation of antioxidant therapy with sodium selenite in acute pancreatitis. A prospective randomized blind trial] | Lindner D | 2004 | not included | Incomplete research data |
| Selenium supplementation in patients with autoimmune thyroiditis decreases thyroid peroxidase antibodies concentrations | G?rtner R | 2002 | not included | Incomplete research data |
| Protein carbonyls and lipid peroxidation products as oxidation markers in preterm infant plasma: Associations with chronic lung disease and retinopathy and effects of selenium supplementation | Winterbourn CC | 2000 | not included | Incomplete research data |
| Effects of organic and inorganic selenium supplementation on selenoenzyme activity in blood lymphocytes, granulocytes, platelets and erythrocytes | Brown KM | 2000 | not included | Incomplete research data |
| [effects of selenium supplement on acute lower respiratory tract infection caused by respiratory syncytial virus] | Liu X | 1997 | not included | Incomplete research data |
| Selenium-dependent gsh-px in erythrocytes of patients with hypertension treated with ace inhibitors | Djordjevi VB | 1998 | not included | Incomplete research data |
| Supplementary selenium influences the response to fatty acid-induced oxidative stress in humans | Meltzer HM | 1997 | not included | Incomplete research data |
| Selenate fortification of infant formulas improves the selenium status of preterm infants | Tyrala EE | 1996 | not included | Incomplete research data |
| Randomised clinical trial of parenteral selenium supplementation in preterm infants | Daniels L | 1996 | not included | Incomplete research data |
| Selenium supplementation enhances the element concentration in blood and seminal fluid but does not change the spermatozoal quality characteristics in subfertile men | Iwanier K | 1995 | not included | Incomplete research data |
| Effect of double-blind cross-over selenium supplementation on lipid peroxidation markers in cystic fibrosis patients | Portal B | 1995 | not included | Incomplete research data |
| Mitochondria changes in human muscle after prolonged exercise, endurance training and selenium supplementation | Zamora AJ | 1995 | not included | Incomplete research data |
| Selenate fortification improves selenium status of term infants fed soy formula | Smith AM | 1995 | not included | Incomplete research data |
| Increase in oxidation resistance of atherogenic serum lipoproteins following antioxidant supplementation: A randomized double-blind placebo-controlled clinical trial | Nyyssnen K | 1994 | not included | Incomplete research data |
| Selenium supplementation in intrinsic asthma | Hasselmark L | 1993 | not included | Incomplete research data |
| Vitamin e and selenium in preterm infants: Lack of effect on clinical patency of ductus arteriosus | Rudolph N | 1989 | not included | Incomplete research data |
| The effect of supplementation with selenium and vitamin e in psoriasis | Fairris GM | 1989 | not included | Incomplete research data |
| The effect on atopic dermatitis of supplementation with selenium and vitamin e | Fairris GM | 1989 | not included | Incomplete research data |
| Selenium supplementation does not alter platelet activation in subjects with normal selenium status | Mutanen M | 1989 | not included | Incomplete research data |
| Improved erythrocyte survival with combined vitamin e and selenium therapy in children with glucose-6-phosphate dehydrogenase deficiency and mild chronic hemolysis | Hafez M | 1986 | not included | Incomplete research data |
| Serum fatty acids, apolipoproteins, selenium and vitamin antioxidants and the risk of death from coronary artery disease | Salonen JT | 1985 | not included | Incomplete research data |
| Effect of ascorbic acid supplementation on selenium bioavailability in humans | Mutanen M | 1985 | not included | Incomplete research data |
| A randomized, double blind, placebo controlled clinical trial of supplementation of l-selenomethionine in patients with prostate cancer prior to prostatectomy or brachytherapy (se pre-prostatectomy/pre-brachytherapy trial) | No author | 2008 | not included | Incomplete research data |
| Behavior of selected antioxidant and immune parameters in selenium-supplemented healthy subjects | Herzfeld A | 1990 | not included | Incomplete research data |
| Challenges and perspectives of selenium supplementation in graves’ disease and orbitopathy | Bednarczuk T | 2020 | not included | Incomplete research data |
| Effect of selenium supplementation on inflammatory,oxidative and nutritional markers in hemodialysis patients | No author | 2010 | not included | Incomplete research data |
| Effect of selenium supplementation on mild graves' ophthalmopathy at a tertiary hospital - a six-month open-labelled, assessor-masked randomized-controlled trial | Flores J | 2024 | not included | Incomplete research data |
| Effects of selenium supplement on acute lower respiratory tract infection caused by respiratory syncytial virus | Liu X | 1997 | not included | Incomplete research data |
| Effects of selenium supplementationonserum thyroid peroxidase antibody levels in patients with autoimmune thyroiditis | Neto J | 2015 | not included | Incomplete research data |
| Impact of selenium supplementation on oxidative stress in patient after cardiac arrest | Janotka M | 2019 | not included | Incomplete research data |
| Monoselenolipoic acid may be an outstanding pharmaceutical antioxidant with direct thioredoxin-like activity | Mccarty | 2000 | not included | Incomplete research data |
| Nano-selenium and the src family kinases pathway: Redefining gene expression dynamics in major depressive disorder based on a randomized controlled trial | Noormohammadi M | 2024 | not included | Incomplete research data |
| Phase i multiple dose study of 12-week treatment by se-methyl-l-cysteine(msc) and l semet in adult males | No author | 2011 | not included | Incomplete research data |
| Prospective associations between plasma saturated, monounsaturated and polyunsaturated fatty acids and overall and breast cancer risk - modulation by antioxidants: A nested case-control study | Pouchieu C | 2014 | not included | Incomplete research data |
| Prospective study of serum cysteine levels and oesophageal and gastric cancers in china | Murphy G | 2011 | not included | Incomplete research data |
| Prospective, multicenter, randomized, double-blind, placebo-controlled study evaluating efficacy and safety of a therapeutic administration of selenium, as selenite, in septic shock patients | No author | 2005 | not included | Incomplete research data |
| Selenistasis: Epistatic effects of selenium on cardiovascular phenotype | Joseph J | 2013 | not included | Incomplete research data |
| Selenium and diabetes: More bad news for supplements | Bleys J | 2007 | not included | Incomplete research data |
| Selenium and the thyroid gland: More good news for clinicians | Drutel A | 2013 | not included | Incomplete research data |
| Selenium for malnutrition in hemodialysis patients: Have we considered all of the elements? | Thompson S | 2013 | not included | Incomplete research data |
| Selenium supplementation and modified high-intensity interval training (m-hiit) to modulate systemic oxidative stress in sedentary overweight/obese adults: In vitro and in vivo studies | Leighton D | 2017 | not included | Incomplete research data |
| The effect of selenium supplementation among pediatric patients with burns | No author | 2009 | not included | Incomplete research data |
| The effect of selenium therapy on autoimmune thyroiditis | Balázs | 2008 | not included | Incomplete research data |
| The effect of vitamin-mineral supplementation on the level of mda and activity of glutathione peroxidase and superoxide dismutase in blood of matched maternal-cord pairs | Che?chowska M | 2004 | not included | Incomplete research data |
| The role of natrium selenite as adjuvants in radiotherapy of rectal cancer | Hehr T | 1997 | not included | Incomplete research data |
| Evaluating the antioxidative activity of diselenide containing compounds in human blood | Saluk, J | 2013 | not included | Incomplete research data |
| Effect of selenium supplementation on recurrent hyperthyroidism caused by graves' disease: A prospective pilot study | Wang, L | 2016 | not included | Incomplete research data |
| Selenium supplementation in low-birth-weight premature-infants - relationship to trace-metals and antioxidant enzymes | Huston, RK | 1991 | not included | Incomplete research data |
| Effects of selenomethionine supplementation on selenium status and thyroid hormone concentrations in healthy adults | Combs, GF | 2009 | not included | Incomplete research data |
| Effect modification of selenium supplementation by intake and serum concentrations of antioxidants on the development of metachronous colorectal adenoma | Jacobs, ET | 2023 | not included | Incomplete research data |
| Selenium and cancer:: Biomarkers of selenium status and molecular action of selenium supplements | Gromadzinska, J | 2008 | not included | Incomplete research data |
| Selenium status of new-zealand infants fed either a selenium supplemented or a standard formula | Darlow, BA | 1995 | not included | Incomplete research data |
| Potential benefits of selenium supplementation in patients with kidney disease | Badri, S | 2021 | not included | Incomplete research data |
| Selenium supplementation in the diets of patients suffering from ulcerative colitis | Stedman, JD | 1997 | not included | Incomplete research data |
| Selenium as a modulator of redox reactions in the prevention and treatment of cardiovascular diseases | Leszto, K | 2024 | not included | Incomplete research data |
| Serum cd5l responds positively to selenium and coenzyme q10 supplementation with relation to thyroid hormones, mortality, and health-related quality-of-life-a sub-analysis of a double-blind randomised placebo-controlled trial in elderly low in selenium | Alehagen, U | 2025 | not included | Incomplete research data |
| Effects of an intervention with selenium and coenzyme q10 on five selected age-related biomarkers in elderly swedes low in selenium: Results that point to an anti-ageing effect-a sub-analysis of a previous prospective double-blind placebo-controlled randomised clinical trial | Alehagen, U | 2023 | not included | Incomplete research data |
| Selenium and zinc: "Antioxidants" For healthy aging? | Steinbrenner, H | 2020 | not included | Incomplete research data |
| Selenium supplementation and autoimmune thyroid diseases | Sturniolo G | 2013 | not included | Incomplete research data |
| Spectacular reduction in the mortality of acutely injured patients by the administration of vitamins c and e and selenium | Hemil | 2009 | not included | Incomplete research data |
| Cardiac and red blood cell glutathione peroxidase: Results of a prospective randomized trial in patients on total parenteral nutrition | Batist, G | 1985 | not included | Incomplete research data |
| Study on the effects of selenium on thyroid peroxidase antibodies and antioxidant capacity in patients with autoimmune thyroiditis | Bai X | 2010 | not included | Incomplete research data |
| The hypotensive effect of selenium | No author | 2000 | not included | Incomplete research data |
| The effect of trace element selenium on lipid peroxidation damage in paediatric patients with acute viral myocarditis | Zhengbai H | 2004 | not included | Incomplete research data |
| The effect of selenium on autoantibodies in autoimmune thyroiditis | Zhu L | 2010 | not included | Incomplete research data |
| Clinical study on the application of selenium-enriched yeast in the treatment of diabetic retinopathy | Tian L | 2020 | not included | Incomplete research data |
| A randomised controlled trial of selenium on antioxidant capacity in subclinical keshan disease | Liuzeng C | 2011 | not included | Incomplete research data |
| A randomised controlled trial of selenium on prognosis and protection against myocardial oxidative damage in patients with slow-type keshan disease | Lan T | 2012 | not included | Incomplete research data |
| The effect of selenium on glutathione peroxidase (gpx) gene expression in patients with rheumatic heart disease | Chang Q | 2001 | not included | Incomplete research data |
| Changes in blood lipid peroxidation intensity, selenocysteine peroxidase activity, and vitamin e content in non-insulin-dependent diabetes mellitus patients | Hu SW | 1989 | not included | Incomplete research data |
| Observations on the preventive and therapeutic effects of selenium-enriched wholemeal flour on hyperglycaemia and hyperlipidaemia | Li MS | 2014 | not included | Incomplete research data |
| Relationship between blood glucose levels and serum selenium and vitamin e in elderly diabetics | Miao HY | 1998 | not included | Incomplete research data |
| Selenium-yeast combined with levothyroxine therapy for type 2 diabetes mellitus with hashimoto's thyroiditis | Wang JF | 2016 | not included | Incomplete research data |
| Correlation between abnormal changes in serum trace elements and adipokines in high-risk diabetic populations and blood glucose levels | Duren S | 2017 | not included | Incomplete research data |
| Trace element selenium and impaired glucose tolerance | Chenxiao P | 2000 | not included | Incomplete research data |
| Study on the effect of selenium on the course of mycoplasma pneumoniae pneumonia | Hushan M | 1998 | not included | Incomplete research data |
| Observational study on the therapeutic effect of selenium in mycoplasma pneumonia | Liuxiao H | 1998 | not included | Incomplete research data |
| Observational study on the therapeutic effects of selenium and vitamin a in mycoplasma pneumonia | Liuxiao H | 1997 | not included | Incomplete research data |
| Observation of the effect of selenium supplementation on acute respiratory syncytial virus lower respiratory tract infections | Liuxiao H | 1997 | not included | Incomplete research data |
| Effects of selenium nutritional supplement of glucose, nutritional status, and clinical outcome among type 2 diabetes patients | No author | 2024 | not included | Incomplete research data |
| Effect of selenium supplementation on serum adiponectin and homocystein | No author | 2010 | not included | Incomplete research data |
| Metabolism of selenium by pregnant women | Butler J | 1992 | not included | Incomplete research data |
| Selenium supplementation of patients with cirrhosis | No author | 2005 | not included | Incomplete research data |
| Selenium substitution in critical ill patients | Grtner R | 1997 | not included | Incomplete research data |
| The effect of selenium supplementation on anti-oxidant levels in copd patients: A 12-week, randomized, placebo-controlled trial | No author | 2005 | not included | Incomplete research data |
| The effect of sodium selenite substitution on oxidative stress markers and antioxidants in severe sepsis | Woth G | 2013 | not included | Incomplete research data |
| Therapeutic effect of sodium selenite on oxidative stress in patients with severe sepsis | No author | 2013 | not included | Incomplete research data |
| A study on the therapeutic effects of selenium in cataract treatment | Tansong N | 1995 | not included | Incomplete research data |
| The effect of selenium supplementation on oxidative stress, clinical symptoms and mental health status in patients with migraine: A study protocol for a double-blinded randomized clinical trial | Balali A | 2024 | not included | Incomplete research data |
| The effects of probiotic and selenium co-supplementation on parameters of mental health, hormonal profiles, and biomarkers of inflammation and oxidative stress in women with polycystic ovary syndrome | Jamilian M | 2018 | not included | Incomplete research data |
| The effect of na-selenite treatment on the oxidative stress-antioxidants balance of multiple organ failure | Woth G | 2014 | not included | Incomplete research data |
| Selenium supplementation and exercise: Effect on oxidant stress in overweight adults | Savory LA | 2012 | not included | Incomplete research data |
| The influence of selenium-enriched milk proteins and selenium yeast on plasma selenium levels and rectal selenoprotein gene expression in human subjects | Hu Y | 2011 | not included | Incomplete research data |
| Effect of vitamin e and selenium supplementation on oxidative stress status in pulmonary tuberculosis patients | Seyedrezazadeh E | 2008 | not included | Incomplete research data |
| A short-term intervention trial with selenate, selenium-enriched yeast and selenium-enriched milk: Effects on oxidative defence regulation | Ravn-haren G | 2008 | not included | Incomplete research data |
| [influence of oral sodium selenite and vitamin e on thyroid hormones in patients with cardiovascular disease at altitude] | Jin M | 2004 | not included | Incomplete research data |
| Influence of selenium-enriched yeast supplementation on biomarkers of oxidative damage and hormone status in healthy adult males: A clinical pilot study | El-bayoumy K | 2002 | not included | Incomplete research data |
| Selenium supplementation of children in a selenium-deficient area in china: Blood selenium levels and glutathione peroxidase activities | Alfthan G | 2000 | not included | Incomplete research data |
| Dietary selenium increases cellular glutathione peroxidase activity and reduces the enhanced susceptibility to lipid peroxidation of plasma and low-density lipoprotein in kidney transplant recipients | Hussein O | 1997 | not included | Incomplete research data |
| High dose selenium effects on oxidative stress and inflammatory markers in post gastrointestinal surgery state: A randomized trial in critically ill phildren | Roudi | 2020 | not included | Incomplete research data |
| The effect of selenium supplementation on glucose homeostasis and the expression of genes related to glucose metabolism | Jablonska | 2016 | not included | Incomplete research data |
| The effects of a novel combination of selenium and probiotic on weight loss, glycemic control and markers of cardio-metabolic risk in women with polycystic ovary syndrome | Shabani | 2018 | not included | Incomplete research data |
| Effects of selenium and/or n-acetyl-cysteine supplementation on nonthyroidal illness syndrome in hemodialysis patients: A factorial randomized controlled trial | Shahreki | 2022 | not included | Incomplete research data |
| An investigation into the therapeutic effects of selenium on cataracts | Tan SN | 1995 | not included | Incomplete research data |
| The effect of sodium selenite on serum thyroid peroxidase antibody titres and oxidative stress levels in patients with hashimoto's thyroiditis | Wang QH | 2017 | not included | Incomplete research data |
| Effects of oral sodium selenite and vitamin e on thyroid hormones in cardiovascular disease patients in high-altitude regions | Jin M | 2004 | not included | Incomplete research data |
| Modulation of blood antioxidant function in parkinson's disease patients by selenium and vitamins e and c | Liu Y | 2006 | not included | Incomplete research data |
| The effect of selenium supplementation on serum selenium, cadmium, and lipid peroxide levels in hypertensive patients | Zhang SY | 2003 | not included | Incomplete research data |
| The effect of selenium-enriched yeast tablets combined with levothyroxine sodium on blood glucose levels and serum tpoab and tgab levels in patients with type 2 diabetes and chronic lymphocytic thyroiditis | Yan XD | 2021 | not included | Incomplete research data |
| The effects of selenium supplementation on gene expression related to insulin and lipid metabolism, and pregnancy outcomes in patients with gestational diabetes mellitus: A randomized, double-blind, placebo-controlled trial | Karamali M | 2020 | not included | Incomplete research data |
| A systematic review and dose-response meta-analysis of exposure to environmental selenium and the risk of type 2 diabetes in nonexperimental studies | Vinceti M | 2021 | not included | Incomplete research data |
| A randomised trial of selenium supplementation and type-2 diabetes risk, as assessed by plasma adiponectin | Rayman | 2012 | not included | Incomplete research data |
| Effect of selenium supplementation on lipid profile, anemia, and inflammation indices in hemodialysis patients | Assarzadeh S | 2022 | not included | Incomplete research data |
| Effects of selenium supplementation on paraoxonase-1 and myeloperoxidase activity in subjects with cardiovascular disease: The selenegene study, a double-blind randomized controlled trial | Mirmohammadsadeghi A | 2018 | not included | Incomplete research data |
| Selenium supplementation in patients undergoing hematopoietic stem cell transplantation: Effects on pro-inflammatory cytokines levels | Daeian N | 2014 | not included | Incomplete research data |
| Effect of long-term selenium yeast intervention on activity and gene expression of antioxidant and xenobiotic metabolising enzymes in healthy elderly volunteers from the danish prevention of cancer by intervention by selenium (precise) pilot study | G-a R | 2008 | not included | Incomplete research data |
| Selenium supplementation of chinese women with habitually low selenium intake increases plasma selenium, plasma glutathione peroxidase activity, and milk selenium, but not milk glutathione peroxidase activity | Moore MA | 2000 | not included | Incomplete research data |
| The enzymatic antioxidant system in blood and glutathione status in human immunodeficiency virus (hiv)-infected patients: Effects of supplementation with selenium or beta-carotene | Delmas-beauvieux MC | 1996 | not included | Incomplete research data |
| Selenium supplementation in healthy belgian adults: Response in platelet glutathione peroxidase activity and other blood indices | Nève J | 1988 | not included | Incomplete research data |
| Serum selenium and glutathione peroxidase, and plasma lipid peroxides in uterine, ovarian or vulvar cancer, and their responses to antioxidants in patients with ovarian cancer | H-a S | 1984 | not included | Incomplete research data |
| Bioavailability of selenium to finnish men as assessed by platelet glutathione peroxidase activity and other blood parameters | Oa-a L | 1983 | not included | Incomplete research data |
| Influence of selenium supplementation to renal allograft recipients on plasma glutathione peroxidase activity | Wlodarczyk | 2005 | not included | Incomplete research data |
| Effects of selenium supplementation on gene expression levels of inflammatory cytokines and vascular endothelial growth factor in patients with gestational diabetes (publication with expression of concern) | Jamilian | 2018 | not included | Incomplete research data |
| Randomised controlled trial of the effect of long-term selenium supplementation on plasma cholesterol in an elderly danish population | Cold | 2015 | not included | Incomplete research data |
| Selenium supplementation improves the nutritional status of hemodialysis patients: A randomized, double-blind, placebo-controlled trial? | Salehi | 2013 | not included | Incomplete research data |
| In vitro and in vivo studies observe selenium supplementation improving antioxidant enzyme activity: Research on selenium therapy for coronary heart disease | Dd-a X | 2011 | not included | Incomplete research data |
| The effect of selenium on glutathione peroxidase gene expression in patients with rheumatic heart disease | Chang Q | 2003 | not included | Incomplete research data |
| Clinical efficacy of selenium in hashimoto's thyroiditis and its effects on the oxidative-antioxidant system | Ll-a Z | 2019 | not included | Incomplete research data |
| Serum selenoprotein P predicts future hyperglycemia | Oo SM | 2018 | not included | Incomplete research data |
| Selenium supplementation and insulin resistance in a randomized, clinical trial | T J | 2019 | not included | Incomplete research data |
| Selenium supplementation and gestational diabetes: A randomised controlled trial | Yigit E | 2024 | not included | Incomplete research data |
| Effects of selenium intake on the expression of prostaglandin-endoperoxide synthase 2 (cyclooxygenase-2) and matrix metallopeptidase-9 genes in the coronary artery disease: Selenegene study, a double-blind randomized controlled trial | Gharipour M | 2021 | not included | Incomplete research data |
| Effect of selenium supplementation on the levels of gene expression associated with insulin and lipid metabolism, as well as inflammatory markers, in diabetic hemodialysis patients | Jamal A | 2022 | not included | Incomplete research data |
| Selenium and iodine supplementation: Effect on thyroid function of older new zealanders | Thomson CD | 2009 | not included | Incomplete research data |
| Effect of selenomethionine- enriched yeast on hypothyroidism patients | Hajyasini A | 2025 | not included | Incomplete research data |
| Auxiliary hypoglycemic effect of bitter melon extract, selenium yeastand chromium yeast composition | Zhang X | 2024 | not included | Lack of a selenium supplement control group |
| Preparation of polygonatum selenomannan-glycoprotein complexes and investigation of their in vitro hypoglycaemic activity | Li YX | 2025 | not included | Lack of a selenium supplement control group |
| Clinical observation of selenium-rich qingqian salix yellow essence tea on blood glucose regulation in diabetic patients | Shao XT | 2023 | not included | Lack of a selenium supplement control group |
| The effects of selenium-enriched qingqian willow yellow essence tea on reducing hba1c in diabetic patients | Shao XT | 2024 | not included | Lack of a selenium supplement control group |
| The effects of zinc and selenium co-supplementation on resting metabolic rate, thyroid function, physical fitness, and functional capacity in overweight and obese people under a hypocaloric diet: A randomized, double-blind, and placebo-controlled trial | Zavros A | 2023 | not included | Lack of a selenium supplement control group |
| Associations between urinary and dietary selenium and blood metabolic parameters in a healthy northern italy population | Urbano T | 2021 | not included | Lack of a selenium supplement control group |
| Zinc and selenium levels in women with gestational diabetes mellitus at medani hospital, sudan | Hamdan HZ | 2014 | not included | Lack of a selenium supplement control group |
| The correlation between dietary selenium intake and type 2 diabetes: A cross-sectional population-based study on north chinese adults | Siddiqi SM | 2020 | not included | Lack of a selenium supplement control group |
| Gestational hyperglycemia, zinc, selenium, and antioxidant vitamins | Bo S | 2005 | not included | Lack of a selenium supplement control group |
| Dietary selenium intake and type-2 diabetes: A cross-sectional population-based study on cume project | Dias JPV | 2021 | not included | Lack of a selenium supplement control group |
| Dietary copper and selenium intakes and the risk of type 2 diabetes mellitus: Findings from the china health and nutrition survey | Cui Z | 2022 | not included | Lack of a selenium supplement control group |
| Comparative effects of two different forms of selenium on oxidative stress biomarkers in healthy men: A randomized clinical trial | Jr R | 2014 | not included | Lack of a selenium supplement control group |
| Association between selenium intake, diabetes and mortality in adults: Findings from national health and nutrition examination survey (nhanes) 2003-2014 | Hoque B | 2022 | not included | Lack of a selenium supplement control group |
| A prospective study of dietary selenium intake and risk of type 2 diabetes | Stranges S | 2010 | not included | Lack of a selenium supplement control group |
| Probiotic and selenium co-supplementation, and the effects on clinical, metabolic and genetic status in alzheimer's disease: A randomized, double-blind, controlled trial. | Omidrezatamtajia | 2018 | not included | Lack of a selenium supplement control group |
| Comparison of whole blood selenium values and erythrocyte glutathione peroxidase activities of normal individuals on supplementation with selenate, selenite, l-selenomethionine, and high selenium yeast | Clausen J | 1988 | not included | Lack of a selenium supplement control group |
| Effects of supplementation with selenium, as selenized yeast, in a healthy male population from new zealand | Karunasinghe | 2013 | not included | Lack of a selenium supplement control group |
| Selenium supplementation and changes in blood rheology | Tang J | 2008 | not included | Lack of a selenium supplement control group |
| No effect of selenium supplementation on serum glucose levels in men with prostate cancer | Algotar AM | 2010 | not included | Repeated studies (select recent publication year) |
| Maternal-fetal status of copper, iron, molybdenum, selenium and zinc in patients with gestational diabetes | Al-saleh E | 2004 | not included | Repeated studies (select recent publication year) |
| Effects of selenate polysaccharides on serum malondialdehyde levels and cuzn-sod activity in cancer patients | Sz-a H | 1997 | not included | Repeated studies (select recent publication year) |
| Selenium and glutathione levels, and glutathione peroxidase activities in blood components of uremic patients on hemodialysis supplemented with selenium and treated with erythropoietin | Zachara BA | 2001 | not included | Studying on blood selenium rather than selenium supplementation |
| Long-term supplementation with selenate and selenomethionine: Selenium and glutathione peroxidase (ec 1.11.1.9) in blood components of new zealand women | Cd-a T | 1993 | not included | Studying on blood selenium rather than selenium supplementation |
| Selenium metabolism and platelet glutathione peroxidase activity in healthy finnish men: Effects of selenium yeast, selenite, and selenate | G-a A | 1991 | not included | Studying on blood selenium rather than selenium supplementation |
| Selenium status and the effect of organic and inorganic selenium supplementation in a group of elderly people in denmark | Lassen | 1994 | not included | Studying on blood selenium rather than selenium supplementation |
| Research advances on serum selenium and type 2 diabetes-associated sarcopenia | Liu Q | 2024 | not included | Studying on blood selenium rather than selenium supplementation |
| Relationship between serum sepp, nesfatin - 1, tnf - α levels and insulin resistance in patients with gestational diabetes. | Min. S | 2022 | not included | Studying on blood selenium rather than selenium supplementation |
| Research on the correlation between serum selenium level in early pregnancy and gestational diabetes mellitus | Jinmei Y | 2025 | not included | Studying on blood selenium rather than selenium supplementation |
| Changes in serum selenium, calcium and lactone levels in pregnant women with gestational diabetes during early pregnancy and their significance | Yang Y | 2024 | not included | Studying on blood selenium rather than selenium supplementation |
| Influencing factors of postpartum abnormal glucose metabolism in pregnant women with gestational diabetes mellitus and the predictive value of serum selenoprotein p during second trimester | Ai-feng L | 2021 | not included | Studying on blood selenium rather than selenium supplementation |
| Effect of selenium supplementation on insulin resistance and hs-crp in patients with early onset type 2 diabetes | Guo-yan C | 2021 | not included | Studying on blood selenium rather than selenium supplementation |
| The role of selenium in type-2 diabetes mellitus and its metabolic comorbidities | Steinbrenner H | 2022 | not included | Studying on blood selenium rather than selenium supplementation |
| Serum selenium, selenoprotein p, and glutathione peroxidase 3 during early and late pregnancy in association with gestational diabetes mellitus: Prospective odense child cohort | Demircan K | 2023 | not included | Studying on blood selenium rather than selenium supplementation |
| Serum selenium levels and their relationship with insulin resistance in individuals with polycystic ovary syndrome | Karami M | 2025 | not included | Studying on blood selenium rather than selenium supplementation |
| Plasma selenium and the risk of first stroke in adults with hypertension: A secondary analysis of the china stroke primary prevention trial | Wang Z | 2022 | not included | Studying on blood selenium rather than selenium supplementation |
| Novel strategies for assessing associations between selenium biomarkers and cardiometabolic risk factors: Concentration, visit-to-visit variability, or individual mean? Evidence from a repeated-measures study of older adults with high selenium | Li A | 2022 | not included | Studying on blood selenium rather than selenium supplementation |
| No effect of modest selenium supplementation on insulin resistance in uk pregnant women, as assessed by plasma adiponectin concentration | Mao J | 2015 | not included | Studying on blood selenium rather than selenium supplementation |
| Higher levels of blood selenium are associated with higher levels of serum lipid profile in us adults with ckd: Results from nhanes 2013-2018 | Li Z | 2023 | not included | Studying on blood selenium rather than selenium supplementation |
| Comprehensive insights into the health effects of selenium exposure and supplementation among the chinese community middle-aged and elderly: A combined retrospective cohort study and intervention study | Li C | 2024 | not included | Studying on blood selenium rather than selenium supplementation |
| Association of serum selenium with clinical features and inflammatory and oxidative stress markers in iranian patients with metabolic dysfunction-associated steatotic liver disease-a cross-sectional study | Pishdadian A | 2025 | not included | Studying on blood selenium rather than selenium supplementation |
| Association between selenium level in blood and glycolipid metabolism in residents of enshi prefecture, china | Fang H | 2021 | not included | Studying on blood selenium rather than selenium supplementation |
| [selenoprotein p in type 2 diabetes mellitus and its association with insulin resistance] | Pan J | 2014 | not included | Studying on blood selenium rather than selenium supplementation |
| The role of afamin and other trace elements in the prediction of gdm: A tertiary center experience | Erolu H | 2021 | not included | Studying on blood selenium rather than selenium supplementation |
| The relationship between heavy metal exposure, trace element level, and monocyte to hdl cholesterol ratio with gestational diabetes mellitus | Onat T | 2021 | not included | Studying on blood selenium rather than selenium supplementation |
| The predictive value of selenium in diagnosis of gestational diabetes: A nested case-control study | Moshfeghy Z | 2020 | not included | Studying on blood selenium rather than selenium supplementation |
| The association of circulating selenium concentrations with diabetes mellitus | Liao XL | 2020 | not included | Studying on blood selenium rather than selenium supplementation |
| Serum selenoprotein p, but not selenium, predicts future hyperglycemia in a general japanese population | Oo SM | 2018 | not included | Studying on blood selenium rather than selenium supplementation |
| Serum selenoprotein p levels in patients with type 2 diabetes and prediabetes: Implications for insulin resistance, inflammation, and atherosclerosis | Yang SJ | 2011 | not included | Studying on blood selenium rather than selenium supplementation |
| Serum selenium in relation to measures of glucose metabolism and incidence of type?2 diabetes in an older swedish population | Gao H | 2014 | not included | Studying on blood selenium rather than selenium supplementation |
| Serum selenium concentrations correlate significantly with inflammatory biomarker high-sensitive crp levels in hungarian gestational diabetic and healthy pregnant women at mid-pregnancy | Molnar J | 2008 | not included | Studying on blood selenium rather than selenium supplementation |
| Serum selenium concentrations and diabetes in u.s. Adults: National health and nutrition examination survey (nhanes) 2003-2004 | Laclaustra M | 2009 | not included | Studying on blood selenium rather than selenium supplementation |
| Serum selenium and diabetes in u.s. Adults | Bleys J | 2007 | not included | Studying on blood selenium rather than selenium supplementation |
| Selenoprotein p is not elevated in gestational diabetes mellitus | Altinova AE | 2015 | not included | Studying on blood selenium rather than selenium supplementation |
| Selenoprotein p is elevated in individuals with obesity, but is not independently associated with insulin resistance | Chen M | 2017 | not included | Studying on blood selenium rather than selenium supplementation |
| Selenoprotein p in patients with nonalcoholic fatty liver disease | Polyzos SA | 2019 | not included | Studying on blood selenium rather than selenium supplementation |
| Selenium levels and glutathione peroxidase activity in the plasma of patients with type ii diabetes mellitus | R G | 2016 | not included | Studying on blood selenium rather than selenium supplementation |
| Selenium and other elements in human maternal and umbilical serum, as determined simultaneously by proton-induced x-ray emission | Hyv?nen-dabek M | 1984 | not included | Studying on blood selenium rather than selenium supplementation |
| Selenium status is associated with insulin resistance markers in adults: Findings from the 2013 to 2018 national health and nutrition examination survey (nhanes) | Cardoso BR | 2021 | not included | Studying on blood selenium rather than selenium supplementation |
| Selenium levels in community dwellers with type 2 diabetes mellitus | Zhang Q | 2019 | not included | Studying on blood selenium rather than selenium supplementation |
| Sep is elevated in nafld and participates in nafld pathogenesis through ampk/acc pathway | Chen Y | 2021 | not included | Studying on blood selenium rather than selenium supplementation |
| Relationship between selenium and hematological markers in young adults with normal weight or overweight/obesity | Larvie DY | 2019 | not included | Studying on blood selenium rather than selenium supplementation |
| Plasma selenoproteins concentrations in type 2 diabetes mellitus--a pilot study | Roman M | 2010 | not included | Studying on blood selenium rather than selenium supplementation |
| Plasma levels of trace element status in early pregnancy and the risk of gestational diabetes mellitus: A nested case-control study | Zhu G | 2021 | not included | Studying on blood selenium rather than selenium supplementation |
| Maternal-foetal status of copper, iron, molybdenum, selenium and zinc in obese gestational diabetic pregnancies | Al-saleh E | 2007 | not included | Studying on blood selenium rather than selenium supplementation |
| Levels of circulating selenoprotein p, fibroblast growth factor (fgf) 21 and fgf23 in relation to the metabolic syndrome in young children | Ko BJ | 2014 | not included | Studying on blood selenium rather than selenium supplementation |
| Interplay between oxidative stress and metabolic derangements in non-alcoholic fatty liver disease: The role of selenoprotein p | Caviglia GP | 2020 | not included | Studying on blood selenium rather than selenium supplementation |
| Higher plasma selenium concentrations are associated with increased odds of prevalent type 2 diabetes | Kohler LN | 2018 | not included | Studying on blood selenium rather than selenium supplementation |
| High serum selenium levels are associated with increased risk for diabetes mellitus independent of central obesity and insulin resistance | Lu CW | 2016 | not included | Studying on blood selenium rather than selenium supplementation |
| First trimester microelements and their relationships with pregnancy outcomes and complications | Lewandowska M | 2020 | not included | Studying on blood selenium rather than selenium supplementation |
| Evaluation of serum selenium levels in turkish women with gestational diabetes mellitus, glucose intolerants, and normal controls | Kilinc M | 2008 | not included | Studying on blood selenium rather than selenium supplementation |
| Evaluation of endothelial dysfunction in patients with nonalcoholic fatty liver disease: Association of selenoprotein p with carotid intima-media thickness and endothelium-dependent vasodilation | Cetinda?l? I | 2017 | not included | Studying on blood selenium rather than selenium supplementation |
| Circulating selenoprotein p levels in relation to mri-derived body fat volumes, liver fat content, and metabolic disorders | R D | 2017 | not included | Studying on blood selenium rather than selenium supplementation |
| Changes of serum selenium in pregnant women with gestational diabetes mellitus | Tan M | 2001 | not included | Studying on blood selenium rather than selenium supplementation |
| Associations of multiple plasma metals with incident type 2 diabetes in chinese adults: The dongfeng-tongji cohort | Yuan Y | 2018 | not included | Studying on blood selenium rather than selenium supplementation |
| Associations of serum selenium levels in the first trimester of pregnancy with the risk of gestational diabetes mellitus and preterm birth: A preliminary cohort study | Liu PJ | 2021 | not included | Studying on blood selenium rather than selenium supplementation |
| Association of expression of selenoprotein p in mrna and protein levels with metabolic syndrome in subjects with cardiovascular disease: Results of the selenegene study | Gharipour M | 2017 | not included | Studying on blood selenium rather than selenium supplementation |
| Association of dietary and serum selenium concentrations with glucose level and risk of diabetes mellitus: A cross sectional study of national health and nutrition examination survey | Lin J | 2021 | not included | Studying on blood selenium rather than selenium supplementation |
| Association between serum selenium level and the prevalence of diabetes mellitus in u.s. Population | Moon S | 2019 | not included | Studying on blood selenium rather than selenium supplementation |
| Association between serum selenium level and the presence of diabetes mellitus: A meta-analysis of observational studies | Kim J | 2019 | not included | Studying on blood selenium rather than selenium supplementation |
| Association between plasma metal levels and diabetes risk: A case-control study in china | Li XT | 2017 | not included | Studying on blood selenium rather than selenium supplementation |
| Alterations of serum trace elements in patients with type 2 diabetes | Zhang H | 2017 | not included | Studying on blood selenium rather than selenium supplementation |
| A gene-environment interaction analysis of plasma selenium with prevalent and incident diabetes: The hortega study | Galan-chilet I | 2017 | not included | Studying on blood selenium rather than selenium supplementation |
| Selenium in intensive care (sic): Results of a prospective randomized, placebo-controlled, multiple-center study in patients with severe systemic inflammatory response syndrome, sepsis, and septic shock | Angstwurm M | 2007 | not included | Studying on blood selenium rather than selenium supplementation |
| Effect of selenium supplementation on adiponectin concentration as a marker of type-2 diabetes risk in uk pregnant women | J. MAO | 2015 | not included | Studying on blood selenium rather than selenium supplementation |
| Effects of selenium supplementation on glucose homeostasis and free androgen index in women with polycystic ovary syndrome: A randomized, double blinded, placebo controlled clinical trial | Hosseinzadeh F | 2016 | not included | Studying on blood selenium rather than selenium supplementation |
| Proposal of a study protocol of a preliminary double-blind randomized controlled trial. Verifying effects of selenium supplementation on selenoprotein p and s genes expression in protein and mrna levels in subjects with coronary artery disease: Selenegene study | Gharpour M | 2019 | not included | Studying on blood selenium rather than selenium supplementation |
| Results of a randomised trial of selenium supplementation and type­2 diabetes risk, as assessed by plasma adiponectin | Rayman M | 2020 | not included | Studying on blood selenium rather than selenium supplementation |
| Selenium supplementation affects insulin resistance and serum hs-crp in patients with type 2 diabetes and coronary heart disease | Farrokhian A | 2016 | not included | Studying on blood selenium rather than selenium supplementation |
| Supplementation with selenium-enriched yeast but not selenomethionine reduces oxidative stress in healthy men in a randomized clinical trial | Kaag M | 2014 | not included | Studying on blood selenium rather than selenium supplementation |
| The effect of selenium supplementation in the prevention of dna damage in white blood cells of hemodialyzed patients: A pilot study | Wasowicz B | 2010 | not included | Studying on blood selenium rather than selenium supplementation |
| Association between plasma selenium and glutathione peroxidase levels and severity of diabetic nephropathy in patients with type two diabetes mellitus | Sedighi O | 2014 | not included | Studying on blood selenium rather than selenium supplementation |
| High levels of plasma selenium are associated with metabolic syndrome and elevated fasting plasma glucose in a chinese population: A case-control study | Yuan, ZP | 2015 | not included | Studying on blood selenium rather than selenium supplementation |
| The relationship between selenoprotein p and glucose metabolism in experimental studies | Mao, JY | 2013 | not included | Studying on blood selenium rather than selenium supplementation |
| Correlation between selenoprotein p and insulin resistance in type 2 diabetes mellitus patients | Panjie M | 2014 | not included | Studying on blood selenium rather than selenium supplementation |
| Research progress on the relationship between selenoprotein p and type 2 diabetes mellitus and insulin resistance | Wang S | 2015 | not included | Studying on blood selenium rather than selenium supplementation |
| Selenoprotein s and type 2 diabetes | Yu BJ | 2020 | not included | Studying on blood selenium rather than selenium supplementation |
| Selenoproteins and diabetes: The dual nature of selenium | Zhou J | 2013 | not included | Studying on blood selenium rather than selenium supplementation |
| Circulating glutathione peroxidase-3 in elderly-association with renal function, cardiovascular mortality, and impact of selenium and coenzyme q(10) supplementation | Alexander J | 2024 | not included | Studying on blood selenium rather than selenium supplementation |
| Selenoprotein p increases upon selenium and coenzyme q(10) supplementation and is associated with telomere length, quality of life and reduced inflammation and mortality | Alehagen U | 2024 | not included | Studying on blood selenium rather than selenium supplementation |
| Selenoprotein p in myocardial infarction with cardiogenic shock | Büttner P | 2020 | not included | Studying on blood selenium rather than selenium supplementation |
| Decreased thyroid peroxidase antibody titer in response to selenium supplementation in autoimmune thyroiditis and the influence of a selenoprotein p gene polymorphism: A prospective, multicenter study in china | Wang W | 2018 | not included | Studying on blood selenium rather than selenium supplementation |
| Effects of selenium supplementation on expression of sepp1 in mrna and protein levels in subjects with and without metabolic syndrome suffering from coronary artery disease: Selenegene study a double-blind randomized controlled trial | Gharipour M | 2018 | not included | Studying on blood selenium rather than selenium supplementation |
| Selenium status in u.k. Pregnant women and its relationship with hypertensive conditions of pregnancy | Rayman MP | 2015 | not included | Studying on blood selenium rather than selenium supplementation |
| Selenoprotein and antioxidant genes and the risk of high-grade prostate cancer and prostate cancer recurrence | Gerstenberger JP | 2015 | not included | Studying on blood selenium rather than selenium supplementation |
| Vitamin d supplementation and serum levels of magnesium and selenium in type 2 diabetes mellitus patients: Gender dimorphic changes | Al-daghri NM | 2014 | not included | Studying on blood selenium rather than selenium supplementation |
| Influence of serum selenium concentrations on hypertension: The lipid analytic cologne cross-sectional study | Berthold HK | 2012 | not included | Studying on blood selenium rather than selenium supplementation |
| Serum selenium and plasma malondialdehyde levels and antioxidant enzyme activities in patients with obsessive-compulsive disorder | Ozdemir E | 2009 | not included | Studying on blood selenium rather than selenium supplementation |
| Effects of high doses of selenium, as sodium selenite, in septic shock: A placebo-controlled, randomized, double-blind, phase ii study | Forceville X | 2007 | not included | Studying on blood selenium rather than selenium supplementation |
| Elevated plasma glutathione peroxidase concentration in acute severe asthma: Comparison with plasma glutathione peroxidase activity, selenium and malondialdehyde | Jacobson GA | 2007 | not included | Studying on blood selenium rather than selenium supplementation |
| Effect of selenium supplementation on biochemical markers and outcome in critically ill patients | Mishra V | 2007 | not included | Studying on blood selenium rather than selenium supplementation |
| Plasma glutathione peroxidase by elisa and relationship to selenium level | Jacobson GA | 2006 | not included | Studying on blood selenium rather than selenium supplementation |
| Changes in serum proteomic patterns by presurgical alpha-tocopherol and l-selenomethionine supplementation in prostate cancer | Kim J | 2005 | not included | Studying on blood selenium rather than selenium supplementation |
| Centralized blood processing for the selenium and vitamin e cancer prevention trial: Effects of delayed processing on carotenoids, tocopherols, insulin-like growth factor-i, insulin-like growth factor binding protein 3, steroid hormones, and lymphocyte viability | Kristal AR | 2005 | not included | Studying on blood selenium rather than selenium supplementation |
| Supplementation with antioxidants in the treatment of graves' disease; the effect on glutathione peroxidase activity and concentration of selenium | Vrca VB | 2004 | not included | Studying on blood selenium rather than selenium supplementation |
| Selenium supplementation enhances low selenium levels and stimulates glutathione peroxidase activity in peripheral blood and distal colon mucosa in past and present carriers of colon adenomas | Al-taie OH | 2003 | not included | Studying on blood selenium rather than selenium supplementation |
| Alterations of serum selenium, zinc, copper, and iron concentrations and some related antioxidant enzyme activities in patients with cutaneous leishmaniasis | Kocyigit A | 1998 | not included | Studying on blood selenium rather than selenium supplementation |
| Selenium and training effects on the glutathione system and aerobic performance | Tessier F | 1995 | not included | Studying on blood selenium rather than selenium supplementation |
| Muscle gsh-px activity after prolonged exercise, training, and selenium supplementation | Tessier F | 1995 | not included | Studying on blood selenium rather than selenium supplementation |
| Serum selenium, glutathione peroxidase, lipids, and human liver microsomal enzyme activity : A double-blind controlled trial of selenium supplementation | Luoma PV | 1985 | not included | Studying on blood selenium rather than selenium supplementation |
| Selenium and risk factors for cardiovascular disease in new zealand | Robinson MF | 1983 | not included | Studying on blood selenium rather than selenium supplementation |
| Effect of selenium supplementation on oxidative stress in patient after cardiac arrest | Janotka M | 2018 | not included | Studying on blood selenium rather than selenium supplementation |
| Effects of selenium nanoparticles and sodium selenite on apoptosis and reactive oxygen species in chondrocytes from articular cartilage in patients with kashin-beck disease | Yao X | 2015 | not included | Studying on blood selenium rather than selenium supplementation |
| Low selenium status of new zealand residents | Robinson M | 1985 | not included | Studying on blood selenium rather than selenium supplementation |
| Prospective, randomized multicenter trial of adjunctive intravenous therapy with sodium-selenite(selenase?, double-blinded) and a procalcitonin guided causal therapy (open) of severe sepsis or septic shock | No author | 2009 | not included | Studying on blood selenium rather than selenium supplementation |
| Selenium supplementation in the modificátion of neurological outcome and antioxidants in acute ischemic stroke | Ramezani M | 2020 | not included | Studying on blood selenium rather than selenium supplementation |
| Selenoprotein p increases upon selenium and coenzyme q10 supplementation and is associated with telomere length, quality of life and reduced inflammation and mortality | Alehagen U | 2024 | not included | Studying on blood selenium rather than selenium supplementation |
| Strong associations of serum selenoprotein p with all-cause mortality and mortality due to cancer, cardiovascular, respiratory and gastrointestinal diseases in older german adults | Sch?ttker, B | 2024 | not included | Studying on blood selenium rather than selenium supplementation |
| Serum selenium and glutathione peroxidase-3 activity: Biomarkers of systemic inflammation in the critically ill? | Manzanares, W | 2009 | not included | Studying on blood selenium rather than selenium supplementation |
| Interaction of peroxynitrite with selenoproteins and glutathione peroxidase mimics | Sies, H | 2000 | not included | Studying on blood selenium rather than selenium supplementation |
| Antioxidant actions of selenium in orbital fibroblasts: A basis for the effects of selenium in graves' orbitopathy | Dottore, GR | 2017 | not included | Studying on blood selenium rather than selenium supplementation |
| The effects of selenium and the gpx-1 selenoprotein on the phosphorylation of h2ax | Jerome-morais, A | 2013 | not included | Studying on blood selenium rather than selenium supplementation |
| Plasma selenium levels and risk of new-onset diabetes in hypertensive adults | Zhang, YY | 2019 | not included | Studying on blood selenium rather than selenium supplementation |
| Serum selenium, selenoprotein p and glutathione peroxidase 3 in rheumatoid, psoriatic, juvenile idiopathic arthritis, and osteoarthritis | Wahl, L | 2025 | not included | Studying on blood selenium rather than selenium supplementation |
| Selenium status associates with thyroid hormone and thyroid dysfunction in older chinese adults | Li, F | 2025 | not included | Studying on blood selenium rather than selenium supplementation |
| Selenium and the selenoprotein thioredoxin reductase in the prevention, treatment and diagnostics of cancer | Selenius M | 2010 | not included | Studying on blood selenium rather than selenium supplementation |
| Study on gestational diabetes mellitus in relation to serum selenium levels and glutathione peroxidase activity | Pengfang L | 2015 | not included | Studying on blood selenium rather than selenium supplementation |
| Preliminary investigation into the relationship between serum selenium levels and metabolic factors associated with diabetes | Xue C | 2017 | not included | Studying on blood selenium rather than selenium supplementation |
| Clinical analysis of serum selenoprotein p changes in patients with gestational diabetes mellitus | Jiang L | 2019 | not included | Studying on blood selenium rather than selenium supplementation |
| A study on the association between plasma thrombosis and pre-diabetes risk | Luo C | 2019 | not included | Studying on blood selenium rather than selenium supplementation |

*Table footnote:* This table lists the articles assessed for eligibility but excluded, along with specific reasons for exclusion.

**Additional file 3: Table S3** Exploratory within-arm pre-post synthesis subject to regression to the mean. Comparison of glycemic, antioxidant, lipid, and anthropometric indicators.

| Indicators | Results for the control group and the intervention group | |  | Results before and after selenium supplementation in the intervention group | |
| --- | --- | --- | --- | --- | --- |
|  | WMD (95% CI) | Change^1^ |  | WMD (95% CI) | Change^1^ |
| FPG (mg/ dL ) | -2.15 (-6.43,2.14) | - |  | -4.62 (-9.09,-0.15) | ↓ |
| HbA1c(%) | -0.21 (-0.63,0.20) | - |  | -0.55 (-0.93,-0.17) | ↓ |
| FINS(μIU/ mL ) | -1.47 (-2.37,-0.57) | ↓ |  | -0.96 (-1.45,-0.48) | ↓ |
| HOMA-IR | -0.58 (-0.84,-0.31) | ↓ |  | -0.62 (-0.96,-0.28) | ↓ |
| HOMA-β | -17.57 (-23.91,-11.23) | ↓ |  | -9.88 (-16.45,-3.31) | ↓ |
| QUICKI | 0.010 (0.006,0.015) | ↑ |  | 0.01 (0.00,0.01) | ↑ |
| HDL-C (mg/ dL ) | 2.63 (0.01,5.26) | ↑ |  | 1.22 (0.15,2.29) | ↑ |
| LDL-C (mg/ dL ) | -4.27 (-8.20,-0.34) | ↓ |  | -4.95 (-9.62,-0.28) | ↓ |
| VLDL-C (mg/ dL ) | -0.55 (-1.65.0.54) | - |  | -0.86 (-2.21,0.49) | - |
| TG(mg/ dL ) | -5.93 (-10.18,-1.69) | ↓ |  | -4.57 (-13.81,4.67) | - |
| TC(mg/ dL ) | -4.14 (-7.40,-0.87) | ↓ |  | -5.35 (-9.91,-0.79) | ↓ |
| GSH(µmol/L) | 55.87 (31.58,80.17) | ↑ |  | 42.46 (18.10,66.82) | ↑ |
| TAC(mmol/L) | 23.76 (8.80,38.72) | ↑ |  | 21.66 (5.98,37.34) | ↑ |
| MDA(µmol/L) | -1.08 (-1.62,-0.54) | ↓ |  | -0.88 (-1.40,-0.36) | ↓ |
| NO(µmol/L) | 0.53 (-1.32,2.38) | - |  | 0.51 (-1.09,2.12) | - |
| SOD(U/ml) | 15.48 (5.77,25.19) | ↑ |  | 19.33 (12.13,26.54) | ↑ |
| GSH-Px(U/L) | 32.37 (7.81,56.93) | ↑ |  | 10.09 (6.20,13.97) | ↑ |
| hs-CRP(ng/ml) | -984.87 (-1419.65,-550.09) | ↓ |  | -1018.63 (-1441.02,-596.24) | ↓ |
| Se(µg/L) | 53.56 (34.33,72.79) | ↑ |  | 51.39 (32.42,70.36) | ↑ |
| Weight(kg) | 0.03 (-0.11,0.16) | - |  | 0.95 (-0.52,2.43) | - |
| BMI(kg/m2) | -0.00 (-0.05,0.05) | - |  | 0.32 (-0.19,0.83) | - |
| SBP(mmHg) | 1.04 (-0.01,2.10) | - |  | 2.93 (2.02,3.85) | ↑ |
| DBP(mmHg) | -1.56 (-2.30,-0.81) | ↓ |  | 0.84 (0.21,1.47) | ↑ |
| WC(cm) | 0.76 (-0.23,1.75) | - |  | -3.02 (-5.89,-0.15) | ↓ |

*Table footnote:* ^1^Change symbols indicate the direction of change after selenium supplementation: ↑ increase; ↔ no significant change; ↓ decrease.

**Additional file 4: Table S4** Subgroup analyses for metabolic and antioxidant outcomes.

| Subgroups | HOMA-β | | | |  | QUICKI | | | |  | SOD (U/mL) | | | |  | GSH-Px (U/L) | | | |
| --- | --- | --- | --- | --- | --- | --- | --- | --- | --- | --- | --- | --- | --- | --- | --- | --- | --- | --- | --- |
|  | n | WMD (95% CI) | *P*^1^ | I^2^ (%)^2^ |  | n | WMD (95% CI) | *P*^1^ | I^2^ (%)^2^ |  | n | WMD (95% CI) | *P*^1^ | I^2^ (%)^2^ |  | n | WMD (95% CI) | *P*^1^ | I^2^ (%)^2^ |
| Population |  |  |  |  |  |  |  |  |  |  |  |  |  |  |  |  |  |  |  |
| GDM/T2DM | 3 | -21.00 (-32.14,-9.86) | <0.001 | 0.0 |  | 3 | 0.015 (0.007,0.022) | <0.001 | 45.6 |  | - | - | - | - |  | - | - | - | - |
| Others | 2 | -15.93 (-23.64,-8.22) | <0.001 | 0.0 |  | 8 | 0.009 (0.003,0.014) | 0.002 | 68.9 |  | 6 | 15.48 (5.77,25.19) | 0.002 | 94.7 |  | 4 | 32.37 (7.81,56.93) | 0.010 | 97.3 |
| Country |  |  |  |  |  |  |  |  |  |  |  |  |  |  |  |  |  |  |  |
| Iran | 5 | -17.57 (-23.91,-11.23) | <0.001 | 5 |  | 11 | 0.010 (0.006,0.015) | <0.001 | 67.6 |  | - | - | - | - |  | - | - | - | - |
| Other countries | - | - | - | 0.0 |  | - | - | - | - |  | 6 | 15.48 (5.77,25.19) | 0.002 | 94.7 |  | 4 | 32.37 (7.81,56.93) | 0.010 | 97.3 |
| Region |  |  |  |  |  |  |  |  |  |  |  |  |  |  |  |  |  |  |  |
| Asia | 5 | -17.57 (-23.91,-11.23) | <0.001 | 0.0 |  | 11 | 0.012 (0.007,0.016) | <0.001 | 67.6 |  | 5 | 15.94 (5.86,26.02) | 0.002 | 95.7 |  | 3 | 35.23 (7.00,63.45) | 0.014 | 98.2 |
| Non-Asia | - | - | - | - |  | - | - | - | - |  | 1 | 8.00 (-26.07,42.07) | 0.645 | 0.0 |  | 1 | 21.70 (-2.90,46.30) | 0.084 | 0.0 |
| Duration |  |  |  |  |  |  |  |  |  |  |  |  |  |  |  |  |  |  |  |
| ≤84(day) | 5 | -17.57 (-23.91,-11.23) | <0.001 | 0.0 |  | 10 | 0.012 (0.007,0.0016) | <0.001 | 53.9 |  | 1 | 8.60 (-0.87,18.07) | 0.075 | 0.0 |  | 1 | 5.60 (-1.32,12.52) | 0.113 | 0.0 |
| >84(day) | - | - | - | - |  | 1 | <0.001 (-0.005,0.005) | 1.000 | 0.0 |  | 5 | 16.87 (5.79,27.95) | 0.003 | 95.7 |  | 3 | 47.29 (39.00,55.58) | <0.001 | 59.9 |
| Dosage |  |  |  |  |  |  |  |  |  |  |  |  |  |  |  |  |  |  |  |
| 200(µg/d) | 4 | -17.72 (-24.58,-10.86) | <0.001 | 0.0 |  | 10 | 0.011 (0.006,0.015) | <0.001 | 70.8 |  | 4 | 17.58 (5.97,29.19) | 0.003 | 96.8 |  | - | - | - | - |
| Others | 1 | -16.70 (-33.33,-0.07) | 0.049 | 0.0 |  | 1 | 0.010 (-<0.001,0.020) | 0.053 | 0.0 |  | 2 | 8.56 (-0.57,17.68) | 0.066 | 0.0 |  | 4 | 32.37 (7.81,56.93) | 0.010 | 97.3 |

| Subgroups | hs-CRP (ng/mL) | | | |  | Se (µg/L) | | | |  | HDL-C (mg/dL) | | | |  | LDL-C (mg/dL) | | | |
| --- | --- | --- | --- | --- | --- | --- | --- | --- | --- | --- | --- | --- | --- | --- | --- | --- | --- | --- | --- |
|  | n | WMD (95% CI) | *P*^1^ | I^2^ (%)^2^ |  | n | WMD (95% CI) | *P*^1^ | I^2^ (%)^2^ |  | n | WMD (95% CI) | *P*^1^ | I^2^ (%)^2^ |  | n | WMD (95% CI) | *P*^1^ | I^2^ (%)^2^ |
| Population |  |  |  |  |  |  |  |  |  |  |  |  |  |  |  |  |  |  |  |
| GDM/T2DM | 2 | -1281.01 (-2022.40,-539.61) | 0.001 | 0.0 |  | 3 | 46.02 (-9.07,101.11) | 0.102 | 99.6 |  | 5 | 1.00 (-0.74,2.74) | 0.261 | 0.0 |  | 5 | -6.61 (-13.50,0.28) ( | 0.060 | 52.8 |
| Others | 8 | -922.32 (-1402.99,-441.64) | <0.001 | 82.0 |  | 12 | 55.59 (33.91,77.27) | <0.001 | 98.5 |  | 15 | 2.91 (-0.42,6.24) | 0.087 | 96.4 |  | 13 | -3.15 (-7.91,1.62) ( | 0.196 | 47.4 |
| Country |  |  |  |  |  |  |  |  |  |  |  |  |  |  |  |  |  |  |  |
| Iran | 2 | -1281.01 (-2022.40,-539.61) | 0.001 | 0.0 |  | 3 | 46.02 (-9.07,101.11) | 0.102 | 99.6 |  | 5 | 1.00 (-0.74,2.74) | 0.261 | 0.0 |  | 5 | -8.59 (-15.30,-1.88) ( | 0.012 | 52.8 |
| Other countries | 8 | -922.32 (-1402.99,-441.64) | <<<0.001 | 82.0 |  | 12 | 55.59 (33.91,77.27) | <0.001 | 98.5 |  | 15 | 2.91 (-0.42,6.24) | 0.087 | 96.4 |  | 13 | -3.66 (-7.98,0.67) ( | 0.098 | 47.4 |
| Region |  |  |  |  |  |  |  |  |  |  |  |  |  |  |  |  |  |  |  |
| Asia | 10 | -984.87 (-1419.65,-550.09) | <0.001 | 79.2 |  | 8 | 34.23 (4.38,64.09) | 0.025 | 98.7 |  | 20 | 2.63 (0.01,5.26) | 0.049 | 95.2 |  | 18 | -4.27 (-8.20,-0.34) ( | 0.033 | 49.7 |
| Non-Asia | - | - | - | - |  | 7 | 72.09 (42.88,101.29) | <0.001 | 99.1 |  | - | - | - | - |  | - | - | - | - |
| Duration |  |  |  |  |  |  |  |  |  |  |  |  |  |  |  |  |  |  |  |
| ≤84(day) | 9 | -1148.57 (-1739.25,-557.90) | <0.001 | 80.0 |  | 3 | 15.10 (8.24,21.96) | <0.001 | 50.1 |  | 18 | 2.46 (-1.09,6.00) | 0.175 | 95.5 |  | 16 | -3.87 (-8.13,0.39) ( | 0.075 | 48.7 |
| >84(day) | 1 | -700.00 (-944.34,-455.66) | <0.001 | 0.0 |  | 12 | 64.96 (43.92,86.00) | <0.001 | 98.7 |  | 2 | 2.68 (0.68.4.68) | 0.009 | 17.1 |  | 2 | -6.71 (-17.61,4.20) ( | 0.228 | 58.6 |
| Dosage |  |  |  |  |  |  |  |  |  |  |  |  |  |  |  |  |  |  |  |
| 200(µg/d) | 8 | -913.59 (-1183.44,-643.74) | <0.001 | 20.8 |  | 5 | 47.87 (7.80,87.95) | 0.019 | 98.2 |  | 18 | 2.58 (-0.20,5.37) | 0.069 | 95.6 |  | 16 | -4.24 (-8.64,0.17) ( | 0.059 | 55.4 |
| Others | 2 | -1828.61 (-5548.07,1890.85) | 0.335 | 91.1 |  | 10 | 56.42 (32.47,80.37) | <0.001 | 99.1 |  | 2 | 3.16 (-3.88,10.20) | 0.379 | 67.8 |  | 2 | -4.95 (-13.70,3.80) | 0.267 | 0.0 |

| Subgroups | VLDL-C (mg/L) | | | |  | TG (mg/dL) | | | |  | TC (mg/dL) | | | |  | Weight (kg) | | | |
| --- | --- | --- | --- | --- | --- | --- | --- | --- | --- | --- | --- | --- | --- | --- | --- | --- | --- | --- | --- |
|  | n | WMD (95%CI) | *P*^1^ | I^2^ (%)^2^ |  | n | WMD (95%CI) | *P*^1^ | I^2^ (%)^2^ |  | n | WMD (95%CI) | *P*^1^ | I^2^ (%)^2^ |  | n | WMD (95%CI) | *P*^1^ | I^2^ (%)^2^ |
| Population |  |  |  |  |  |  |  |  |  |  |  |  |  |  |  |  |  |  |  |
| GDM/T2DM | 2 | -3.79(-6.77,-0.80) | 0.013 | 73.0 |  | 5 | -18.83(-28.06,-9.60) | <0.001 | 12.5 |  | 5 | -9.78(-15.86,-3.71) | 0.002 | 66.7 |  | 2 | 0.20(-0.30,0.69) | 0.432 | 0.0 |
| Others | 8 | -0.05(-1.23,1.13) | 0.931 | 0.0 |  | 16 | -2.47(-7.25,2.31) | 0.311 | 0.0 |  | 14 | -1.85(-5.72,2.02) | 0.348 | 0.0 |  | 10 | 0.01(-0.13,0.16) | 0.847 | 0.0 |
| Country |  |  |  |  |  |  |  |  |  |  |  |  |  |  |  |  |  |  |  |
| Iran | 10 | -0.55(-1.65,0.54) | 0.321 | 24.9 |  | 19 | -4.57(-9.05,-0.10) | 0.045 | 9.3 |  | 17 | -3.48(-6.92,-0.04) | 0.047 | 31.2 |  | 12 | 0.03(-0.11,0.16) | 0.688 | 0.0 |
| Other countries | - | - | - | - |  | 2 | -17.99(-31.34,-4.65) | 0.008 | 73.6 |  | 2 | -9.98(-20.25,0.29) | 0.057 | 0.0 |  | - | - | - | - |
| Region |  |  |  |  |  |  |  |  |  |  |  |  |  |  |  |  |  |  |  |
| Asia | 10 | -0.55(-1.65,0.54) | 0.321 | 24.9 |  | 21 | -5.93(-10.18,-1.69) | 0.006 | 26.3 |  | 19 | -4.14(-7.40,-0.87) | 0.013 | 29.2 |  | 12 | 0.03(-0.11,0.16) | 0.688 | 0.0 |
| Non-Asia | - | - | - | - |  | - | - | - | - |  | - | - | - | - |  | - | - | - | - |
| Duration |  |  |  |  |  |  |  |  |  |  |  |  |  |  |  |  |  |  |  |
| ≤84(day) | 9 | -1.33(-2.72,0.06) | 0.061 | 9.8 |  | 18 | -9.07(-13.99,-4.14) | <0.001 | 18.1 |  | 16 | -3.40(-7.05,0.26) | 0.069 | 37.2 |  | 10 | 0.04(-0.10,0.19) | 0.545 | 0.0 |
| >84(day) | 1 | 0.70(-1.07,2.47) | 0.439 | 0.0 |  | 3 | 3.18(-5.21,11.57) | 0.458 | 0.0 |  | 3 | -7.04(-14.28,0.20) | 0.057 | 0.0 |  | 2 | -0.10(-0.49,0.30) | 0.630 | 0.0 |
| Dosage |  |  |  |  |  |  |  |  |  |  |  |  |  |  |  |  |  |  |  |
| 200(µg/d) | 9 | -0.73(-1.92,0.46) | 0.230 | 30.1 |  | 18 | -7.38(-11.93,-2.83) | 0.001 | 28.0 |  | 16 | -4.14(-7.62,-0.66) | 0.020 | 40.0 |  | 10 | -0.02(-0.17,0.13) | 0.794 | 0.0 |
| Others | 1 | 0.40(-2.37,3.17) | 0.777 | 0.0 |  | 3 | 3.75(-8.02,15.52) | 0.532 | 0.0 |  | 3 | -4.09(-13.45,5.26) | 0.391 | 0.0 |  | 2 | 0.30(-0.05,0.66) | 0.095 | 0.0 |

| Subgroups | BMI (kg/m^2^) | | | |  | SBP (mmHg) | | | |  | DBP (mmHg) | | | |  | WC (cm) | | | |
| --- | --- | --- | --- | --- | --- | --- | --- | --- | --- | --- | --- | --- | --- | --- | --- | --- | --- | --- | --- |
|  | n | WMD (95%CI) | *P*^1^ | I^2^ (%)^2^ |  | n | WMD (95%CI) | *P*^1^ | I^2^ (%)^2^ |  | n | WMD (95%CI) | *P*^1^ | I^2^ (%)^2^ |  | n | WMD (95%CI) | *P*^1^ | I^2^ (%)^2^ |
| Population |  |  |  |  |  |  |  |  |  |  |  |  |  |  |  |  |  |  |  |
| GDM/T2DM | 2 | 0.04 (-0.12,0.20) | 0.633 | 0.0 |  | 2 | 0.79 (-0.33,1.90) | 0.167 | 0.0 |  | 2 | -1.82 (-2.61,-1.03) | <0.001 | 30.8 |  | - | - | - | - |
| Others | 12 | -0.01 (-0.06,0.05) | 0.806 | 0.0 |  | 6 | 3.18 (-0.04,6.41) | 0.091 | 0.0 |  | 6 | 0.50 (-1.71,2.72) | 0.655 | 0.0 |  | 3 | 0.76 (-0.23,1.75) | 0.133 | 0.0 |
| Country |  |  |  |  |  |  |  |  |  |  |  |  |  |  |  |  |  |  |  |
| Iran | 12 | -0.00 (-0.05,0.05) | 0.995 | 0.0 |  | 7 | 1.74 (-0.40,3.88) | 0.112 | 0.0 |  | 7 | 0.02 (-1.57,1.61) | 0.979 | 0.0 |  | 3 | 0.76 (-0.23,1.75) | 0.133 | 0.0 |
| Other countries | 2 | -0.05 (-0.30,0.21) | 0.703 | 0.0 |  | 1 | 0.82 (-0.39,2.03) | 0.184 | 0.0 |  | 1 | -2.00 (-2.84,-1.16) | <0.001 | 0.0 |  | - | - | - | - |
| Region |  |  |  |  |  |  |  |  |  |  |  |  |  |  |  |  |  |  |  |
| Asia | 13 | -0.00 (-0.05,0.05) | 0.993 | 0.0 |  | 7 | 1.74 (-0.40,3.88) | 0.112 | 0.0 |  | 7 | 0.02 (-1.57,1.61) | 0.979 | 0.0 |  | 3 | 0.76 (-0.23,1.75) | 0.133 | 0.0 |
| Non-Asia | 1 | -0.05 (-0.31,0.21) | 0.706 | 0.0 |  | 1 | 0.82 (-0.39,2.03) | 0.184 | 0.0 |  | 1 | -2.00 (-2.84,-1.16) | <0.001 | 0.0 |  | - | - | - | - |
| Duration |  |  |  |  |  |  |  |  |  |  |  |  |  |  |  |  |  |  |  |
| ≤84(day) | 13 | 0.01 (-0.04,0.07) | 0.627 | 0.0 |  | 8 | 1.04 (-0.01,2.10) | 0.053 | 0.0 |  | 8 | -1.06 (-2.24,0.12) | 0.078 | 15.1 |  | 3 | 0.76 (-0.23,1.75) | 0.133 | 0.0 |
| >84(day) | 1 | -0.10 (-0.24,0.04) | 0.155 | 0.0 |  | - | - | - | - |  | - | - | - | - |  | - | - | - | - |
| Dosage |  |  |  |  |  |  |  |  |  |  |  |  |  |  |  |  |  |  |  |
| 200(µg/d) | 11 | -0.01 (-0.07,0.04) | 0.627 | 0.0 |  | 7 | 1.74 (-0.40,3.88) | 0.112 | 0.0 |  | 7 | 0.02 (-1.57,1.61) | 0.979 | 0.0 |  | 3 | 0.76 (-0.23,1.75) | 0.133 | 0.0 |
| Others | 3 | 0.06 (-0.07,0.19) | 0.360 | 0.0 |  | 1 | 0.82 (-0.39,2.03) | 0.184 | 0.0 |  | 1 | -2.00 (-2.84,-1.16) | <0.001 | 0.0 |  | - | - | - | - |

*Table footnote:* ^1^*P* value for subgroup differences between groups. ^2^I² indicates the percentage of variability across studies due to heterogeneity.

**Additional file 5: Table S5** Exploratory within-arm pre-post synthesis subject to regression to the mean. Subgroup analyses by pre- and post-intervention status.

| **Subgroups** | **Se (µg/L)** | | | |  | **FPG (mg/dL)** | | | |  | **HbA1c (%)** | | | |  | **FINS (μIU/mL)** | | | |  | **HOMA-IR** | | | |  | **HOMA-β** | | | |  | **QUICKI** | | | | |
| --- | --- | --- | --- | --- | --- | --- | --- | --- | --- | --- | --- | --- | --- | --- | --- | --- | --- | --- | --- | --- | --- | --- | --- | --- | --- | --- | --- | --- | --- | --- | --- | --- | --- | --- | --- |
|  | **n** | **WMD (95% CI)** | **P^1^** | **I^2^(%)^2^** |  | **n** | **WMD(95%CI)** | **P^1^** | **I^2^(%)^2^** |  | **n** | **WMD(95%CI)** | **P^1^** | **I^2^(%)^2^** |  | **n** | **WMD(95%CI)** | **P^1^** | **I^2^(%)^2^** |  | **n** | **WMD(95%CI)** | **P^1^** | **I^2^(%)^2^** |  | **n** | **WMD(95%CI)** | **P^1^** | **I^2^(%)^2^** |  | **n** | **WMD(95%CI)** | **P^1^** | **I^2^(%)^2^** |  |
| Population |  |  |  |  |  |  |  |  |  |  |  |  |  |  |  |  |  |  |  |  |  |  |  |  |  |  |  |  |  |  |  |  |  |  |  |
| GDM/T2DM | 3 | 43.30 (-12.37,98.97) | 0.127 | 99.6 |  | 11 | -10.79 (-17.83,-3.75) | 0.042 | 97.3 |  | 5 | -0.78 (-1.18,-0.39) | <0.001 | 94.1 |  | 6 | -1.20 (-2.15,-0.25) | 0.014 | 0.0 |  | 6 | -0.73 (-1.47,0.01) | 0.054 | 89.0 |  | 2 | -4.22 (-15.16,6.73) | 0.450 | 0.0 |  | 3 | 0.01 (0.01,0.01) | <0.001 | 0.0 |  |
| Others | 12 | 53.47 (32.26,74.68) | <0.001 | 98.6 |  | 21 | -1.06 (-5.18,3.05) | 0.612 | 85.0 |  | 4 | -0.20 (-0.58,0.17) | 0.286 | 92.9 |  | 14 | -0.94 (-1.44,-0.44) | <0.001 | 42.8 |  | 15 | -0.47 (-0.71,-0.22) | <0.001 | 52.7 |  | 2 | -13.08 (-21.30,-4.85) | 0.002 | 55.9 |  | 10 | 0.01 (0.00,0.01) | 0.016 | 67.1 |  |
| Country |  |  |  |  |  |  |  |  |  |  |  |  |  |  |  |  |  |  |  |  |  |  |  |  |  |  |  |  |  |  |  |  |  |  |  |
| Iran | 3 | 19.39 (2.81,35.96) | 0.022 | 87.0 |  | 21 | -1.63 (-3.98,0.71) | 0.172 | 48.5 |  | 4 | -0.10 (-0.23,0.02) | 0.111 | 0.0 |  | 18 | -1.22 (-1.68,-0.76) | <0.001 | 0.0 |  | 19 | -0.47 (-0.67,-0.26) | <0.001 | 42.0 |  | 4 | -9.88 (-16.45, -3.30) | 0.003 | 27.6 |  | 13 | 0.01 (0.00,0.01) | 0.001 | 63.3 |  |
| Other countries | 12 | 59.79 (37.31,82.27) | <0.001 | 99.0 |  | 11 | -9.34 (-16.75,-1.93) | 0.014 | 97.8 |  | 5 | -0.78 (-1.20,-0.36) | <0.001 | 97.1 |  | 2 | 1.33 (-0.15,2.82) | 0.079 | 0.0 |  | 2 | -1.00 (-2.55,0.54) | 0.204 | 96.7 |  | - | - | - | - |  | - | - | - | - |  |
| Region |  |  |  |  |  |  |  |  |  |  |  |  |  |  |  |  |  |  |  |  |  |  |  |  |  |  |  |  |  |  |  |  |  |  |  |
| Asia | 8 | 30.99 (4.21,57.77) | 0.023 | 98.7 |  | 28 | -5.32 (-9.91,-0.73) | 0.023 | 95.2 |  | 8 | -0.51 (-0.90,-0.11) | 0.012 | 97.7 |  | 19 | -1.04 (-1.49,-0.59) | <0.001 | 24.6 |  | 21 | -0.60 (-0.92,-0.29) | <0.001 | 81.9 |  | 4 | -9.88 (-16.45, -3.30) | 0.003 | 27.6 |  | 13 | 0.01 (0.00,0.01) | 0.001 | 63.3 |  |
| Non-Asia | 7 | 72.76 (42.80,102.72) | <0.001 | 99.1 |  | 4 | 1.42 (-17.26,20.11) | 0.881 | 97.8 |  | 1 | -0.23 (-1.21,0.75) | 0.645 | 0.0 |  | 1 | 0.89 (-1.97,3.75) | 0.542 | 0.0 |  | - | - | - | - |  | - | - | - | - |  | - | - | - | - |  |
| Duration |  |  |  |  |  |  |  |  |  |  |  |  |  |  |  |  |  |  |  |  |  |  |  |  |  |  |  |  |  |  |  |  |  |  |  |
| ≤84(day) | 3 | 12.55 (5.61,19.49) | <0.001 | 52.2 |  | 24 | -5.04 (-9.01,-1.07) | 0.013 | 90.4 |  | 4 | -0.24 (-0.59,0.10) | 0.167 | 92.7 |  | 17 | -1.16 (-1.86,-0.46) | 0.001 | 32.8 |  | 19 | -0.60 (-0.92,-0.29) | <0.001 | 83.2 |  | 4 | -9.88 (-16.46,-3.31) | 0.003 | 27.6 |  | 12 | 0.01 (0.00,0.01) | <0.001 | 65.2 |  |
| >84(day) | 12 | 61.83 (41.02,82.64) | <0.001 | 98.8 |  | 8 | -2.41 (-14.68,9.86) | 0.700 | 98.2 |  | 5 | -0.79 (-1.15,-0.43) | <0.001 | 91.9 |  | 3 | -0.73 (-1.72,0.26) | 0.149 | 0.0 |  | 2 | -0.25 (-0.67,0.17) | 0.245 | 0.0 |  | - | - | - | - |  | 1 | 0.00 (-0.01,0.01) | 1.000 | 0.0 |  |
| Dosage |  |  |  |  |  |  |  |  |  |  |  |  |  |  |  |  |  |  |  |  |  |  |  |  |  |  |  |  |  |  |  |  |  |  |  |
| 200(µg/d) | 5 | 50.10 (10.45,89.74) | 0.013 | 98.2 |  | 22 | -4.50 (-11.54,2.55) | 0.211 | 95.8 |  | 5 | -0.35 (-0.99,0.30) | 0.290 | 97.5 |  | 17 | -1.22 (-1.69,-0.75) | <0.001 | 0.0 |  | 18 | -0.65 (-1.01,-0.29) | <0.001 | 84.1 |  | 3 | -11.39 (-19.26,-3.52) | 0.005 | 45.6 |  | 12 | 0.01 (0.00,0.01) | <0.001 | 59.4 |  |
| Others | 10 | 52.36 (29.16,75.56) | <0.001 | 99.1 |  | 10 | -4.39 (-10.03,1.26) | 0.128 | 95.4 |  | 4 | -0.67 (-1.25,-0.08) | 0.025 | 97.7 |  | 3 | 0.91 (-0.44,2.27) | 0.186 | 0.0 |  | 3 | -0.33 (-0.69,0.03) | 0.069 | 0.0 |  | 1 | -6.40 (-18.36,5.56) | 0.294 | 0.0 |  | 1 | 0.00 (-0.01,0.01) | 1.000 | 0.0 |  |

*Table footnote:* ^1^P value for subgroup differences between groups. ^2^I² indicates the percentage of variability across studies due to heterogeneity.

**Additional file 6: Table S6** Meta-analysis results for lipid and anthropometric outcomes.

| Indicators | Studies | I/C^1^ | WMD (95% CI) | *P*^2^ | I2 (%) | *P*^3^ | Model |  | Egger's Test | |  | Begg's Test | |  | **Quality of evidence** |
| --- | --- | --- | --- | --- | --- | --- | --- | --- | --- | --- | --- | --- | --- | --- | --- |
|  |  |  |  |  |  |  |  |  | t | *P* > \|t\| |  | z | *P*r > \|z\| |  |  |
| HDL-C(mg/dL) | 20 | 520/512 | 2.63 (0.01,5.26) | 0.049 | 95.2 | <0.001 | random |  | -1.18 | 0.269 |  | -1.79 | 0.073 |  | ⨁⨁◯◯ Low |
| LDL-C(mg/dL) | 18 | 486/478 | -4.27 (-8.20,-0.34) | 0.033 | 49.7 | 0.009 | random |  | -0.99 | 0.337 |  | -0.57 | 0.570 |  | ⨁⨁⨁◯Moderate |
| VLDL-C(mg/dL) | 10 | 265/267 | -0.55 (-1.65,0.54) | 0.321 | 24.9 | 0.214 | fixed |  | -1.18 | 0.258 |  | -1.44 | 0.151 |  | ⨁⨁⨁◯ Moderate |
| TG(mg/dL) | 21 | 560/550 | -5.93 (-10.18,-1.69) | 0.006 | 26.3 | 0.132 | fixed |  | 0.98 | 0.353 |  | 1.17 | 0.243 |  | ⨁⨁⨁⨁ High |
| TC(mg/dL) | 19 | 526/516 | -4.14 (-7.40,-0.87) | 0.013 | 29.2 | 0.114 | fixed |  | 0.73 | 0.505 |  | -0.19 | 0.851 |  | ⨁⨁⨁⨁ High |
| Weight(kg) | 12 | 330/330 | 0.03 (-0.11,0.16) | 0.688 | 0.0 | 0.928 | fixed |  | 0.17 | 0.869 |  | -0.27 | 0.784 |  | ⨁⨁⨁◯ Moderate |
| BMI(kg/m2) | 14 | 409/408 | -0.00 (-0.05,0.05) | 0.934 | 0.0 | 0.966 | fixed |  | 0.43 | 0.677 |  | -0.27 | 0.784 |  | ⨁⨁⨁⨁ High |
| SBP(mmHg) | 8 | 223/226 | 1.04 (-0.01,2.10) | 0.071 | 0.0 | 0.053 | fixed |  | NA | NA |  | NA | NA |  | ⨁⨁⨁◯Moderate |
| DBP(mmHg) | 8 | 223/226 | -1.56 (-2.30,-0.81) | <0.001 | 15.1 | 0.311 | fixed |  | NA | NA |  | NA | NA |  | ⨁⨁⨁◯ Moderate |
| WC(cm) | 3 | 70/70 | 0.76 (-0.23,1.75) | 0.133 | 0.0 | 0.725 | fixed |  | NA | NA |  | NA | NA |  | ⨁⨁◯◯Low |

*Table footnote:* ^1^I/C: I = number of patients in the intervention group; C = number in the control group. ^2^*P* value for subgroup differences between groups. ^3^*P* value for heterogeneity within each subgroup.

**Additional file 7: Table S7** Risk of bias assessment of the included randomized controlled trials using the Cochrane risk-of-bias tool.

| study | Random sequence generation  (selection bias) | Allocation concealment  (selection bias) | Blinding of participants and personnel  (performance bias) | Blinding of outcome assessment  (detection bias) | Incomplete outcome data  (attrition bias) | Selective reporting  (reporting bias) | Other bias |
| --- | --- | --- | --- | --- | --- | --- | --- |
| Yan XD 2021 China^[29]^ | Low | Unclear | Unclear | Unclear | Low | Low | Low |
| Meng XN 2022 China^[36]^ | Low | Unclear | Unclear | Unclear | Low | Low | Low |
| Li L 2023 China^[42]^ | Low | Unclear | Low | Unclear | Low | Low | Low |
| Bahmani 2016 Iran^[55]^ | Low | Low | Low | Low | Low | Low | Low |
| Amirani 2022 Iran^[37]^ | Low | Low | Low | Low | Low | Low | Low |
| Salimian 2022 Iran^[43]^ | Low | Low | Low | Low | Low | Low | Low |
| Asemi 2017 Iran^[40]^ | Low | Low | Low | Low | Low | Low | Low |
| Zadeh Modarres 2022 Iran^[45]^ | Low | Low | Low | Low | Low | Low | Low |
| Algotar 2013 United States^[24]^ | Low | Low | Low | Low | Low | Unclear | Low |
| Mahdiyeh 2021 Iran^[25]^ | Low | Low | Low | Low | Low | Unclear | Low |
| Najib FS 2020 Iran^[39]^ | Low | Low | Low | Low | Low | Unclear | Low |
| Mohammad Hosseinzadeh 2016 Iran^[47]^ | Low | Low | Low | Low | Low | Unclear | Low |
| Mesdaghinia 2016 Iran^[48]^ | Low | Low | Low | Low | Low | Low | Low |
| Faghihi T 2014 Iran^[31]^ | Low | Low | Low | Low | Low | Low | Low |
| Rayman MP 2012 United Kingdom^[28]^ | Low | Low | Low | Low | Low | Low | Low |
| Farrokhian 2016 Iran^[32]^ | Low | Low | Low | Low | Low | Low | Low |
| Zhang 2014 China^[33]^ | Low | Unclear | Unclear | Unclear | Low | Unclear | Unclear |
| Jia 2020 China^[34]^ | Low | Unclear | Unclear | Unclear | Low | Unclear | Unclear |
| Deng 2013 China^[56]^ | Low | Unclear | Unclear | Unclear | Low | Unclear | Unclear |
| Asemi 2015 Iran^[40]^ | Low | Low | Low | Low | Low | Unclear | Unclear |
| Saifi 2019 Algerian^[41]^ | Unclear | Unclear | Unclear | Unclear | Low | Low | Unclear |
| Chen 2014 China^[49]^ | Low | Unclear | Unclear | Unclear | Low | Unclear | Unclear |
| Bahmani 2015 Iran^[50]^ | Low | Unclear | Unclear | Unclear | Low | Unclear | Unclear |
| Jamilian 2015 Iran^[51]^ | Low | Low | Low | Low | Low | Low | Low |
| Raygan 2018 Iran^[44]^ | Low | Low | Unclear | Unclear | Low | Low | Low |
| Kamali 2019 Iran^[52]^ | Low | Low | Low | Low | Low | Low | Low |
| Gargari 2015 Iran^[26]^ | Unclear | Unclear | Unclear | Unclear | Low | Unclear | Unclear |
| Tamtaji OR 2019 Iran^[46]^ | Low | Low | Low | Low | Low | Low | Low |
| Rashidi BH 2020 Iran^[73]^ | Low | Low | Low | Low | Low | Low | Low |
| Zamani B 2024 Iran^[53]^ | Low | Low | Low | Low | Low | Low | Low |
| Faure, P. 2004 France^[35]^ | Low | Unclear | Unclear | Low | Low | Low | Low |
| Razavi, M 2016 Iran^[74]^ | Low | Low | Low | Low | Low | Low | Low |
| Mahmoodianfard, S 2015 Iran^[75]^ | Low | Low | Low | Low | Low | Low | Low |
| Balali A 2024 Iran^[61]^ | Low | Low | Low | Low | Low | Low | Low |
| Hu Y 2021 China^[76]^ | Low | Low | Unclear | Low | Low | Low | Low |
| Tian X 2020 China^[62]^ | Low | Low | Low | Low | Low | Low | Low |
| Adamowicz, A. 2002 Poland^[77]^ | Low | Low | Low | Low | Low | Low | Low |
| Atapour, A. 2022 Iran^[78]^ | Low | Low | Low | Low | Low | Low | Low |
| Wu XQ 2023 China^[79]^ | Low | Low | Low | Low | Low | Low | Low |
| Zhou JP 2022 China^[80]^ | Low | Low | Low | Low | Low | Low | Low |
| Qu CM 2019 China^[81]^ | Low | Unclear | Unclear | Unclear | Low | Low | Low |
| Zhao R 2000 China^[82]^ | Low | Unclear | Unclear | Unclear | Low | Low | Low |
| Wang B 2015 China^[54]^ | Low | Low | Low | Low | Low | Low | Low |
| Ye JJ 2018 China^[83]^ | Low | Unclear | Unclear | Unclear | Low | Low | Low |
| XU JL 2012 China^[84]^ | Low | Unclear | Unclear | Unclear | Low | Low | Low |
| Rui LX 1998 China^[27]^ | Low | Unclear | Unclear | Unclear | Low | Low | Low |

**Additional file 8: Table S8** GRADE summary of findings and quality of evidence.

| Outcomes | Certainty assessment | | | | | | | No. of patients | | Effect | | Quality of evidence |
| --- | --- | --- | --- | --- | --- | --- | --- | --- | --- | --- | --- | --- |
|  | No. of studies | Study design | Risk of bias | Inconsistency | Indirectness | Imprecision | Other considerations | Selenium supplementation | No selenium supplementation | Relative(95% CI) | Absolute (95% CI) |  |
| FPG | 30 | randomised trials | not serious ^1^ | very serious^a^ | not serious | serious^d^ | publication bias strongly suspected^b^ | 1328 | 1327 |  | MD 2.15 lower  (6.43 lower to 2.14 higher) | ⨁◯◯◯ Very low^a,b,d^ |
| HbA1c | 8 | randomised trials | not serious | very serious^c^ | not serious | serious^d^ | none | 295 | 293 | - | MD 0.21 lower (0.63 lower to 0.2 higher) | ⨁◯◯◯ Very low^c,d^ |
| FINS | 20 | randomised trials | not serious | serious^e^ | not serious | not serious | none | 506 | 511 | - | MD 1.47 lower  (2.37 lower to 0.57 lower) | ⨁⨁⨁◯ Moderate^e^ |
| HOMA-IR | 19 | randomised trials | not serious | very serious^f^ | not serious | not serious | none | 492 | 488 | - | MD 0.58 lower (0.84 lower to 0.31 lower) | ⨁⨁◯◯ Low^f^ |
| HOMA-β | 5 | randomised trials | not serious | not serious | not serious | very serious^d^ | none | 160 | 160 | - | MD 17.57 lower (23.91 lower to 11.23 lower) | ⨁⨁◯◯  Low^d^ |
| QUICKI | 11 | randomised trials | not serious | serious^g^ | not serious | serious^d^ | none | 300 | 302 | - | MD 0.01 higher (0.006 higher to 0.015 higher) | ⨁⨁◯◯ Low^d,g^ |
| GSH | 11 | randomised trials | not serious | not serious | not serious | serious^d^ | none | 296 | 300 | - | MD 55.87 higher (31.58 higher to 80.17 higher) | ⨁⨁⨁◯  Moderate^d^ |
| TAC | 13 | randomised trials | not serious | very serious^h^ | serious^i^ | serious^d^ | publication bias strongly suspected^j^ | 350 | 350 | - | MD 23.76 higher (8.8 higher to 38.72 higher) | ⨁◯◯◯ Very low^d,h,i,j^ |
| MDA | 15 | randomised trials | not serious | very serious^k^ | not serious | not serious | none | 463 | 462 | - | MD 1.08 lower (1.62 lower to 0.54 lower) | ⨁⨁◯◯ Low^k^ |
| NO | 11 | randomised trials | not serious | serious^l^ | serious^m^ | serious^d^ | none | 306 | 309 | - | MD 0.53 higher (1.32 lower to 2.38 higher) | ⨁◯◯◯ Very low^d,l,m^ |
| SOD | 6 | randomised trials | not serious | very serious^n^ | not serious | serious^d^ | none | 195 | 191 | - | MD 15.48 higher (5.77 higher to 25.19 higher) | ⨁◯◯◯ Very low^d,n^ |
| GSH-Px | 4 | randomised trials | not serious | very serious^o^ | not serious | very serious^p^ | none | 71 | 71 | - | MD 32.37 higher (7.81 higher to 56.93 higher) | ⨁◯◯◯ Very low^o,p^ |
| hs-CRP | 10 | randomised trials | not serious | very serious^q^ | not serious | serious^d^ | publication bias strongly suspected^j^ | 267 | 270 | - | MD 984.87 lower (1419.65 lower to 550.09 lower) | ⨁◯◯◯ Very low^d,j,q^ |
| Se | 15 | randomised trials | not serious | very serious^r^ | not serious | not serious | none | 1139 | 1105 | - | MD 53.56 higher (34.33 higher to | ⨁⨁◯◯ Low^r^ |
| HDL | 20 | randomised trials | not serious | very serious^s^ | not serious | not serious | none | 520 | 512 | - | MD 2.63 higher (0.01 higher to 5.26 higher) | ⨁⨁◯◯ Low^s^ |
| LDL | 18 | randomised trials | not serious | serious^t^ | not serious | not serious | none | 486 | 478 | - | MD 4.27 lower (8.2 lower to 0.34 lower) | ⨁⨁⨁◯ Moderate^t^ |
| VLDL | 10 | randomised trials | not serious | not serious | not serious | serious^d^ | none | 265 | 267 | - | MD 0.55 lower (1.65 lower to 0.54 higher) | ⨁⨁⨁◯  Moderate^d^ |
| TG | 21 | randomised trials | not serious | not serious | not serious | not serious | none | 560 | 550 | - | MD 5.93 lower (10.18 lower to 1.69 lower) | ⨁⨁⨁⨁  High |
| TC | 19 | randomised trials | not serious | not serious | not serious | not serious | none | 526 | 516 | - | MD 4.14 lower (7.4 lower to 0.87 lower) | ⨁⨁⨁⨁  High |
| Weight | 12 | randomised trials | not serious | not serious | not serious | seriousd | none | 330 | 330 | - | MD 0.03 higher (0.11 lower to 0.16 higher) | ⨁⨁⨁◯  Moderate^d^ |
| BMI | 14 | randomised trials | not serious | not serious | not serious | not serious | none | 409 | 408 | - | MD 0  (0.05 lower to 0.05 higher) | ⨁⨁⨁⨁  High |
| SBP | 8 | randomised trials | not serious | not serious | not serious | serious^d^ | none | 223 | 226 | - | MD 1.04 higher (0.01 lower to 2.1 higher) | ⨁⨁⨁◯ Moderate^d^ |
| DBP | 8 | randomised trials | not serious | not serious | not serious | serious^d^ | none | 223 | 226 | - | MD 1.56 lower (2.3 lower to 0.81 lower) | ⨁⨁⨁◯  Moderate^d^ |
| WC | 3 | randomised trials | not serious | not serious | not serious | very serious^d^ | none | 70 | 70 | - | MD 0.76 higher (0.23 lower to 1.75 higher) | ⨁⨁◯◯ Low^d^ |

*Table footnote:*

^a^ Downgraded by two levels for very serious inconsistency because substantial heterogeneity was observed for FPG (I² = 94.6%, *P* < 0.001), and subgroup analyses did not fully explain the between-study variability.

^b^ Publication bias was strongly suspected for FPG because funnel plot asymmetry was observed and Egger’s test suggested small-study effects.

^c^ Downgraded by two levels for very serious inconsistency because substantial heterogeneity was observed for HbA1c (I² = 95.3%, *P* < 0.001), and the direction and magnitude of effects varied across studies.

^d^ Downgraded for imprecision because the optimal information size was not met, the number of contributing studies or participants was limited, or the 95% confidence interval crossed the line of no effect or included both clinically important benefit and no effect.

^e^ Downgraded by one level for serious inconsistency because moderate heterogeneity was observed for FINS (I² = 63.3%, *P* < 0.001).

^f^ Downgraded by two levels for very serious inconsistency because substantial heterogeneity was observed for HOMA-IR (I² = 75.5%, *P* < 0.001).

^g^ Downgraded by one level for serious inconsistency because moderate heterogeneity was observed for QUICKI (I² = 67.6%, *P* < 0.001), and subgroup analyses did not fully explain the variability.

^h^ Downgraded by two levels for very serious inconsistency because high heterogeneity was observed for TAC (I² = 79.5%, *P* < 0.001).

^i^ Downgraded by one level for indirectness because subgroup analyses showed a significant difference between selenium-alone and balanced co-intervention trials for TAC, suggesting that intervention type may influence this outcome.

^j^ Publication bias was strongly suspected because funnel plot asymmetry was observed and Egger’s or Begg’s test suggested small-study effects. In this analysis, publication bias was strongly suspected for TAC and hs-CRP.

^k^ Downgraded by two levels for very serious inconsistency because very high heterogeneity was observed for MDA (I² = 96.4%, *P* < 0.001).

^l^ Downgraded by one level for serious inconsistency because high heterogeneity was observed for NO (I² = 70.2%, *P* < 0.001).

^m^ Downgraded by one level for indirectness because subgroup analyses showed a significant difference between selenium-alone and balanced co-intervention trials for NO, with inconsistent effect directions across intervention types.

^n^ Downgraded by two levels for very serious inconsistency because very high heterogeneity was observed for SOD (I² = 94.7%, *P* < 0.001).

^o^ Downgraded by two levels for very serious inconsistency because very high heterogeneity was observed for GSH-Px (I² = 97.3%, *P* < 0.001).

^p^ Downgraded by two levels for very serious imprecision because the evidence for GSH-Px was based on only 4 treatment arms and 142 participants, resulting in limited certainty around the pooled estimate.

^q^ Downgraded by two levels for very serious inconsistency because high heterogeneity was observed for hs-CRP (I² = 79.2%, *P* < 0.001).

^r^ Downgraded by two levels for very serious inconsistency because very high heterogeneity was observed for serum selenium (I² = 98.9%, *P* < 0.001).

^s^ Downgraded by two levels for very serious inconsistency because very high heterogeneity was observed for HDL-C (I² = 95.2%, *P* < 0.001).

^t^ Downgraded by one level for serious inconsistency because heterogeneity was suggested for LDL-C by Cochran’s Q test (*P* = 0.009), although the I² value was close to the conventional threshold for substantial heterogeneity.

Publication bias was evaluated by visual inspection of funnel plots and, when at least 10 studies were available, by Egger’s and Begg’s tests. Formal statistical tests for publication bias were not performed for outcomes with fewer than 10 contributing studies because of limited statistical power. Dose–response evidence was considered supportive but was not used to upgrade the certainty of evidence when important concerns remained regarding inconsistency, imprecision, indirectness, or potential publication bias.ublication bias was evaluated by visual inspect.

**Additional file 9: Table S9** Subgroup analysis by intervention context: selenium-alone trials versus balanced co-intervention trials.

| **Outcome** | **Selenium-alone trials** | **Balanced co-intervention trials** | ***P* for subgroup difference** |
| --- | --- | --- | --- |
| FPG(mg/dL) | -2.33(-5.82,1.16) | -3.71(-16.42,9.00) | 0.837 |
| HbA1c(%) | -0.12(-1.20,0.97) | -0.27(-0.56,0.02) | 0.786 |
| FINS(μIU/mL) | -1.91(-3.35,-0.48) | -1.04(-4.07,1.99) | 0.612 |
| HOMA-IR | -0.75(-1.17,-0.32) | -0.33(-0.97,0.32) | 0.288 |
| HOMA-B | -20.43(-28.74,-12.12) | -13.60(-23.40,-3.80) | 0.297 |
| QUICKI | 0.013(0.007,0.020) | 0.010(0.005,0.015) | 0.419 |
| HDL-C (mg/dL) | 3.05(-2.31,8.41) | -0.001(-0.203,0.202) | 0.265 |
| LDL-C (mg/dL) | -3.84(-8.34,0.67) | 2.21(-8.57,12.98) | 0.311 |
| VLDL-C (mg/dL) | -1.26(-3.05,0.52) | -2.90(-6.86,1.06) | 0.548 |
| TG (mg/dL) | -9.59(-15.00,-4.18) | -7.46(-24.97,10.04) | 0.820 |
| TC (mg/dL) | -5.14(-9.35,-0.93) | 3.60(-4.19,11.39) | 0.123 |
| GSH(µmol/L) | 40.14(1.74,78.54) | 50.50(-104.29,205.29) | 0.899 |
| TAC(mmol/L) | 83.73(18.7,148.76) | 6.72(-0.61,14.05) | 0.021 |
| MDA(µmol/L) | -1.09(-1.94,-0.25) | -1.31(-2.01,-0.62) | 0.692 |
| NO(µmol/L) | 1.75(-0.64,4.14) | -2.42(-3.88,-0.95) | 0.004 |
| SOD(U/mL) | 11.16(0.82,21.50) | 20.70(2.74,38.65) | 0.367 |
| GSH-Px(U/L) | 35.23(7,63.45) | 21.70(-2.9,46.3) | 0.479 |
| hs-CRP(ng/mL) | -1399.86(-2298.15,-501.58) | -933.90(-1763.22,-104.58) | 0.455 |
| Se(ug/L) | 51.28(30.33,72.22) | 68.75(-1.55,139.05) | 0.641 |
| Weight(kg) | 0.14(-0.11,0.39) | 0.000(-0.202,0.202) | 0.270 |
| BMI(kg/m^2^) | 0.07(-0.03,0.17) | -0.02(-0.10,0.05) | 0.158 |
| SBP(mmHg) | 1.57(-0.72,3.86) | 0.90(-0.29,2.09) | 0.610 |
| DBP(mmHg) | -0.35(-2.1,1.41) | -1.82(-2.65,-1.00) | 0.135 |
| WC(cm) | -0.28(-4.17,3.61) | 0.83(-0.19,1.85) | 0.589 |

*Table footnote:* Selenium-alone trials were defined as trials comparing selenium supplementation with placebo or no selenium. Balanced co-intervention trials were defined as trials in which both groups received the same active background intervention, and the only between-group difference was selenium supplementation. Trials with unbalanced co-interventions were not eligible for inclusion. *P* ≥ 0.05 indicates no statistically significant difference between the two groups; *P* < 0.05 indicates a statistically significant difference; *P* < 0.01 indicates a highly significant difference.

**Additional file10: Figure S1** Quality Evaluation plot. Quality evaluation plot showing risk of bias for each included study. Green indicates low risk of bias, red indicates high risk of bias, and yellow indicates unclear risk of bias.

**Additional file 11: Figure S2** Leave-one-out sensitivity analyses for the primary outcomes. Leave-one-out sensitivity analyses for all primary outcomes: **a** fasting plasma glucose (FPG), **b** fasting insulin (FINS), **c** HOMA-IR, **d** serum selenium (Se), **e** QUICKI, **f** hs-CRP, **g** HbA1c, **h** superoxide dismutase (SOD), **i** malondialdehyde (MDA), **j** total antioxidant capacity (TAC), **k** glutathione (GSH), **l** nitric oxide (NO), **m** HOMA-β, **n** glutathione peroxidase (GSH-Px).

**Additional file 12: Figure S3** Funnel plots assessing publication bias. Funnel plots for publication bias assessment across primary outcomes: **a** FPG, **b** HbA1c, **c** FINS, **d** QUICKI, **e** HOMA-β, **f** HOMA-IR, **g** serum selenium Se, **h** GSH, **i** MDA, **j** SOD, **k** TAC, **l** GSH-Px, **m** hs-CRP, **n** NO. For outcomes with fewer than 10 contributing studies, funnel plots are presented only for visual reference and were not formally interpreted using Egger’s or Begg’s tests.

**References**

1.	Manning A HH, Gasser J, et al.: **A Low-Frequency Inactivating AKT2 Variant Enriched in the Finnish Population Is Associated With Fasting Insulin Levels and Type 2 Diabetes Risk.** *Diabetes* 2017, **66:**2019-2032.doi: 10.2337/db16-1329.

2.	Ni C JQ, Ding G, Wu X, Yang M: **Low-Glycemic Index Diets as an Intervention in Metabolic Diseases: A Systematic Review and Meta-Analysis.** *Nutrients* 2022, **14**.doi: 10.3390/nu14020307.

3.	Francesco Giorgino AL, Luigi Laviola: **Cardiovascular disease and glycemic control in type 2 diabetes: now that the dust is settling from large clinical trials.** *Ann N Y Acad Sci* 2013, **1281:**36-50.doi: 10.1111/nyas.12044.

4.	Grundy SM: **Metabolic syndrome pandemic.** *Arterioscler Thromb Vasc Biol* 2008, **28:**629-636.doi: 10.1161/atvbaha.107.151092.

5.	Ismael SA: **Effects of low carbohydrate diet compared to low fat diet on reversing the metabolic syndrome, using NCEP ATP III criteria: a randomized clinical trial.** *BMC Nutr* 2021, **7:**62.10.1186/s40795-021-00466-8.

6.	Steinbrenner H, Duntas LH, Rayman MP: **The role of selenium in type-2 diabetes mellitus and its metabolic comorbidities.** *Redox Biol* 2022, **50:**102236.10.1016/j.redox.2022.102236.

7.	Pei J, Pan X, Wei G, Hua Y: **Research progress of glutathione peroxidase family (GPX) in redoxidation.** *Front Pharmacol* 2023, **14:**1147414.10.3389/fphar.2023.1147414.

8.	Lee JG, Jang JY, Baik SM: **Selenium as an Antioxidant: Roles and Clinical Applications in Critically Ill and Trauma Patients: A Narrative Review.** *Antioxidants (Basel)* 2025, **14**.10.3390/antiox14030294.

9.	Huang Z, Rose AH, Hoffmann PR: **The role of selenium in inflammation and immunity: from molecular mechanisms to therapeutic opportunities.** *Antioxid Redox Signal* 2012, **16:**705-743.10.1089/ars.2011.4145.

10.	Casanova P, Monleon D: **Role of selenium in type 2 diabetes, insulin resistance and insulin secretion.** *World J Diabetes* 2023, **14:**147-158.10.4239/wjd.v14.i3.147.

11.	Ouyang J, Cai Y, Song Y, Gao Z, Bai R, Wang A: **Potential Benefits of Selenium Supplementation in Reducing Insulin Resistance in Patients with Cardiometabolic Diseases: A Systematic Review and Meta-Analysis.** *Nutrients* 2022, **14**.10.3390/nu14224933.

12.	Shimada BK, Alfulaij N, Seale LA: **The Impact of Selenium Deficiency on Cardiovascular Function.** *Int J Mol Sci* 2021, **22**.10.3390/ijms221910713.

13.	Jablonska E, Reszka E, Gromadzinska J, Wieczorek E, Krol MB, Raimondi S, Socha K, Borawska MH, Wasowicz W: **The Effect of Selenium Supplementation on Glucose Homeostasis and the Expression of Genes Related to Glucose Metabolism.** *Nutrients* 2016, **8**.10.3390/nu8120772.

14.	Ma XM, Li KX, Chen ZQ, Wu CM, Liao WZ, Guo XG: **Impact of age, sex, and thyroid autoimmunity on the association between selenium intake and type 2 diabetes mellitus.** *BMC Public Health* 2024, **24:**743.10.1186/s12889-024-18225-2.

15.	Mahdavi Gorabi A, Hasani M, Djalalinia S, Zarei M, Ejtahed H, Abdar ME, Asayesh H, Azimzadeh M, Qorbani M, Noroozi M: **Effect of selenium supplementation on glycemic indices: a meta-analysis of randomized controlled trials.** *J Diabetes Metab Disord* 2019, **18:**349-362.10.1007/s40200-019-00419-w.

16.	Dean Follmann PE, Il Suh, Jeffrey Cutler,: **Variance imputation for overviews of clinical trials with continuous response.** *Journal of Clinical Epidemiology* 1992, **45:**769-773.doi: 10.1016/0895-4356(92)90054-q.

17.	Keith R Abrams CLG, Paul C Lambert: **Meta-analysis of heterogeneously reported trials assessing change from baseline.** *Stat Med* 2005, **24:**3823-3844.doi: 10.1002/sim.2423.

18.	Stela Pudar Hozo BD, Iztok Hozo: **Estimating the mean and variance from the median, range, and the size of a sample.** *BMC Med Res Methodol* 2005, **5:**13.doi: 10.1186/1471-2288-5-13.

19.	Simental-Mendía LE, Sahebkar A, Rodríguez-Morán M, Guerrero-Romero F: **A systematic review and meta-analysis of randomized controlled trials on the effects of magnesium supplementation on insulin sensitivity and glucose control.** *Pharmacol Res* 2016, **111:**272-282.10.1016/j.phrs.2016.06.019.

20.	SAR XCaD: **The robust error meta-regression method for dose-response meta-analysis.** *Int J Evid Based Healthc* 2018, **16:**138-144.doi: 10.1097/xeb.0000000000000132.

21.	Higgins JP, Altman DG, Gøtzsche PC, Jüni P, Moher D, Oxman AD, Savovic J, Schulz KF, Weeks L, Sterne JA: **The Cochrane Collaboration's tool for assessing risk of bias in randomised trials.** *Bmj* 2011, **343:**d5928.10.1136/bmj.d5928.

22.	Julian P T Higgins SGT, Douglas G Altman: **Measuring inconsistency in meta-analyses.** *Bmj* 2003, **327:**557-560.doi: 10.1136/bmj.327.7414.557.

23.	Andreas E Stuck C, Laurence Z Rubenstein, Professor of geriatric medicineb,Darryl Wieland, Professorc: **Bias in meta-analysis detected by a simple, graphical test.** *Bmj* 1998, **315:**629-634.doi: 10.1136/bmj.315.7109.629.

24.	Algotar AM: **Selenium supplementation has no effect on serum glucose levels in men at high risk of prostate cancer.** *J Diabetes* 2013, **5:**465-470.10.1111/1753-0407.12041.

25.	Mahdiyeh KKG: **Effects of sodium selenite and selenium-enriched yeast on cardiometabolic indices of patients with atherosclerosis: A double-blind randomized clinical trial study.** *J Cardiovasc Thorac Res* 2021, **13:**314-319.10.34172/jcvtr.2021.51.

26.	Gargari: **Effect of L-arginine and Selenium on Metabolic Features, Insulin Resistance and Hepatic Function Tests in Obese Women.** 2015

27.	Rui Lixin ZY, Mei Weide, et al: **The antagonistic effect of selenate polysaccharides on lipid peroxidation in cancer patients caused by chemotherapy.** *Studies of Trace Elements and Health* 1998, **15:**21-23

28.	Rayman MP: **A randomized trial of selenium supplementation and risk of type-2 diabetes, as assessed by plasma adiponectin.** *PLoS One* 2012, **7:**e45269.10.1371/journal.pone.0045269.

29.	XD Y: **The Effect of Selenium Yeast Tablets Combined with Levothyroxine Sodium on Blood Glucose Levels and Serum TPOAb and TGAb Levels in Patients with Type 2 Diabetes and Chronic Lymphocytic Thyroiditis.** *Chronic Pathematology J* 2021, **22:**814-816.10.16440/j.cnki.1674-8166.2021.05.057.

30.	Saverio Stranges JRM, Raj Natarajan, Richard P. Donahue, Maurizio Trevisan, Gerald F. Combs, Francesco P. Cappuccio, Antonio Ceriello, and Mary E. Reid: **Effects of Long-Term Selenium Supplementation on the Incidence of Type 2 Diabetes.** *Annals of Internal Medicine* 2007, **147:**217-223.doi: 10.7326/0003-4819-147-4-200708210-00175.

31.	T F: **A randomized, placebo-controlled trial of selenium supplementation in patients with type 2 diabetes: effects on glucose homeostasis, oxidative stress, and lipid profile.** 2014

32.	Farrokhian A: **Selenium Supplementation Affects Insulin Resistance and Serum hs-CRP in Patients with Type 2 Diabetes and Coronary Heart Disease.** *Horm Metab Res* 2016, **48:**263-268.10.1055/s-0035-1569276.

33.	Zhang Gaozhi HX, Lin Hongxian, Wu Yuling: **Effect of selenium yeast on serum level of C-peptide in patients with type 2 diabetes mellitus.** *Practical Pharmacy And Clinical Remedies* 2014, **17:**1417-1419

34.	Ye Jingjing CN, Ye Shu, et al.: **Influence of selenium yeast combined with levothyroxine sodium on outcomes in type 2 diabetes mellitus patients complicated with chronic lymphocytic thyroiditis.** *China Journal of Traditional Chinese Medicine and Pharmacy* 2020, **33:**1672-1674

35.	P. Faure OR, A. Favier and S. Halimi: **Selenium supplementation decreases nuclear factor-kappa B activity in peripheral blood mononuclear cells from type 2 diabetic patients.** *European Journal of Clinical Investigation* 2004, **34:**475-481.doi: 10.1111/j.1365-2362.2004.01362.x.

36.	XN M: **Effect of Selenium Supplement on Blood Glucose, Lipid Metabolism and Pregnancy Outcome in Gestational Diabetes Mellitus.** *Chinese Practical Journal of Rural Doctor* 2022, **29:**60-67.10.3969/j.issn.1672-7185.2022.01.022.

37.	Amirani E: **The effects of selenium plus probiotics supplementation on glycemic status and serum lipoproteins in patients with gestational diabetes mellitus: A randomized, double-blind, placebo-controlled trial.** *Clin Nutr ESPEN* 2022, **48:**56-62.10.1016/j.clnesp.2022.02.010.

38.	Yigit E: **Selenium Supplementation and Gestational Diabetes: A Randomised Controlled Trial.** *J Coll Physicians Surg Pak* 2024, **34:**561-567.10.29271/jcpsp.2024.05.561.

39.	Sadat Najib F: **Effects of selenium supplementation on glucose homeostasis in women with gestational diabetes mellitus: A randomized, controlled trial.** *Int J Reprod Biomed* 2019, **18:**57-64.10.18502/ijrm.v18i1.6201.

40.	Asemi Z: **Effects of selenium supplementation on glucose homeostasis, inflammation, and oxidative stress in gestational diabetes: Randomized, double-blind, placebo-controlled trial.** *Nutrition* 2015, **31:**1235-1242.10.1016/j.nut.2015.04.014.

41.	Saifi H: **Influence of selenium supplementation on carbohydrate metabolism and oxidative stress in pregnant women with gestational diabetes mellitus.** *J Med Biochem* 2020, **39:**191-198.10.2478/jomb-2019-0034.

42.	Li Li QJ: **Influence of selenium supplementation for treating patients with polycystic ovary syndrome on their asymmetric dimethyl arginine level and their cardiac metabolic risk factors.** *Chin J Fam Plann* 2023, **31**.doi: 10.3969/j.issn.1004-8189.2023.02.011.

43.	Salimian M: **The effects of selenium administration on carotid intima-media thickness and metabolic status in diabetic hemodialysis patients: A randomized, double-blind, placebo-controlled trial.** *Clin Nutr ESPEN* 2022, **47:**58-62.10.1016/j.clnesp.2021.11.022.

44.	Raygan F: **Selenium supplementation lowers insulin resistance and markers of cardio-metabolic risk in patients with congestive heart failure: a randomised, double-blind, placebo-controlled trial.** *British Journal of Nutrition* 2018, **120:**33-40.10.1017/s0007114518001253.

45.	Zadeh Modarres S: **The effects of selenium supplementation on glycemic control, serum lipoproteins and biomarkers of oxidative stress in infertile women diagnosed with polycystic ovary syndrome undergoing in vitro fertilization: A randomized, double-blind, placebo-controlled trial.** *Clin Nutr ESPEN* 2022, **51:**92-96.10.1016/j.clnesp.2022.07.017.

46.	Tamtaji OR: **Probiotic and selenium co-supplementation, and the effects on clinical, metabolic and genetic status in Alzheimer's disease: A randomized, double-blind, controlled trial.** *Clin Nutr* 2019, **38:**2569-2575.10.1016/j.clnu.2018.11.034.

47.	Mohammad Hosseinzadeh F: **Effects of selenium supplementation on glucose homeostasis and free androgen index in women with polycystic ovary syndrome: A randomized, double blinded, placebo controlled clinical trial.** *J Trace Elem Med Biol* 2016, **34:**56-61.10.1016/j.jtemb.2016.01.002.

48.	Mesdaghinia E: **Clinical and Metabolic Response to Selenium Supplementation in Pregnant Women at Risk for Intrauterine Growth Restriction: Randomized, Double-Blind, Placebo-Controlled Trial.** *Biol Trace Elem Res* 2017, **178:**14-21.10.1007/s12011-016-0911-0.

49.	H C: **To observe the clinical effect of improved resistance to blood glucose levels in patients with abnormal glucose selenium.** 2014

50.	Bahmani F: **Effect of Selenium Supplementation on Glycemic Control and Lipid Profiles in Patients with Diabetic Nephropathy.** *Biol Trace Elem Res* 2016, **172:**282-289.10.1007/s12011-015-0600-4.

51.	Jamilian M: **Metabolic response to selenium supplementation in women with polycystic ovary syndrome: a randomized, double‐blind, placebo‐controlled trial.** *Clinical Endocrinology* 2015, **82:**885-891.10.1111/cen.12699.

52.	Kamali A: **Effects of Selenium Supplementation on Metabolic Status in Patients Undergoing for Coronary Artery Bypass Grafting (CABG) Surgery: a Randomized, Double-Blind, Placebo-Controlled Trial.** *Biol Trace Elem Res* 2019, **191:**331-337.10.1007/s12011-019-1636-7.

53.	Zamani B: **Effects of Selenium Supplementation on the Indices of Disease Activity, Inflammation and Oxidative Stress in Patients with Rheumatoid Arthritis: a Randomized Clinical Trial.** *Biological Trace Element Research* 2023, **202:**1457-1467.10.1007/s12011-023-03782-1.

54.	Wanɡ Bo JX, Qin Liqiánɡ, Yin Xuebin, Lu Xiaoqi, Ma Lianɡcai, Shen Jie, Ni Chuanminɡ, Fenɡ Li, Xue Minjun: **The Effects of Selenium-enriched Corn on Blood Selenium, Fasting Blood Glucose and Glutathione Peroxidase in Middle-aged and Elderly People.** *Journal Of Hygiene Research* 2015, **44:**490-493.10.19813/j.cnki.weishengyanjiu.2015.03.031.

55.	Bahmani F: **The effects of selenium supplementation on biomarkers of inflammation and oxidative stress in patients with diabetic nephropathy: a randomised, double-blind, placebo-controlled trial.** *Br J Nutr* 2016, **116:**1222-1228.10.1017/S0007114516003251.

56.	Deng Shunyou CX, Wu Linying, et al.: **Influence of Selenium on Hashimoto Thyroiditis with Normal Thyroid Function.** *Department of Endocrinology* 2013, **16**.10. 3969 / j. issn. 1007 － 9572. 2013. 07. 089.

57.	Tauil RB, Golono PT, de Lima EP, de Alvares Goulart R, Guiguer EL, Bechara MD, Nicolau CCT, Yanaguizawa Junior JL, Fiorini AMR, Mendez-Sanchez N, et al: **Metabolic-Associated Fatty Liver Disease: The Influence of Oxidative Stress, Inflammation, Mitochondrial Dysfunctions, and the Role of Polyphenols.** *Pharmaceuticals (Basel)* 2024, **17**.10.3390/ph17101354.

58.	EFSA NDA Panel (EFSA Panel on Dietetic Products NaA: **Scientific Opinion on Dietary Reference Values for selenium.** *EFSA Journal* 2014, **12**.doi: 10.2903/j.efsa.2014.3846.

59.	Institute of Medicine Panel on Dietary A, Related C: **Dietary Reference Intakes for Vitamin C, Vitamin E, Selenium, and Carotenoids.** In *Dietary Reference Intakes for Vitamin C, Vitamin E, Selenium, and Carotenoids.* Washington (DC): National Academies Press (US) Copyright 2000 by the National Academy of Sciences. All rights reserved.; 2000

60.	Council NHaMR: *Nutrient Reference Values for Australia and New Zealand Including Recommended Dietary Intakes.* National Health and Medical Research Council; 2006.

61.	Balali A, Sadeghi O, Khorvash F, Rouhani MH, Askari G: **The effect of selenium supplementation on oxidative stress, clinical and physiological symptoms in patients with migraine: a double-blinded randomized clinical trial.** *Front Nutr* 2024, **11:**1369373.10.3389/fnut.2024.1369373.

62.	Tian X: **Selenium Supplementation May Decrease Thyroid Peroxidase Antibody Titer via Reducing Oxidative Stress in Euthyroid Patients with Autoimmune Thyroiditis.** *Int J Endocrinol* 2020, **2020:**9210572.10.1155/2020/9210572.

63.	Santos AC, Passos AFF, Holzbach LC, Cominetti C: **Selenium Intake and Glycemic Control in Young Adults With Normal-Weight Obesity Syndrome.** *Front Nutr* 2021, **8:**696325.10.3389/fnut.2021.696325.

64.	Bizerea-Moga TO, Pitulice L, Bizerea-Spiridon O, Moga TV: **Exploring the Link between Oxidative Stress, Selenium Levels, and Obesity in Youth.** *Int J Mol Sci* 2024, **25**.10.3390/ijms25137276.

65.	Panic A, Stanimirovic J, Sudar-Milovanovic E, Isenovic ER: **Oxidative stress in obesity and insulin resistance.** *Exploration of Medicine* 2022**:**58-70.10.37349/emed.2022.00074.

66.	Kaneto H, Katakami N, Matsuhisa M, Matsuoka TA: **Role of reactive oxygen species in the progression of type 2 diabetes and atherosclerosis.** *Mediators Inflamm* 2010, **2010:**453892.10.1155/2010/453892.

67.	Tanti JF, Jager J: **Cellular mechanisms of insulin resistance: role of stress-regulated serine kinases and insulin receptor substrates (IRS) serine phosphorylation.** *Curr Opin Pharmacol* 2009, **9:**753-762.10.1016/j.coph.2009.07.004.

68.	Wang N, Tan HY, Li S, Xu Y, Guo W, Feng Y: **Supplementation of Micronutrient Selenium in Metabolic Diseases: Its Role as an Antioxidant.** *Oxid Med Cell Longev* 2017, **2017:**7478523.10.1155/2017/7478523.

69.	Chen Z, Tian R, She Z, Cai J, Li H: **Role of oxidative stress in the pathogenesis of nonalcoholic fatty liver disease.** *Free Radic Biol Med* 2020, **152:**116-141.10.1016/j.freeradbiomed.2020.02.025.

70.	Larissa Cristina Fontenelle MMF, Jennifer Beatriz Silva Morais, et al. : **The role of selenium in insulin resistance.** *Brazilian Journal of Pharmaceutical Sciences* 2018, **54**.doi: 10.1590/s2175-97902018000100139.

71.	Zhao Z, Barcus M, Kim J, Lum KL, Mills C, Lei XG: **High Dietary Selenium Intake Alters Lipid Metabolism and Protein Synthesis in Liver and Muscle of Pigs.** *J Nutr* 2016, **146:**1625-1633.10.3945/jn.116.229955.

72.	Liu G, Yu H, Wang C, Li P, Liu S, Zhang X, Zhang C, Qi M, Ji H: **Nano‑selenium supplements in high-fat diets relieve hepatopancreas injury and improve survival of grass carp Ctenopharyngodon Idella by reducing lipid deposition.** *Aquaculture* 2021, **538:**736580.10.1016/j.aquaculture.2021.736580.

73.	Rashidi BH: **Effects of Selenium Supplementation on Asymmetric Dimethylarginine and Cardiometabolic Risk Factors in Patients with Polycystic Ovary Syndrome.** *Biol Trace Elem Res* 2020, **196:**430-437.10.1007/s12011-019-01954-6.

74.	Razavi M: **Selenium Supplementation and the Effects on Reproductive Outcomes, Biomarkers of Inflammation, and Oxidative Stress in Women with Polycystic Ovary Syndrome.** *Horm Metab Res* 2016, **48:**185-190.10.1055/s-0035-1559604.

75.	Mahmoodianfard S: **Effects of Zinc and Selenium Supplementation on Thyroid Function in Overweight and Obese Hypothyroid Female Patients: A Randomized Double-Blind Controlled Trial.** *J Am Coll Nutr* 2015, **34:**391-399.10.1080/07315724.2014.926161.

76.	Hu Y: **Effect of selenium on thyroid autoimmunity and regulatory T cells in patients with Hashimoto's thyroiditis: A prospective randomized-controlled trial.** *Clin Transl Sci* 2021, **14:**1390-1402.10.1111/cts.12993.

77.	Andrzej Adamowicz UT, Anna Trafikowska, Bronisław A. Zachara, Jacek Manitius: **Effect of erythropoietin therapy and selenium supplementation on selected antioxidant parameters in blood of uremic patients on long term hemodialysis.** *Med Sci Monit* 2002, **8:**202-205.PMID: 11887037.

78.	Atapour A: **Effect of Selenium on Triglyceride and Total Cholesterol, Weight Gain, and Physical Activity on Hemodialysis Patients: A Randomized Double-Blinded Controlled Trial.** *Int J Prev Med* 2022, **13:**63.10.4103/ijpvm.IJPVM_378_20.

79.	XQ W: **Clinical efficacy of selenious yeast tablets in the treatment of Hashimoto's thyroiditis and their impact on oxidative and antioxidant system indicators.** *Chin Jof Clinical Rational Drug Use* 2023, **16:**39-41.doi: 10.15887/j.cnki.13-389/r.2023.16.010.

80.	JP Z: **The effect of sodium selenite on the serum TPO-Ab titer and oxidative stress level in patients with hypothyroidism caused by Hashimoto's thyroiditis.** *Labeled Immunoassays & Clin Med* 2022, **29:**32-35.doi: 10.11748/bjmy.issn.1006-1703.2022.01.008.

81.	CM Q: **Effects of selenium supplements on thyroid function, immune response and oxidative stress in HT patients.** 2019

82.	Ren Zhao BM, Guo Chizhang, Min Huazheng, Dong Huali, Liang Huang, Hong Zhizou: **Effect of selenium on immunity and anti-oxidative functions in patients with colorectal carcinoma.** *Shijie Huaren Xiaohua Zazhi* 2000, **8:**1013-1016.ISSN: 1009-3079.

83.	JJ Y: **Synergistic effect of Selenious Yeast Tablets combined with Danggui Yinzi on chronic urticaria and regulating effect on serumal malondialdehyde.** 2018

84.	Xu Jinliang LL, Wang Jianhua, et al.: **Effects of Selenium Yeast on Serum Nitric Oxide and High-sensitivity C-reactive Protein in Patients with Alzheimer Disease.** *Chinese Journal of Medicinal Guide* 2012, **14:**83-84.doi: 1009-0959(2012)01-0083-02.
